# Activation of metabolic and stress responses during subtoxic expression of the type I toxin *hok* in *Erwinia amylovora*

**DOI:** 10.1186/s12864-021-07376-w

**Published:** 2021-01-22

**Authors:** Jingyu Peng, Lindsay R. Triplett, George W. Sundin

**Affiliations:** 1 Department of Plant, Soil, and Microbial Sciences, Michigan State University, East Lansing, MI USA; 2 Department of Plant Pathology and Ecology, The Connecticut Agricultural Experiment Station, New Haven, CT USA

**Keywords:** Toxin:antitoxin, Fire blight, Phage shock protein, Transcriptome, Antibiotic tolerance

## Abstract

**Background:**

Toxin-antitoxin (TA) systems, abundant in prokaryotes, are composed of a toxin gene and its cognate antitoxin. Several toxins are implied to affect the physiological state and stress tolerance of bacteria in a population. We previously identified a chromosomally encoded *hok*-*sok* type I TA system in *Erwinia amylovora*, the causative agent of fire blight disease on pome fruit trees. A high-level induction of the *hok* gene was lethal to *E. amylovora* cells through unknown mechanisms. The molecular targets or regulatory roles of Hok were unknown.

**Results:**

Here, we examined the physiological and transcriptomic changes of *Erwinia amylovora* cells expressing *hok* at subtoxic levels that were confirmed to confer no cell death, and at toxic levels that resulted in killing of cells. In both conditions, *hok* caused membrane rupture and collapse of the proton motive force in a subpopulation of *E. amylovora* cells. We demonstrated that induction of *hok* resulted in upregulation of ATP biosynthesis genes, and caused leakage of ATP from cells only at toxic levels. We showed that overexpression of the phage shock protein gene *pspA* largely reversed the cell death phenotype caused by high levels of *hok* induction. We also showed that induction of *hok* at a subtoxic level rendered a greater proportion of stationary phase *E. amylovora* cells tolerant to the antibiotic streptomycin.

**Conclusions:**

We characterized the molecular mechanism of toxicity by high-level of *hok* induction and demonstrated that low-level expression of *hok* primes the stress responses of *E. amylovora* against further membrane and antibiotic stressors.

**Supplementary Information:**

The online version contains supplementary material available at 10.1186/s12864-021-07376-w.

## Background

Toxin-antitoxin (TA) systems are simple genetic loci that encode a stable proteinaceous toxin and an unstable counteracting antitoxin. TA systems are widely found throughout the chromosomes and plasmids of free-living prokaryotes [1]. In type I TA systems, the antitoxins are small RNAs that inhibit the translation of or facilitate the degradation of the transcript encoding the corresponding toxin (reviewed in [2, 3]). Type I toxins, such as Hok, HokB, and TisB, tend to be small (≤60 amino acids) hydrophobic proteins containing one transmembrane domain [4–6]. A high induction level of the toxin genes *hok* or *tisB* causes drastic cell death of *E. coli* cells, accompanied by collapse of the proton motive force (PMF) [7–9]. The gene products of both *hokB* and *tisB* form membrane pores in *Escherichia coli* [8, 10] and lead to leakage of cellular ATP during moderate [10] or high-level [7] induction of the toxin genes. The PMF, the proton gradient generated via oxidation of NADH and FADH_2_, is required to generate ATP through ATP synthase, as well as to power membrane-localized cell machinery, such as the flagellum [11, 12]. The *hok/sok* TA system in *E. coli* has been suggested as a target for killing host bacterial cells [13, 14]. Through sequestering the sRNA *sok* from interacting with *hok* mRNA by addition of anti-Sok peptide nucleic acid (PNA) oligomers [13] or doxycycline that inhibits RNase III degradation of the *hok*-*sok* dsRNA complex, *hok* mRNA is released and consequently causes cell death [14].

The molecular targets and regulatory roles of many TA systems are still enigmatic. Although inactivation of a single type I TA system does not frequently result in a phenotype [15], studies using low-level ectopic expression have revealed that a few membrane-associated TA systems can affect the physiological state and stress tolerance of bacteria in a population. In *E. coli*, expression of *hokB* or *tisB* at sub-toxic levels increased the proportion of persister cells with tolerance to multiple antibiotics, which was hypothesized to result from growth retardation following ATP leakage and the loss of the PMF [7, 8, 15–17]. Plasmid expression of the *hok-sok* locus also increased T4 bacteriophage exclusion in *E. coli* [18]. Interestingly, despite its role in compromising membrane integrity, moderate *hokB* expression was observed to increase metabolic activity in *E. coli*, determined via a fluorescent redox sensor [10].

Through transcriptomics and in vitro RNA degradation analyzes, Wang et al. demonstrated that the type V antitoxin GhoS cleaves the membrane-associated toxin *ghoT* mRNA [19]. However, the global transcriptional effects of a type I membrane-associated TA, to the best of our knowledge, have not been previously examined. It has been hypothesized that induction of *hokB* may activate phage shock protein (*psp*) genes, based on the protective effects of Psp proteins in mitigating various membrane stresses in *E. coli* [20, 21]*.* Though the effects vary in different bacteria, perturbation of the cell membrane seems to cause shared consequences in activating stress responses and downregulating genes that encode energy consuming machinery [22–26]. Addition of polymyxin, an antibiotic that causes formation of membrane pores and cell death in bacteria, caused increased expression of genes associated with vancomycin resistance and decreased expression of virulence factor-related genes in *Staphylococcus aureus* [22]; exposure of *Klebsiella pneumoniae* to 1-(1-Naphthylmethyl)-piperazine depolarized the membrane PMF yet upregulated many envelope stress response genes [26]. Still, it is not known whether endogenous pore-forming toxins also trigger stress response or influence the expression of virulence genes.

Recently, we identified a chromosomally encoded *hok*-*sok* type I TA system in *Erwinia amylovora* [27], a model enterobacterial plant-pathogenic bacterium that causes the destructive fire blight disease of pome fruit trees including apple (*Malus* sp.) and pear (*Pyrus* sp.) [28, 29]. Episomal overexpression of the *hok* gene caused massive killing of *E. amylovora* cells and arrested cell division after septa were formed [27]. We proposed that cell death due to *hok* induction at toxic levels in *E. amylovora* is likely to be associated with the disturbance of essential functions of the cell membrane. Although upregulation of toxin genes occurs under a variety of different stress conditions [30–34], natively expressed toxin genes are not known to be induced to cell-killing levels in any environmental context, to the best of our knowledge. Therefore, we hypothesized that *hok* might actually confer a selective advantage to *E. amylovora* at moderate (subtoxic) levels of induction, when no cell death is observed. In this study, we compared the transcriptome profiles of *E. amylovora* cultures expressing *hok* at toxic, subtoxic, and wild-type levels. We found that Hok plays important roles in activating ATP biosynthesis and priming the tolerance of *E. amylovora* cells against membrane and antibiotic damage.

## Results

### Moderate overexpression of *hok* does not suppress bacterial growth

**Fig. 1:**
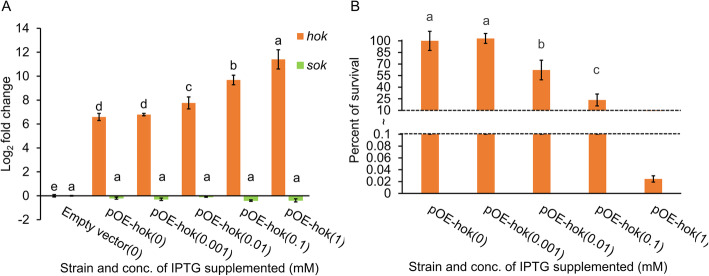
Induction of *hok* and its effect on cell survival in *E. amylovora*. **a** Expression levels of *hok* induced with four progressive doses (0.001 mM, 0.01 mM, 0.1 mM, and 1 mM) of isopropyl β-D-1-thiogalactopyranoside (IPTG) or with water. **b** The effect *hok* induction on survival rate of *E. amylovora*. The concentrations of IPTG supplemented are indicated in parentheses. After IPTG or water addition, cultures were incubated at 28 °C with 200 rpm shaking for 1 h. Expression levels of *hok* were measured using quantitative real-time PCR (qRT-PCR), and fold changes were calculated using the 2^-ΔΔ*C*^_T_ formula. The *recA* gene was used as an endogenous control. Survival rate was determined as the ratio of colony forming units (CFU)/ml in Ea1189(pOE-*hok*) after and before the addition of IPTG. Results represent the means of three replications, and error bars indicate the standard deviation. Different letters indicate significant differences (*P* < 0.05) using Tukey’s HSD (honestly significant difference) test. The experiments were conducted three times with similar results

A *hok* overexpression construct, pOE-*hok*, was previously generated by cloning the *E. amylovora* Ea1189 *hok* gene into the *lac* promoter-containing plasmid pEVS143 [27]. The *lac* promoter allows low levels of transcription in the absence of the inducer isopropyl β-D-1-thiogalactopyranoside (IPTG) [35]. We did not observe any growth defect in *E. amylovora* Ea1189 cells transformed with pOE-*hok* (Fig. S1), suggesting that *E. amylovora* is able to tolerate leaky *hok* expression without inhibiting growth. Therefore, we hypothesized that Ea1189(pOE-*hok*) grown in the absence of IPTG induction may provide a useful system to identify the physiological roles of Hok separate from those caused by its toxicity. We used quantitative real-time PCR (qRT-PCR) to measure the expression levels of *hok* in Ea1189(pEVS143) and Ea1189(pOE-*hok*) without IPTG and in four progressively increasing doses of IPTG, and monitored the growth of the cultures in the same conditions. In the absence of IPTG, expression of *hok* was approximately 40-fold higher in Ea1189(pOE-*hok*) compared to Ea1189(pEVS143), and expression of *hok* increased by another ~ 130-fold when 1 mM IPTG was added to the Ea1189(pOE-*hok*) culture (Fig. 1a). The expression levels of the small RNA antitoxin *sok* remained almost unchanged in these conditions (Fig. 1a). Induction of *hok* did not result in cell death until expression reached about 60-fold induction or greater, induced by the addition of 0.01 mM IPTG (Fig. 1b). Henceforth, we will define *hok* expression from the *lac* promoter with 0.01 mM, 0.1 mM or 1 mM IPTG as the “toxic” expression conditions for this study, while expression from the *lac* promoter with 0.001 mM or no IPTG will be defined as the “subtoxic” expression conditions.

### Induction of *hok* causes PMF collapse and membrane rupture

**Fig. 2:**
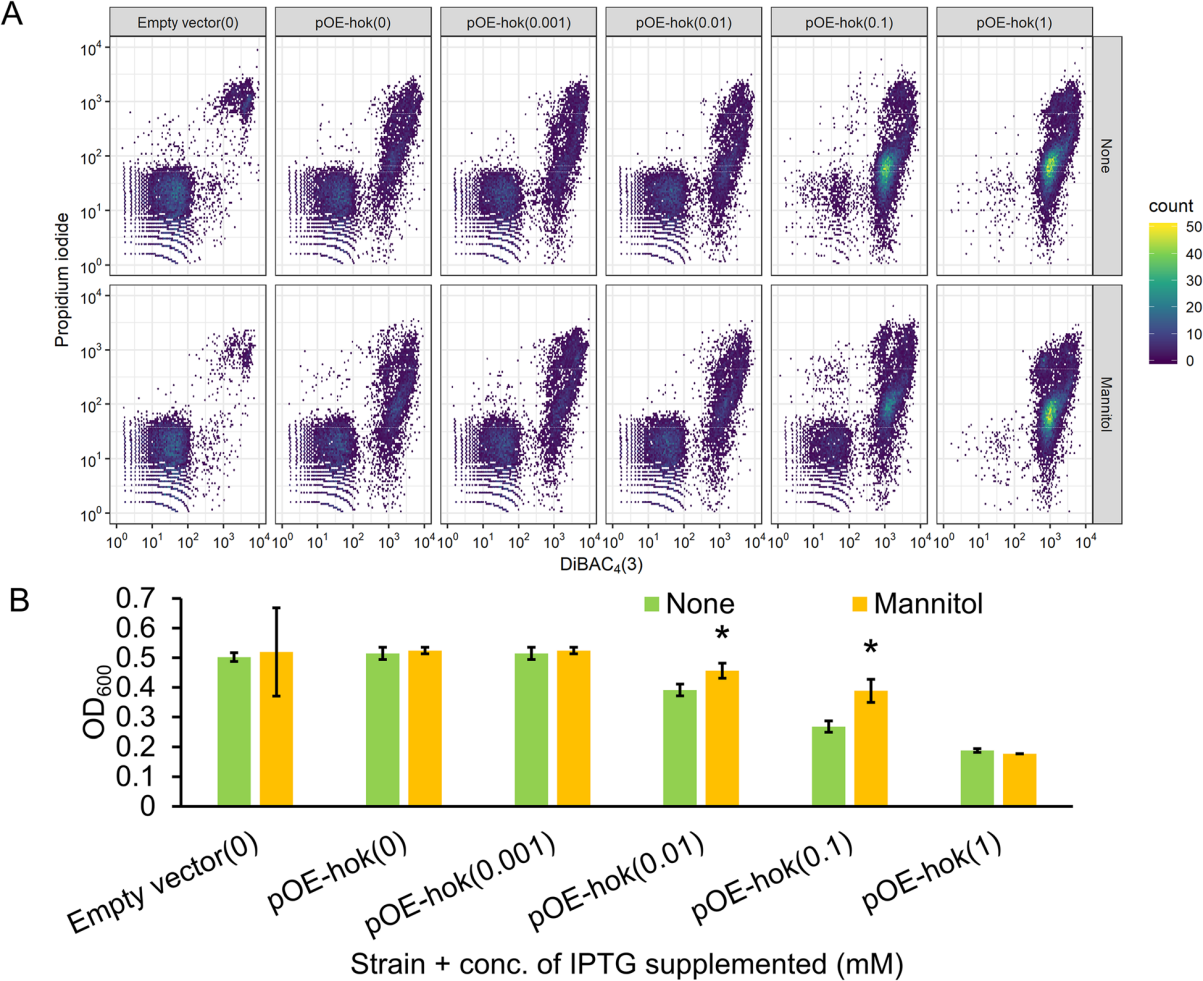
*hok* induction disturbs essential membrane functions of *E. amylovora*. Effect of *hok* induction on the proton motive force (PMF) and membrane integrity without (panel labelled as “None”) or with the addition of 10 mM mannitol (panel labelled as “Mannitol”) immediately before IPTG supplementation (**a**), and effect of mannitol in reversing the toxicity of Hok (**b**). *E. amylovora* cultures grown overnight for 20 h in LB broth were washed twice and diluted to OD_600_ = 0.2 in fresh LB broth. The concentrations of IPTG supplemented are indicated in parentheses. After incubation at 28 °C with 200 rpm shaking for 1 h, the PMF of cultures was examined using bis-(1,3-dibutylbarbituric acid) trimethine oxonol (DiBAC_4_ [3]), and membrane integrity was determined via propidium iodide (PI). Fluorescence was measured using a BD LSR II flow cytometer. Ten thousand events were examined with a 488 nm laser and a 530/30 emission filter (DiBAC_4_ [3]) staining and a 561 nm laser and a 620/15 emission filter (PI). Subsequent analyses were conducted on Flowing Software 2.5.1 and R v3.4.0. Increased fluorescence after treatment with DiBAC_4_ [3] or PI indicates greater collapse of the PMF or compromised membrane integrity, respectively. To test the effect of bacterial metabolites on the toxicity of Hok, 10 mM mannitol or 10 mM arabinose was added to the cultures (OD_600_ = 0.2) immediately before IPTG was added, and the OD_600_ was measured 4 h after the incubation using a Tecan spectrophotometer. Bacterial growth was monitored by measuring OD_600_ of the cultures using a Tecan spectrophotometer. Results represent the means of three biological replicates and error bars indicate the standard deviation. Different letters indicate significant differences (*P* < 0.05) using Student’s *t*-test. The assays were done three times with similar results

Membrane-associated type I toxins of *E. coli*, including HokB and TisB, form membrane pores [8, 10, 19], and cause collapse of the PMF [9, 10, 17]. We therefore wondered if the transmembrane domain-containing *E. amylovora* Hok, sharing 48 and 14% amino acid identity to HokB and TisB, respectively, also causes membrane depolarization and rupture. To assess this possibility, we measured membrane potential using DiBAC_4_ [3] (bis-(1,3-dibutylbarbituric acid) trimethine oxonol), a membrane potential-sensitive fluorescent dye. Fluorescence level negatively correlates to membrane potential, meaning that higher fluorescence indicates a greater level of PMF collapse. Carbonyl cyanide-m-chlorophenylhydrazone (CCCP), a protonophore that uncouples the PMF, was used as a positive control for the DiBAC_4_ [3] staining (Fig. S2). Propidium iodide (PI) was used as an indicator of membrane rupture, which binds to nucleic acid and generates fluorescence in membrane integrity compromised cells. Ethanol disturbs the physical structure of cell membranes and was used as a positive control for the PI staining (Fig. S2). Fluorescence was measured in single cells using a flow cytometer. We found that induction of *hok* to subtoxic levels caused membrane depolarization and rupture in a subpopulation of cells, though many cells remained unchanged in their membrane states (Fig. 2a)*.* More drastic membrane depolarization and rupture was observed when *hok* was induced to toxic levels (Fig. 2a). At the highest level of *hok* induction, almost the entire population was shifted to the membrane depolarization state, with varied levels of membrane rupture. We next asked whether mannitol, a bacterial metabolite that feeds into glycolysis and was shown to stimulate the PMF in *E. coli* [36], was able to restore the collapsed PMF and rupture of cell membrane due to the toxicity of Hok in *E. amylovora*. In cells expressing *hok* with 0.1 mM IPTG induction, mannitol partially relieved the membrane stress (Fig. 2a). Similarly, addition of mannitol significantly alleviated the inhibitory effect of bacterial growth during 0.01 or 0.1 mM induction of *hok* (Fig. 2b). However, when 1 mM IPTG was supplemented, the protective effect of mannitol was not observed in any of these phenotypes (Fig. 2a and Fig. 2b). Arabinose, which does not contribute to the PMF [36], was used a negative control for the assays (Fig. S3).

### Transcriptomic analysis reveals that *hok* overexpression affects genes involved in stress responses and energy generation/consumption

While overexpression of *E. amylovora hok* causes extreme disturbance of essential membrane functions, it is not clear how the membrane disruption capacity of these toxins may affect bacterial physiology when *hok* is expressed in subtoxic or native expression conditions. To distinguish potential downstream effects of *E. amylovora* Hok from those resulting from toxicity, we compared the transcriptomes of *E. amylovora* cultures expressing *hok* at wild-type levels (i.e., wild-type strains carrying the empty vector) with cultures expressing *hok* at subtoxic (IPTG untreated) and toxic (1 mM IPTG treated) levels. Expression of each gene was quantified as counts per million reads (CPM), and differentially-expressed genes (DEGs) were defined as those having greater than 2-fold change of CPM values and less than 0.05 of the corresponding false discovery rate (FDR) values (Fig. S4 and Table S1).

**Fig. 3:**
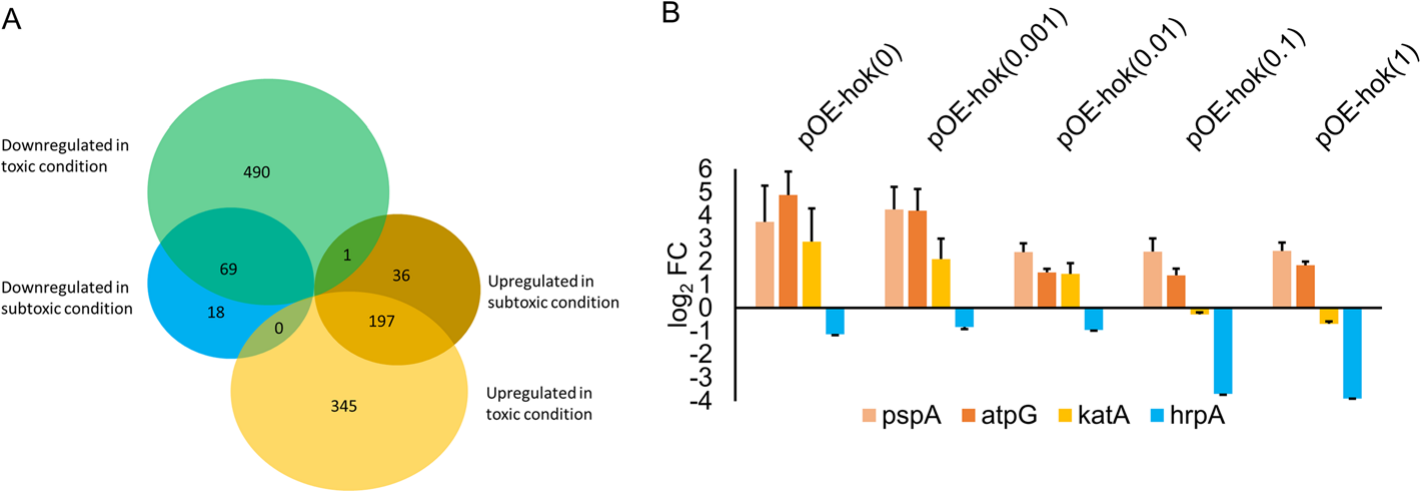
Comparative transcriptomic analysis of *E. amylovora* cells expressing *hok* at wild-type, subtoxic and toxic levels, respectively. **a** Venn diagram of the differentially-expressed genes (DEGs) in *E. amylovora* cells expressing *hok* at subtoxic or toxic level. **b** Expression of representative DEGs in subtoxic or toxic condition examined using qRT-PCR. Fold changes were calculated using the 2^-ΔΔ*C*^_T_ formula. The *recA* gene was used as an endogenous control. The error bars indicate standard deviation. The concentrations of IPTG supplemented are indicated in parentheses

**Fig. 4:**
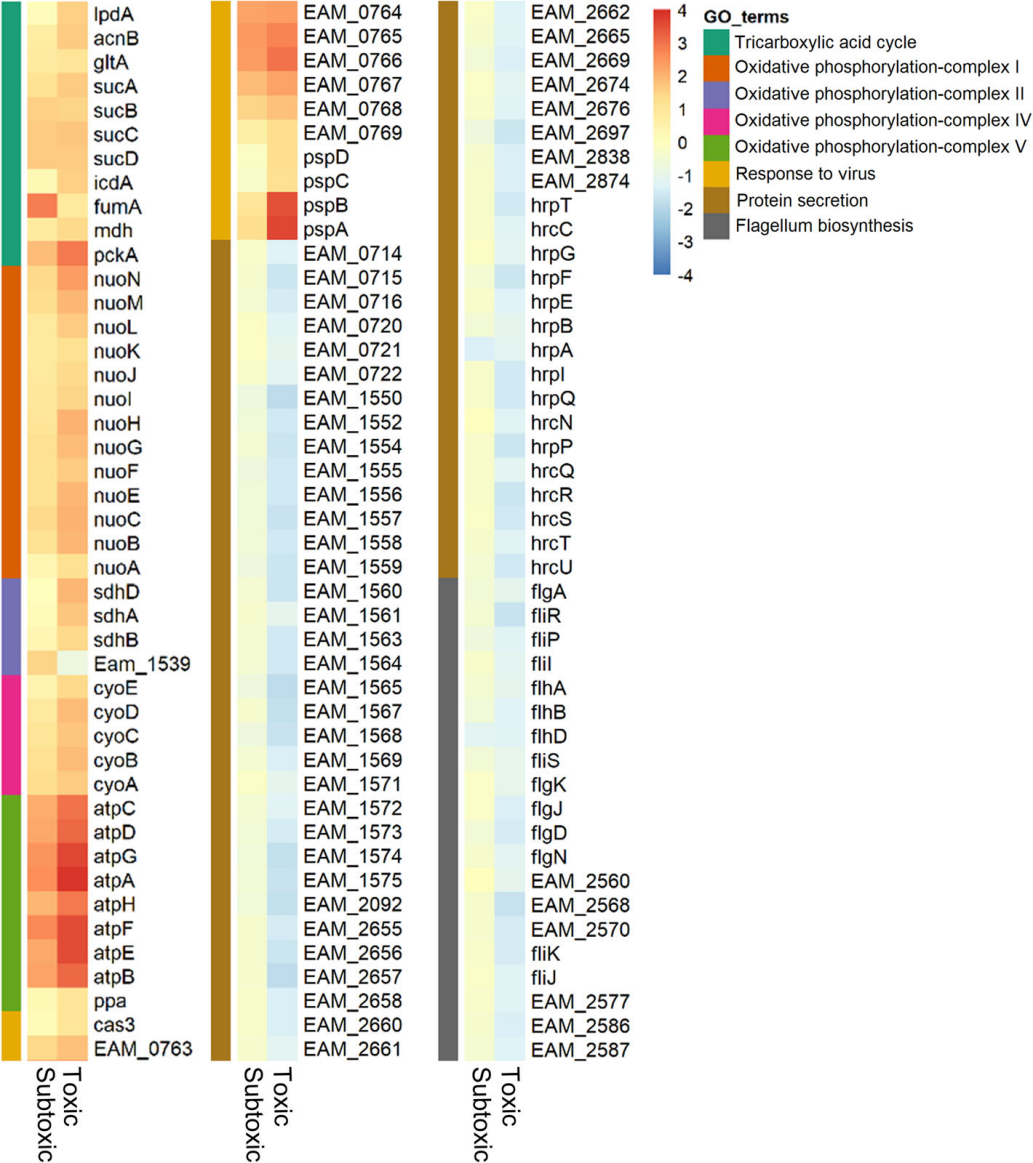
Overrepresented Gene Ontology (GO) terms enriched in the GO enrichment analysis with a cutoff FDR of 0.01. Scale bar indicates the color key of log_2_ fold-change values

Compared with Ea1189(pEVS143), which was also untreated with IPTG, 321 DEGs were identified in IPTG-untreated Ea1189(pOE-*hok*), of which 234 had increased expression and 87 had decreased expression (Fig. 3a). After 1 mM IPTG treatment of Ea1189(pOE-*hok*), a much larger set of 541 and 560 genes were up- and down-regulated, respectively (Fig. 3a). Approximately 83% of the DEGs identified in the subtoxic condition were differentially expressed in the same direction and to a greater extent in the toxic condition. Expression of representative genes in Ea1189(pOE-*hok*) in subtoxic and toxic conditions was validated through qRT-PCR (Fig. 3b). The housekeeping gene *recA* was used as an endogenous control, that had negligible differences in expression among *E. amylovora* cultures expressing wild-type, subtoxic, or toxic levels of *hok* in our transcriptomic analysis*.* Based on the read count, the ratio of *hok* to *sok* was approximately 18 in the wild-type condition, that increased to ~ 200 in the subtoxic condition and ~ 6000 in the toxic condition (Fig. S5). Gene ontology (GO) enrichment analysis of the DEGs further revealed that *hok* exerts substantial effects in the essential metabolism of *E. amylovora* (Fig. 4 and Table S2). Oxidative phosphorylation-related genes (GO:0006119), that include NADH-coenzyme Q oxidoreductase (complex I), Succinate-Q oxidoreductase (complex II), Cytochrome c oxidase (complex IV) and F_1_F_o_-ATPase (complex V), were enriched among the higher expressed genes in both toxic and subtoxic conditions. Specifically, in the toxic condition, higher expressed genes were also significantly associated with the “tricarboxylic acid cycle” GO term (GO:0006099).

Several genes with demonstrated importance to bacterial plant pathogenesis were negatively affected by elevated *hok* expression. Specifically, *hrpA* and *flhD*, encoding a T3SS protein and a flagellar transcriptional activator, respectively, decreased in expression at both levels of *hok* induction. In toxic but not subtoxic conditions, down-regulated genes were primarily comprised of flagellar genes and “protein secretion” (GO:0009306) genes, which included type II secretion system (T2SS) and type III secretion system (T3SS)-related genes.

Induction of *hok* also activated multiple genes involved in stress responses. Several genes with known roles in antibiotic persistence and other stress responses, i.e. *groS*, *groL*, *dnaK*, *dnaJ*, *skp*, *surA*, *sucB* and *lon* [37–42], were consistently more highly expressed in both *hok* induction conditions. Also upregulated were genes in the “response to virus” ontology (GO:0009615), including genes encoding phage shock proteins, i.e. *pspABCD*, and CRISPR-associated proteins. The catalase gene *katA* showed increased expression in the subtoxic condition, consistent with our previous observation that catalase activity is significantly compromised in a *hok*-*sok* deletion mutant [27]. The stress-induced ATP-dependent chaperone gene *clpB* was also more highly expressed in the subtoxic but not the toxic condition. Together, these results show that different *hok* expression levels exert diverse and overlapping effects on the *E. amylovora* transcriptome, enhancing expression of metabolic and stress-related traits while suppressing genes required for infection.

### *hok* positively affects ATP biosynthesis

**Fig. 5:**
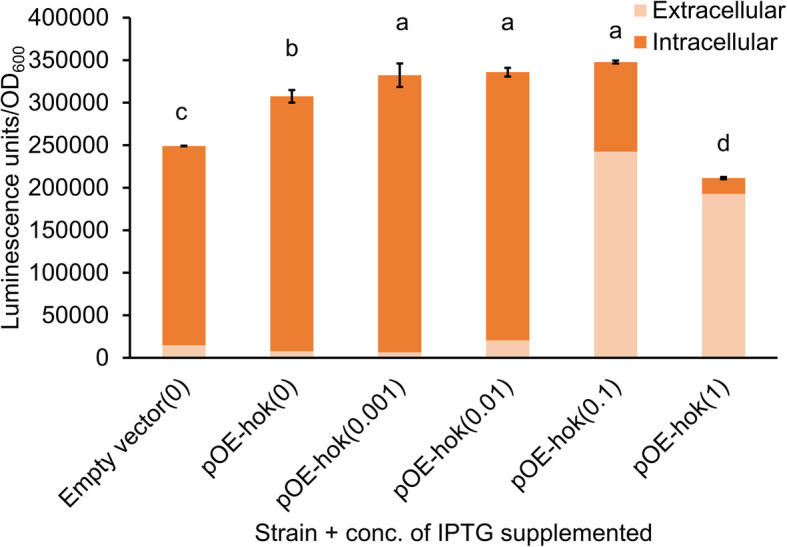
Effect of *hok* induction on ATP biosynthesis in *E. amylovora*. Both extracellular and intracellular levels of ATP were simultaneously quantified using a luciferase reporter system. Results represent the means of three biological replications and error bars indicate the standard deviation. Different letters indicate significant differences (*P* < 0.05) using Tukey’s HSD test. The assays were done twice with similar results

Membrane-associated type I toxins have been shown to cause leakage of cellular ATP as indicated by either decrease level of intracellular ATP or increase level of extracellular ATP [7, 10, 19]. In this study, we found that genes associated with oxidative phosphorylation, the process of ATP generation through electron transfer, were higher expressed in the subtoxic condition and were higher expressed to a greater extent in the toxic condition (Fig. 5). We hypothesized that the upregulation of ATP biogenesis-related genes could be part of a response to compensate for the possible leakage of intracellular ATP through increased ATP synthesis in Ea1189(pOE-*hok*) cultures in both subtoxic and toxic conditions. To determine whether ATP leakage was occurring, we performed simultaneous measurements of both the intracellular and the extracellular levels of ATP in both subtoxic and toxic conditions. When induced with 0.1 or 1 mM IPTG, conditions causing more than 70% dieoff (Fig. 1b), *E. amylovora* Hok caused dramatic leakage of ATP from the cells, indicated by the decreased level of intracellular ATP and increased level of extracellular ATP (Fig. 5 and Fig. S6). In contrast, a significant increase in intracellular ATP was measured after induction with 0.01 mM or less IPTG (Fig. 5 and Fig. S6), expression conditions that were associated with minimal or no cell death of *E. amylovora* (Fig. 1b). No ATP leakage was observed in these subtoxic conditions.

Combining intercellular and extracellular ATP measurements allowed us to assess the total ATP concentration under each expression condition. In the absence of IPTG, total ATP was greater in Ea1189(pOE-*hok*) cultures than Ea1189(pEVS143). Total ATP in Ea1189(pOE-*hok*) increased with IPTG addition at concentrations up to 0.1 mM (Fig. 5). At the highest concentration of IPTG tested, 1 mM, the total ATP in Ea1189(pOE-*hok*) cultures started to decrease compared with lower levels of inducer, likely due to the massive kill-off of ATP-generating cells at this induction level. Taken together, our results suggest that *hok* positively affects the biosynthesis of ATP, and leakage of ATP only occurs when *hok* was induced at toxic levels.

Overexpression of the ATP synthase gene *atpB* is toxic to *E. coli* cells; it allows leakage of protons through the F_0_ sector of F_1_F_o_-ATPase [43–46]. Given that *hok* positively affects ATP synthase gene expression and ATP biosynthesis in subtoxic conditions, we wondered if the toxicity of Hok was increased by the upregulation in ATP synthase genes. To test this hypothesis, we generated ATP synthase gene deletion mutants, Ea1189*ΔatpB* and Ea1189*ΔatpBEFHAGDC*. The growth of Ea1189*ΔatpB* and Ea1189*ΔatpBEFHAGDC* mutants was severely reduced, as overnight cultures only reached OD_600_ ≈ 0.3 compared with OD_600_ ≈ 1.5 in the wild-type Ea1189 strain (data not shown). pOE-*hok* was transformed into the ATP synthase mutants to generate Ea1189*ΔatpB*(pOE-*hok*) and Ea1189*ΔatpBEFHAGDC*(pOE-*hok*), respectively. Hok expression was induced in the wild-type and ATPase mutant backgrounds with 1 mM IPTG, and survival rates were measured. Hok killing efficiency was not changed between the wild-type and the mutants (Fig. S7), suggesting that the toxicity of Hok is not affected by the increased expression of ATP biosynthesis genes.

### Expression of *pspA* is induced in known PMF dissipation conditions and relieves the toxicity of Hok

**Fig. 6:**
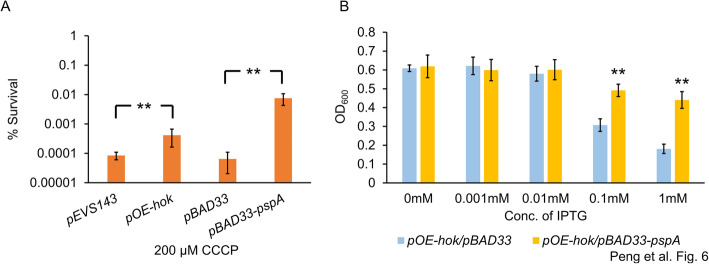
Effect of *pspA* in managing membrane stresses in *E. amylovora*. **a** Carbonyl cyanide-m-chlorophenylhydrazone (CCCP) was added to 200 μM to *E. amylovora* cultures and incubated for 5 h. Survival rate was determined as the ratio of CFU/ml after the treatment to that before the treatment. Arabinose at 10 mM, but no IPTG, was supplemented to the cultures. **b** Overexpression of *pspA* largely reverses the killing of *E. amylovora* cells expressing *hok* at toxic levels. Growth of cultures was determined by measuring OD_600_ using a Tecan spectrophotometer. Arabinose at 10 mM was supplemented to the cultures. Results represent the means of four biological replications and error bars indicate the standard deviation. Asterisk symbols indicate significant differences (*P* < 0.05 using Student’s *t*-test). The assays were done three times with similar results

Our transcriptome results indicated that *psp* genes were upregulated in both expression conditions. The *psp* genes are induced on exposure to conditions that dissipate the PMF, such as bacteriophage infection, alkaline pH, and addition of uncoupling agents, in both Gram-negative and -positive bacteria (reviewed in [47]). The protective roles of PspA in managing membrane stresses have been validated in *E. coli* and *Salmonella enterica* serovar Typhimurium [48–50]. As the functions of *psp* genes have not been previously investigated in *E. amylovora*, we constructed a transcriptional fusion of the promoter region of the *pspABCD* operon to a green fluorescence protein (*gfp*) reporter. As expected, the promoter activity of the *pspABCD* operon was significantly increased in *E. amylovora* cells after exposure to bacteriophage, and was increased to a lesser extent in the presence of CCCP, ethanol, or Triton X-100 (Fig. S8). To examine the possible protective role of *pspA* under the condition of membrane stress in *E. amylovora*, we generated the *pspA-*overexpression construct, pBAD33-*pspA*, through cloning the *pspA* gene into the pBAD33 plasmid, containing the arabinose-inducible P_BAD_ promoter. Compared with Ea1189(pBAD33), Ea1189(pBAD33-*pspA*) cultures were ~ 100 times more tolerant to CCCP (Fig. 6a). Interestingly, without supplementing any IPTG, Ea1189(pOE-*hok*) cultures survived at significantly higher rates than Ea1189(pEVS143) (Fig. 6a), suggesting that induction of *hok* at subtoxic levels protect *E. amylovora* cells from further membrane damage by activating the expression of *pspA*. Interestingly, *pspA* overexpression significantly alleviated the toxicity due to high levels of *hok* induction (Fig. 6b), further validating the defensive role of *pspA* in response to membrane stress in *E. amylovora*.

### Subtoxic expression of *hok* increases tolerance of stationary-phase *E. amylovora* cells to the aminoglycoside antibiotic streptomycin

**Fig. 7:**
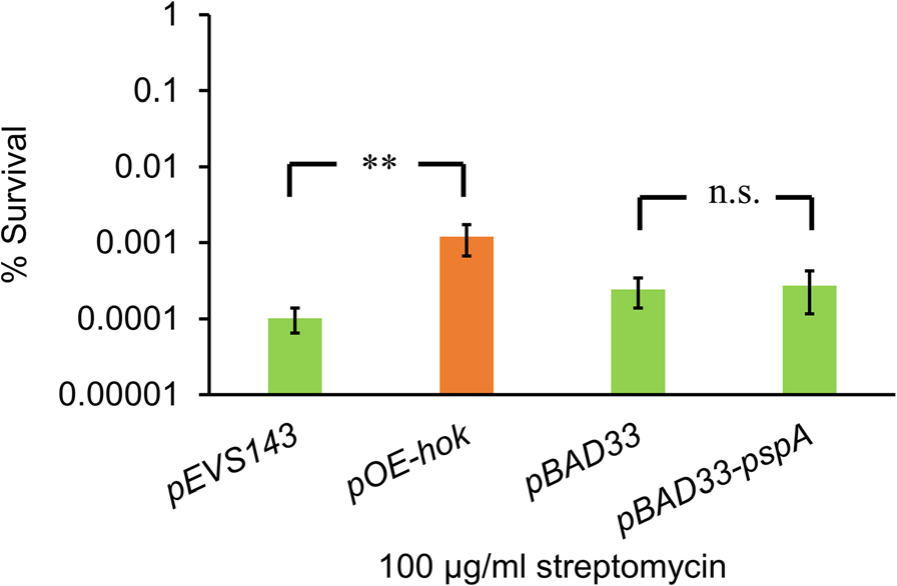
Subtoxic level of *hok* increases tolerance of stationary-phase *E. amylovora* cells to streptomycin. Single colonies of *E. amylovora* cultures were grown in LB broth amended with selective antibiotics for 20 h to reach stationary phase. Cultures were washed twice using fresh LB broth before streptomycin was added to 100 μg/ml; the culture was subsequently incubated at 28 °C with shaking for 5 h. Survival rate was determined as the ratio of CFU/ml after the treatment to that before the treatment. Arabinose at 10 mM and no IPTG was supplemented into the cultures. Results represent the means of four biological replications and error bars indicate the standard deviation. Asterisk symbols indicate significant differences (*P* < 0.05 using Student’s *t*-test). The assays were done three times with similar results

Transcriptome results showed that *hok* expression upregulated several genes previously associated with antibiotic persistence, so we next asked whether *hok* has a role in antibiotic tolerance during stationary phase. Without addition of IPTG, stationary phase *E. amylovora* cultures expressing *hok* had 10 times the number of survivors to streptomycin exposure than the vector control strain (Fig. 7). concentration that is routinely used for management of fire blight and screening of streptomycin-resistant *E. amylovora* isolates [51–53]. Of note, we did not observe altered tolerance of *E. amylovora* cultures overexpressing *pspA*, suggesting that *hok* does not affect antibiotic tolerance through overproduction of PspA.

## Discussion

In this study, by taking advantage of the “leaky” expression of the *lac* promoter, we showed that induction of the type I toxin *hok* disturbs essential functions of the cell membrane in both subtoxic and toxic conditions. In an effort to understand the physiological roles of *hok* in subtoxic and native expression conditions, we examined the transcriptomic changes in *E. amylovora* cells expressing *hok* at subtoxic and toxic levels. We demonstrated that subtoxic expression of *hok* in *E. amylovora* stimulates ATP biogenesis, activates *pspA* expression to protect cells from further membrane damage, and renders stationary phase cell cultures more tolerant to streptomycin.

Consistent with observations in *E. coli* expressing *tisB* at a toxic level or *hokB* at a subtoxic level, induction of *E. amylovora hok* at both subtoxic and toxic levels caused collapse of the PMF and membrane rupture in a subpopulation of the cultures. Interestingly, although induction of *hok* with 1 mM IPTG killed more than 97% of *E. amylovora* cells, a significant subpopulation of cells retained low levels of membrane rupture under the same conditions. Therefore, the culturability of *E. amylovora* cells expressing a toxic level of *hok* is not entirely correlated with the level of membrane rupture. Addition of mannitol alleviated the toxicity of Hok at lower induction levels, indicating that collapse of the PMF indeed contributes to the toxicity of Hok. However, mannitol did not reduce toxicity when Hok was expressed to the highest levels. Mannitol provides the PMF through glycolysis, and the massive killing due to high-level *hok* induction may severely impair the central metabolism machineries of *E. amylovora*.

Our transcriptomic profiling experiments demonstrate that induction of *hok* positively affects the expression of all the F_1_F_o_-ATPase genes. F_1_F_o_-ATPase, consist of a proton-conducting structure and a catalytic portion, exhibits a central role in energy transduction in bacteria. In line with the transcriptomic changes, quantification of intracellular and extracellular ATP showed that induction of *hok* positively affects ATP biogenesis, though leakage of ATP from cells was observed in toxic conditions. Although leakage of cellular ATP has also been suggested in a few other membrane-associated toxins at toxic conditions in *E. coli*, only intracellular [7, 54] or extracellular ATP [10] alone was measured.

Though a subtoxic level of *hok* triggered primarily higher expression of genes in *E. amylovora*, a greater proportion of DEGs were negatively affected in cells expressing toxic levels of *hok*, including genes of flagellum, type II secretion system, and type III secretion system. A previous study of a Lon protease mutant in *E. amylovora* showed that Lon negatively affects the expression of pathogenesis-related genes *hrpA* and *flhD* [55]. In this study, *lon* expression was increased, while *hrpA* and *flhD* expression was reduced, by more than two-fold in both *hok* expression conditions. This suggests that induction of *hok* inactivates these energy-consuming cell machineries that are important for the pathogenesis of *E. amylovora,* and this favors energy conservation during the stress.

The transcriptome analysis in this study showed that *hok* triggers higher expression of *psp* genes in *E. amylovora*. Though not previously studied in *E. amylovora*, Psp proteins and homologs are widely found in bacteria, archaea, and plants [47]. Bacterial Psp proteins are activated in response to phage, extreme temperature, ethanol, mislocalization of outer membrane secretins, and other events that cause membrane dissipation [23, 30, 31, 56–61]. We demonstrated that the promoter activity of *E. amylovora pspA* was induced upon exposure to several membrane stress-inducing substances, and that overexpression of *pspA* increased tolerance to Hok. These results show that in addition to protection from external stresses, PspA can serve a protective function from the effects of bacterially produced stresses, such as toxin-antitoxin systems.

We found that subtoxic *hok* induction increased tolerance of stationary cultures of *E. amylovora* to the aminoglycoside antibiotic streptomycin. This is consistent with a previous report that low-level expression of the hok homolog *hokB* confers increased antibiotic tolerance to *E. coli* [10]. Although energy state is an important factor for persister development in different bacteria [34, 62], we did not observe any leakage of ATP from *E. amylovora* cells expressing subtoxic levels of *hok*. Instead, ATP levels were significantly higher in these conditions. Given that several other genes associated with persistence were also upregulated during *hok* overexpression [37–40, 63], it is possible that *hok* affects antibiotic tolerance in *E. amylovora* through induction of stress response genes that help cells to mitigate antibiotic stressors. While the importance of type II TA systems in antibiotic survival is still being debated, this study adds to the body of evidence that Type I systems may play such a role.

To our knowledge, this is the first study of the transcriptomic response to a type I membrane-disrupting toxin. Overall, the response of *E. amylovora* to *hok* expression appears highly congruent with bacterial responses to other PMF-dissipating stresses. Alkaline conditions, in which the PMF is dissipated to maintain the inverted pH gradient, results in upregulation of ATP synthases and downregulation of chemotaxis genes in *E. coli* [64]. The membrane-targeting antibiotic polymyxin strongly upregulates metabolic pathways while repressing key virulence factor genes in *Staphylococcus aureus* [22]. Exposure to the membrane destabilizer 1-(1-naphthylmethyl)-piperazine causes upregulation of many stress response genes, including *dnaJ*, *dnaK*, and *pspABCD* [26]. Although it is still unclear whether Hok exhibits any direct regulatory roles, the overlapping downstream transcriptional effects due to *hok* induction and other membrane perturbing agents suggest that formation of membrane pore is at least one of the main reasons for the transcriptomic changes observed during *hok* induction.

**Fig. 8:**
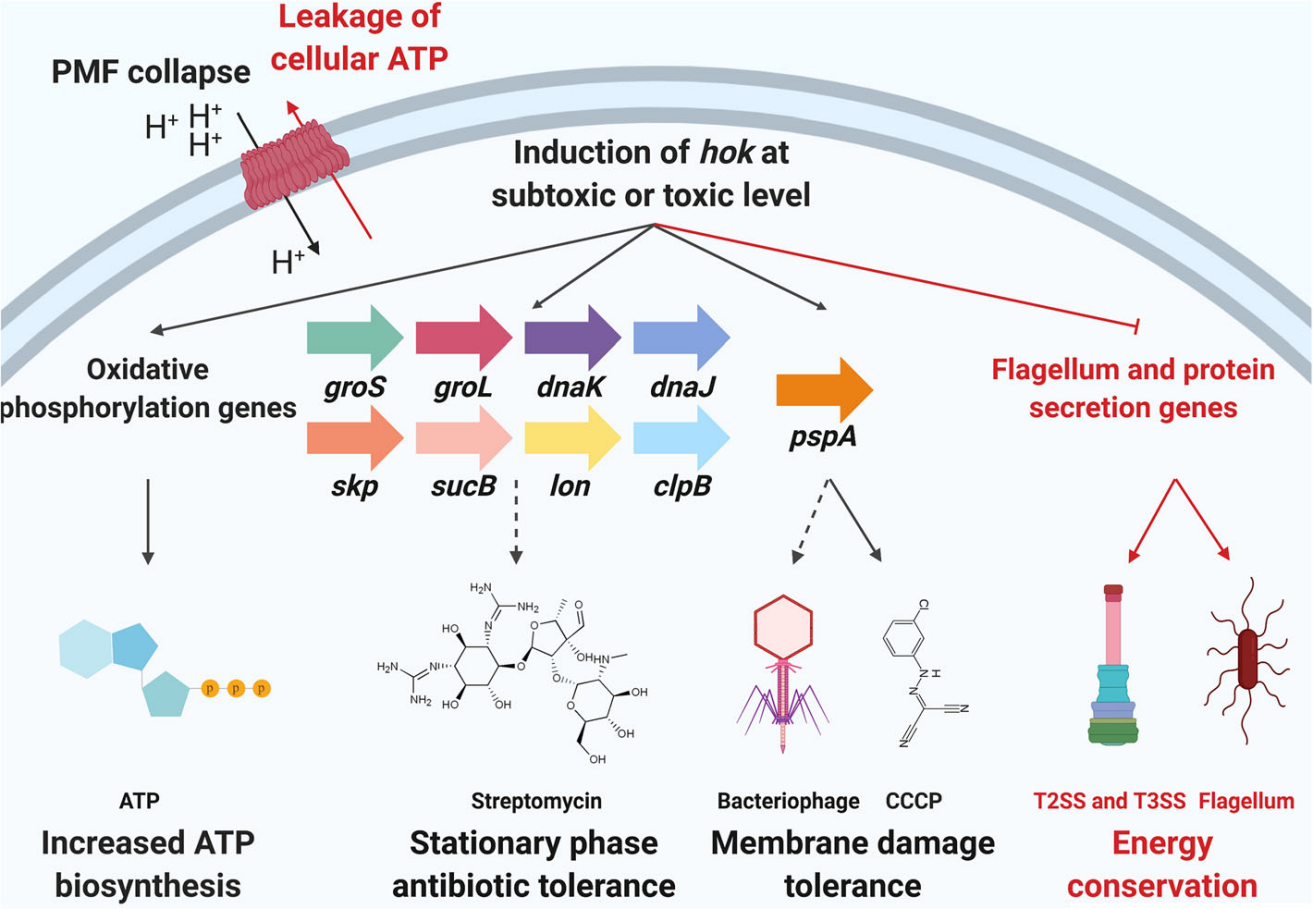
Working model of the effects of *hok* induction at subtoxic or toxic level. Induction of *hok* at a subtoxic level causes dissipation of the PMF, increased ATP biosynthesis, increased tolerance to antibiotics and further membrane stress (black arrows and letters). Induction of *hok* at a toxic level leads to leakage of cellular ATP and inactivation of energy-consuming cell machineries including T2SS, T3SS, and flagellum (red arrows and letters)

A working model summarizing the effects of *hok* induced at subtoxic or toxic levels in *E. amylovora* is shown in Fig. 8. The shared phenotypes of membrane-associated type I TA systems that have been investigated in this study and previous studies are compared (Table S3).

## Conclusions

By examining the physiological and transcriptomic changes of *Erwinia amylovora* cells expressing the type I toxin/antitoxin system toxin gene *hok* at subtoxic or toxic levels, we demonstrated that low-level expression of *hok*, while not affecting bacterial culturability, triggers expression of the ATP synthase genes and overproduction of cellular ATP. Low-level expression of *hok* also activates multiple genes associated with stress response, and triggers expression of the phage shock protein gene *pspA*, which then functions to protect *E. amylovora* cells from further membrane stressors. This study contributes to the idea that stress management is an important selective advantage of TA systems when these systems are under low level expression conditions. Although the *E. coli* Hok has been implied as a target for killing host bacterial cells [13, 14], the significant transcriptomic and physiological changes during *E. amylovora hok* overexpression presented in this study suggest that additional considerations are requisite in applying this toxin to fire blight disease management.

## Methods

### Bacterial strains, plasmids, and growth conditions

The bacterial strains and plasmids used in this study are listed in Table 1. *E. amylovora* strains were routinely grown at 28 °C in Luria-Bertani (LB) agar or broth. Unless stated otherwise, the following antibiotics were supplemented in the medium at the concentrations indicated: ampicillin (Ap; 100 μg/ml), chloramphenicol (Cm; 15 μg/ml), or kanamycin (Km; 25 μg/ml). For tolerance screening, streptomycin was applied at 100 μg/ml.

**Table 1:** Bacteria strains or plasmids used in this study and their relevant characteristics

Strain or plasmid		Genotype	Reference
Strains	Ea1189	Wild type	[65]
	Ea1189Δ*atpB*	*atpB* deletion mutant	This study
	Ea1189Δ*atpBC*	Deletion of the chromosomal region that spans the *atpB*, *atpE*, *atpF*, *atpH*, *atpA*, *atpG*, *atpD*, and *atpC* genes	This study
Plasmids	pKD3	Contains FRT-flanked Cm^r^ cassette sites; R6K *ori*; Cm^r^	[66]
	pKD46	Contains lambda Red recombinase induced by L-Arabinose; R101 *ori*; Ap^r^	[66]
	pEVS143	Broad-host-range cloning vector; IPTG inducible Cm^r^; pES213 *ori*; Km^r^	[67]
	pOE-*hok*	pEVS143 cmR::*hok*; overexpression vector; Km^r^	[27]
	pBAD33	Broad-host-range cloning vector; arabinose inducible Cm^r^; pACYC18 *ori*	[68]
	pBAD33-*pspA*	pBAD33 cmR::*pspA*; overexpression vector; Cm^r^	This study
	pPROBE-NT	Broad-host-range promoter-probe vector; pBBR1 *ori*; Km^r^	[69]
	pPROBE-*pspA*	pPROBE-NT::*pspA*; native promoter of *pspA* in pPROBE-NT; Km^r^	This study

### DNA manipulations

The *atpB* gene or the chromosomal region that spanned the entire ATP synthase gene cluster (*atpB, atpE, atpF, atpH, atpA, atpG, atpD,* and *atpC*) was deleted from *E. amylovora* Ea1189 using the λ Red recombinase system [65, 66] to generate Ea1189*ΔatpB* and Ea1189*ΔatpBC*, respectively. The pBAD33-*pspA* and pPROBE-NT:*pspA* constructs were generated by cloning the coding region of *pspA* or the 500 bp upstream the coding region into pBAD33 and pPROBE-NT, respectively, using the FastCloning approach [70]. The oligonucleotide primers used in this study are listed in Table 2. *E. coli* and *E. amylovora* cells were transformed routinely using heat shock and electroporation approaches, respectively.

**Table 2:** Oligonucleotide primers used in this study

Primer Name	Sequence (5′-3′)	Reference
**Primers used for making knockout mutants**		
atpBC_F	ATGGCTGCAGGAGAAATCTCTACGCCGCAAGAGTACATAGGTCATCATCTGTGTAGGCTGGAGCTGCTTC	This study
atpBC_R	TTACATCGCGTTTTTGGTCAACTCGATCACGCGCAGTTTGGCGATCGCTTCATATGAATATCCTCCTTA	This study
atpB_R	TCAATGTTCTTCAGATGCCATCGACAGATAGACAATCGTTAAGACCATGACATATGAATATCCTCCTTA	This study
**Primers used for the overexpression construct of** ***pspA***		
pBAD_FC_F	GGCTGTTTTGGCGGATGAGA	This study
pBAD_FC_R	AATTCGCTAGCCCAAAAAAACGG	This study
pspA_FC_F	CCGTTTTTTTGGGCTAGCGAATTATGGGTATTTTTTCACGTTTTGCCG	This study
pspA_FC_R	TCTCATCCGCCAAAACAGCCTTATTCACCGATACGCCGGTT	This study
**Primers used for confirming knockout of genes**		
atpBC_CF	CCGGCTGTAATTAACAACAAAG	This study
atpBC_CR	TTTCCTGACTGGCCTTCT	This study
atpB_CR	CCATGTACAGCAGATCCATATT	This study
**Primers used for the transcriptional fusion construct of** ***pspA***		
pspA_tsc_F	CGACCTGAATGGAAGCCGGCGCCAGTTCGTCGAGAAACAAC	This study
pspA_tsc_R	GAGCTCGGTACCCGGGGATCCTCAATCAAATTCCTCATCAGTCTGG	This study
pPROBE-NT_tsc_F	GAGGATCCCCGGGTACCGAGCTC	[27]
pPROBE-NT_tsc_R	GCCGGCTTCCATTCAGGTCG	[27]
**Primers used for qRT-PCR**		
qhok_F	TGGTGCGTACTTATAGTGTGTG	This study
qhok_R	CCGGATTCGTAAGCCATGAA	This study
qpspA_F	GACCTGATTGCTGCTTTGC	This study
qpspA_R	GGTTTCAGCCAGTTTACTTTCC	This study
qatpG_F	GTCGGCTATCTGGTCGTATCT	This study
qatpG_R	GCCTTTATCAGCCCAGGATTT	This study
qkatA_F	CGCGACTGGGTGTTAACTATAA	This study
qkatA_R	TGGATCAGAGGCAAGATCAATAC	This study
qhrpA_F	AGCACTTCAGCATCCAAGAC	This study
qhrpA_R	CGAGTTCTGCGTATCCATCTTC	This study

### Growth arrest assay

*E. amylovora* cultures grown overnight for 20 h in LB broth were washed twice and diluted to OD_600_ = 0.2 in fresh LB broth. Serially diluted IPTG (1 mM, 0.1 mM, 0.01 mM, 0.001 mM) or H_2_O was supplemented, and the cultures were incubated at 28 °C with 200 rpm shaking for 1 h. Survival rate was determined by the ratio of colony forming units (CFUs) per ml calculated through dilution plating in Ea1189(pOE-*hok*) to that in Ea1189(pEVS143). To test the effect of bacterial metabolites on the toxicity of Hok, cultures were prepared the same as the growth arrest assay, except that 10 mM mannitol or 10 mM arabinose was added to the cultures right before IPTG was added, and the OD_600_ was measured 4 h after the incubation using a spectrophotometer plate reader (Tecan; Männedorf, Switzerland).

### Measurement of membrane polarity and membrane integrity through flow cytometry

Changes in membrane polarity and membrane integrity was measured using bis-(1,3-dibutylbarbituric acid) trimethine oxonol (DiBAC_4_ [3]; Thermo Fisher Scientific; Waltham, MA) and propidium iodide (PI; Thermo Fisher Scientific), respectively, following a published protocol [71] with minor modifications. DiBAC_4_ [3] is a slow-response potential-sensitive dye that enters depolarized cells and exhibits fluorescence (excitation/emission = 490 nm/516 nm) after binding to membrane proteins. Propidium iodide exhibits fluorescence (excitation/emission, 535 nm/617 nm) after binding to DNA of membrane integrity-compromised cells. Briefly, *E. amylovora* cultures grown for 20 h were washed twice with fresh LB and adjusted to OD_600_ = 0.5. Mannitol or arabinose at 10 mM or H_2_O was supplemented followed by addition of serially diluted IPTG (1 mM, 0.1 mM, 0.01 mM, 0.001 mM) or H_2_O of the same volume for 1 h at 28 °C with shaking. Cells were washed twice with 0.5× Phosphate-Buffered Saline (PBS) to the original volume, and 250 nM DiBAC4 [3] or 20 μg/ml PI was added. The mixtures were incubated in a 28 °C incubator for 1 h without shaking. Fluorescence was measured using a BD LSR II flow cytometer (BD Biosciences; Franklin Lakes, NJ) equipped with a 488 nm laser and a 530/30 emission filter for DiBAC_4_ [3] staining and a 561 nm laser and a 620/15 emission filter for PI staining. A minimum of 10,000 events were collected for each sample. Subsequent analyses were conducted using Flowing Software 2.5.1 and R v3.4.0.

### Total RNA extraction

Cultures of Ea1189(pEVS143) and Ea1189(pOE-*hok*) grown for 20 h were washed twice and diluted to OD_600_ = 0.2 in fresh LB broth. H_2_O or 1 mM IPTG was supplemented to Ea1189(pOE-*hok*) cultures, representing induction of *hok* at subtoxic and toxic level, respectively. Ea1189(pEVS143) cultures supplemented with H_2_O of the same volume represented cells expressing wild-type level of *hok*. Each treatment had three biological replicates. Bacterial cells were harvested 1 h after the addition of IPTG or H_2_O. Total RNA extraction was modified from a previous reported method [72]. Briefly, cell pellets of *E. amylovora* cultures were resuspended in 200 μl of 0.1% N-lauroyl sarcosine sodium salt followed by centrifugation at 13,000×g for 1 min. The cell pellets were resuspended in 100 μl acetate/SDS solution (1% SDS in 10 mM EDTA and 50 mM sodium acetate, pH 5.1) and incubated for 5 min in boiling water. After centrifugation, RNA was extracted from the supernatants using the RNA Clean & Concentrator-25 kit (Zymo Research; Irvine, CA) following manufacturer’s instructions. Residual DNA contamination was removed by TURBO DNA-free Kit (Thermo Fisher Scientific) following the manufacturer’s instructions.

### Library preparation and sequencing

The quality of RNA was analyzed on a 2100 Bioanalyzer (Agilent Technologies; Santa Clara, CA). The integrity of RNA was examined via electrophoresis on a 1% agarose gel. RNA libraries were prepared by Illumina TruSeq Stranded Total RNA Library Prep Kit (bacteria) (Illumina; San Diego, CA). Sequencing was conducted with the 50 bp single-end format on the Illumina HiSeq 4000 platform at the Michigan State University Research Technology Support Facility.

### Data analysis

Adaptor sequences and low-quality reads were filtered through Trimmomatic v0.32 with following parameters: ILLUMINACLIP:$ADAPTOR:2:30:10 LEADING:3 TRAILING:3 SLIDINGWINDOW:4:30 [73]. The remaining reads were mapped to the genome of *E. amylovora* ATCC 49964 using Bowtie 2 v2.3.4.3 [74]. Counts of reads for each annotated gene were obtained using HTSeq v0.6.1 [75]. TMM-normalized CPM were called via edgeR [76]. DEGs were defined as greater than 2-fold change of CPM value and less than 0.05 of the corresponding FDR value. GO enrichment analysis was conducted on AgriGO v2.0 [77], with a cutoff FDR of 0.01. Redundant GO terms were filtered through REVIGO using the default setting [78].

### qRT-PCR

Cultures were grown in the same condition as the RNA-seq experiment and total RNA from three biological replicates was extracted and pooled together. cDNA was synthesized from 1000 ng total RNA using the High Capacity cDNA Reverse Transcription kit (Thermo Fisher Scientific) following manufacturer’s instructions. Gene expression levels were quantified via qRT-PCR using a StepOne Plus Real-Time PCR system (Applied Biosystems, Foster City, CA, USA) under the routine condition [79] with three replications. The housekeeping gene *recA* was used as an endogenous reference gene [79]. Fold changes of gene expression were quantified via the 2^-ΔΔC^_T_ method.

### Quantification of intracellular and extracellular ATP

BacTiter-Glo™ reagent (Promega; Madison, WI) was prepared following the manufacturer’s instructions. The BacTiter-Glo™ reagent supports bacterial lysis, ATP extraction, and ATP quantification based on the luminescent signal from a thermostable luciferase. *E. amylovora* cultures were grown and treated in the same condition as that of the growth arrest assay in this study. To measure intracellular ATP levels, cultures were washed twice and resuspended in 0.5× PBS of the same volume to remove any extracellular ATP. One hundred μl of the culture suspension was mixed with equal volume of the BacTiter-Glo™ reagent prepared in a 96-well plate. The mixtures were incubated at room temperature for 5 min before luminescence was measured. Extracellular ATP levels were measured following the same procedure for intracellular ATP measurement, except that supernatants of the cultures from the first centrifugation were used for quantification. Total ATP was defined as the sum up of the intracellular and the extracellular levels of ATP.

### Promoter activity of *pspABCD* operon and CCCP tolerance assay

Promoter activity of *pspABCD* was monitored in *E. amylovora* Ea1189 cultures containing the transcriptional fusion construct pPROBE-NT:*pspA*. The overnight cultures of Ea1189(pPROBE-NT:*pspA*) were washed and resuspended in LB broth to a final OD_600_ of 1.0. The cultures were incubated with approximately 10^8^ plaque forming units of bacteriophage ΦEa31–3 [80], 40 μM CCCP, 5% ethanol, or 0.1% Triton X-100 for 5 h. The Gfp fluorescence was measured on a spectrophotometer plate reader (Tecan). To measure tolerance of *E. amylovora* cultures during membrane stress, 200 μM CCCP was supplemented to the washed *E. amylovora* cultures at OD_600_ = 1.0 followed by incubation for 5 h. Survival rate was determined as the ratio of CFU/ml after the treatment to that before the treatment.

### Tolerance of stationary phase cultures to streptomycin

Single colonies of *E. amylovora* cultures were in grown in LB broth amended with selective antibiotics for 20 h to reach stationary phase. Cultures were washed twice by fresh LB broth before subjecting to 100 μg/ml streptomycin. After incubation at 28 °C with shaking for 5 h, cultures were washed once and serially diluted in 0.5× PBS buffer, and plated in LB plates without any antibiotics. Survival rate was determined as the ratio of CFU/ml after the treatment to that before the treatment.

## Supplementary Information

**Additional file 1.**

## Data Availability

The reference genome sequence of *E. amylovora* was obtained from GenBank with the accession number of FN666575. The datasets generated and analyzed during the current study are available in the National Center for Biotechnology Information Sequence Read Archive repository (https://www.ncbi.nlm.nih.gov/sra) with the following accession number: SRR10489885-SRR10489893. The *E. amylovora* strains and mutants analyzed in this study are available from the corresponding author upon reasonable request.

## Abbreviations

TA: Toxin-antitoxin
PMF: Proton motive force
PNA: Peptide nucleic acid
*psp*: Phage shock protein
IPTG: Isopropyl β-D-1-thiogalactopyranoside
qRT-PCR: Quantitative real-time PCR
bis-(1,3-dibutylbarbituric acid) trimethine oxonol: DiBAC_4_ (3)
CCCP: Carbonyl cyanide-m-chlorophenylhydrazone
CPM: Counts per million reads
DEGs: Differentially-expressed genes
FDR: False discovery rate
T2SS: Type II secretion system
T3SS: Type III secretion system
*gfp*: Green fluorescence protein
LB: Luria-Bertani
PI: Propidium iodide
PBS: Phosphate-Buffered Saline
GO: Gene Ontology
CFU: Colony forming unit
Ap: Ampicillin
Cm: Chloramphenicol
Km: Kanamycin
