# Supplementary material for: Activation of metabolic and stress responses during subtoxic expression of the type I toxin hok in Erwinia amylovora
Source: BMC Genomics. 2021 Jan 22;22:74. doi: 10.1186/s12864-021-07376-w (PMC7821729; doi:10.1186/s12864-021-07376-w)
Supplement: Supplementary file 1 — Additional file 1. [file 12864_2021_7376_MOESM1_ESM.pdf]

Supplementary material

Activation of metabolic and stress responses during subtoxic expression of the type I toxin *hok*

Jingyu Peng[a], Lindsay R. Triplett[b], George W. Sundin[a]

[a] Department of Plant, Soil, and Microbial Sciences, Michigan State University, East Lansing, Michigan, USA

[b] Department of Plant Pathology and Ecology, The Connecticut Agricultural Experiment Station, New Haven, Connecticut, USA

Address correspondence to George W. Sundin, sundin@msu.edu

This file contains Table S1, Table S2, Table S3, Fig. S1, Fig. S2, Fig. S3, Fig. S4, Fig. S5, Fig. S6, Fig. S7, and Fig. S8.

Table S1. Differentially expressed genes (DEGs) in *E. amylovora* cells expressing *hok* at subtoxic or toxic level.

Gene ID[a]	Subtoxic condition			Toxic condition			Gene name	Function description[b]
	log_2 FC	P-value	FDR	log_2 FC	P-value	FDR		
EAM_0012	-0.01	9.88E-01	9.96E-01	**-1.22**	**1.76E-02**	**3.73E-02**	*budA*	acetolactate decarboxylase
EAM_0014	-0.28	6.06E-01	8.38E-01	**-1.29**	**1.87E-02**	**3.87E-02**	-	hypothetical protein
EAM_0018	0.74	3.21E-05	6.25E-04	**1.13**	**1.97E-10**	**4.77E-09**	*rbsB*	D-ribose-binding periplasmic protein
EAM_0022	-0.34	3.74E-01	6.70E-01	**-1.34**	**6.83E-04**	**2.81E-03**	*mobA*	molybdopterin-guanine dinucleotide biosynthesis protein MobA
EAM_0023	-0.39	2.25E-01	5.00E-01	**-1.13**	**4.44E-04**	**1.96E-03**	-	pseudo
EAM_0025	0.50	4.63E-02	1.70E-01	**1.07**	**1.98E-05**	**1.32E-04**	*dsbA*	thiol:disulfide interchange protein
EAM_0030	**1.28**	**1.36E-04**	**1.90E-03**	**1.23**	**2.42E-04**	**1.17E-03**	*glnG*	nitrogen regulation protein NR(I)
EAM_0031	**1.33**	**2.79E-04**	**3.34E-03**	**1.07**	**3.38E-03**	**1.03E-02**	*glnL*	nitrogen regulation two-component sensor kinase
EAM_0032	**3.63**	**1.62E-16**	**2.89E-13**	**2.94**	**5.31E-12**	**2.07E-10**	*glnA*	glutamine synthetase
EAM_0033	**1.29**	**1.80E-03**	**1.48E-02**	**2.02**	**1.79E-06**	**1.72E-05**	*typA*	tyrosine phosphorylated protein A
EAM_0042	0.87	4.65E-03	2.99E-02	**1.27**	**4.16E-05**	**2.54E-04**	*trmH*	tRNA (guanosine-2'-O)-methyltransferase
EAM_0043	0.62	2.25E-03	1.76E-02	**1.43**	**2.59E-12**	**1.09E-10**	*spoT*	guanosine-3',5'-bis(diphosphate) 3'-pyrophosphohydrolase
EAM_0044	0.33	4.73E-01	7.51E-01	**1.11**	**1.59E-02**	**3.46E-02**	*rpoZ*	DNA-directed RNA polymerase subunit omega
EAM_0046	0.04	9.45E-01	9.82E-01	**-1.44**	**1.41E-02**	**3.18E-02**	-	DNA ligase
EAM_0050	**1.45**	**4.12E-03**	**2.75E-02**	**1.82**	**3.48E-04**	**1.59E-03**	*phnA*	phosphonoacetate hydrolase
EAM_0067	0.61	2.01E-02	9.36E-02	**1.17**	**1.13E-05**	**8.30E-05**	*rph*	ribonuclease PH
EAM_0068	**1.12**	**2.80E-04**	**3.34E-03**	**1.35**	**1.23E-05**	**8.83E-05**	*pyrE*	orotate phosphoribosyltransferase
EAM_0073	**1.14**	**4.17E-03**	**2.77E-02**	**2.92**	**3.37E-12**	**1.37E-10**	*rpmB*	50S ribosomal protein L28
EAM_0074	**1.07**	**3.23E-05**	**6.26E-04**	**1.86**	**1.52E-12**	**7.09E-11**	*rpmG*	50S ribosomal protein L33
EAM_0085	0.68	5.96E-04	6.05E-03	**1.16**	**6.47E-09**	**1.16E-07**	*wabM*	glycosyl transferase family protein
EAM_0088	0.77	3.77E-03	2.58E-02	**1.38**	**2.51E-07**	**3.05E-06**	*waaC*	lipopolysaccharide heptosyltransferase-1
EAM_0089	0.63	1.29E-02	6.64E-02	**1.10**	**1.37E-05**	**9.76E-05**	*waaF*	ADP-heptose--LPS-heptosyltransferase II
EAM_0091	0.88	9.35E-05	1.43E-03	**1.01**	**8.34E-06**	**6.36E-05**	-	exported polysaccharide deacetylase
EAM_0094	0.67	2.54E-02	1.11E-01	**1.27**	**2.31E-05**	**1.51E-04**	*grxC*	glutaredoxin 3
EAM_0095	0.78	3.16E-04	3.71E-03	**1.47**	**1.64E-11**	**5.15E-10**	*secB*	protein-export protein
EAM_0096	0.56	1.42E-02	7.11E-02	**1.25**	**4.62E-08**	**6.93E-07**	*gpsA*	glycerol-3-phosphate dehydrogenase
EAM_0111	**1.51**	**5.24E-05**	**8.95E-04**	**1.41**	**1.46E-04**	**7.76E-04**	*tpiA*	triosephosphate isomerase

EAM_0114	**-1.03**	**2.87E-04**	**3.40E-03**	**-1.56**	**6.40E-08**	**9.06E-07**	*fpr*	ferredoxin--NADP reductase
EAM_0123	0.79	7.02E-02	2.30E-01	**1.10**	**1.16E-02**	**2.75E-02**	*hslV*	ATP-dependent protease
EAM_0127	0.72	7.94E-02	2.53E-01	**1.80**	**1.82E-05**	**1.24E-04**	*rpmE*	50S ribosomal protein L31
EAM_0131	-0.46	2.78E-01	5.68E-01	**-1.05**	**1.40E-02**	**3.16E-02**	-	hypothetical protein
EAM_0139	**1.27**	**2.48E-07**	**1.18E-05**	**1.65**	**2.41E-11**	**7.26E-10**	*argG*	argininosuccinate synthase
EAM_0141	-0.29	4.19E-01	7.08E-01	**-1.12**	**1.70E-03**	**5.87E-03**	-	hybrid peroxiredoxin hyprx5
EAM_0149	0.96	1.43E-03	1.23E-02	**1.45**	**1.73E-06**	**1.67E-05**	-	hypothetical protein
EAM_0151	-0.76	1.11E-02	5.97E-02	**-1.27**	**3.10E-05**	**1.96E-04**	*ilvG*	acetolactate synthase isozyme II large subunit
EAM_0160	0.40	1.54E-01	3.98E-01	**1.08**	**1.19E-04**	**6.46E-04**	*gppA*	guanosine-3',5'-bis(diphosphate) 3'-pyrophosphohydrolase
EAM_0161	0.42	2.21E-01	4.94E-01	**1.12**	**1.36E-03**	**4.97E-03**	*rhlB*	ATP-dependent RNA helicase
EAM_0167	0.75	5.27E-03	3.31E-02	**1.34**	**8.40E-07**	**8.87E-06**	*wecC*	UDP-N-acetyl-D-mannosamine dehydrogenase
EAM_0168	0.60	1.73E-03	1.44E-02	**1.24**	**1.85E-10**	**4.57E-09**	*rffG*	dTDP-glucose 4,6-dehydratase
EAM_0173	0.83	3.61E-03	2.50E-02	**1.51**	**1.49E-07**	**1.90E-06**	*rffT*	4-alpha-L-fucosyltransferase
EAM_0176	-0.29	7.10E-01	8.82E-01	**-2.01**	**1.17E-02**	**2.76E-02**	-	hypothetical protein
EAM_0177	0.57	6.30E-02	2.14E-01	**1.23**	**7.01E-05**	**4.03E-04**	*hemY*	Porphyrin biosynthetic protein
EAM_0186	0.64	3.66E-02	1.44E-01	**1.40**	**6.74E-06**	**5.34E-05**	-	hydrolase
EAM_0210	0.74	5.24E-03	3.31E-02	**1.27**	**2.06E-06**	**1.93E-05**	*ubiD*	3-octaprenyl-4-hydroxybenzoate carboxy-lyase
EAM_0221	**1.73**	**6.34E-05**	**1.04E-03**	**2.36**	**9.41E-08**	**1.26E-06**	*tufB*	elongation factor Tu
EAM_0223	0.34	2.87E-01	5.79E-01	**1.24**	**1.39E-04**	**7.41E-04**	*nusG*	transcription antitermination protein
EAM_0224	**1.80**	**1.12E-04**	**1.63E-03**	**2.96**	**1.21E-09**	**2.56E-08**	*rplK*	50S ribosomal protein L11
EAM_0225	**1.93**	**3.49E-05**	**6.51E-04**	**2.99**	**6.76E-10**	**1.48E-08**	*rplA*	50S ribosomal protein L1
EAM_0226	**1.80**	**6.93E-04**	**6.77E-03**	**2.99**	**7.51E-08**	**1.04E-06**	*rplJ*	50S ribosomal protein L10
EAM_0227	**1.40**	**1.34E-03**	**1.17E-02**	**2.43**	**8.28E-08**	**1.14E-06**	*rplL*	50S ribosomal protein L7/L12
EAM_0228	**1.16**	**1.33E-03**	**1.16E-02**	**1.90**	**2.54E-07**	**3.07E-06**	*rpoB*	DNA-directed RNA polymerase subunit beta
EAM_0229	1.05	1.42E-02	7.14E-02	**1.86**	**2.37E-05**	**1.54E-04**	*rpoC*	DNA-directed RNA polymerase subunit beta'
EAM_0230	-0.02	9.61E-01	9.89E-01	**-1.17**	**1.53E-02**	**3.36E-02**	-	pentapeptide repeat-containing protein
EAM_0232	-0.11	8.37E-01	9.39E-01	**-1.25**	**1.90E-02**	**3.92E-02**	-	Fimbrial outer membrane usher protein
EAM_0233	-0.14	7.49E-01	8.96E-01	**-1.11**	**1.12E-02**	**2.68E-02**	-	Fimbrial chaperone protein
EAM_0234	-0.21	7.59E-01	8.99E-01	**-1.55**	**2.38E-02**	**4.67E-02**	-	fimbrial protein
EAM_0239	-0.19	6.57E-01	8.58E-01	**-1.33**	**1.79E-03**	**6.18E-03**	*thiE*	thiamine-phosphate pyrophosphorylase
EAM_0240	-0.30	1.58E-01	4.05E-01	**-1.24**	**9.40E-09**	**1.64E-07**	*thiC*	thiamine biosynthesis protein
EAM_0241	**-1.13**	**2.37E-04**	**2.93E-03**	**-1.70**	**5.27E-08**	**7.78E-07**	*rsd*	regulator of sigma D

EAM_0247	0.82	1.22E-03	1.08E-02	**1.42**	**2.61E-08**	**4.06E-07**	-	lipoprotein
EAM_0251	-0.19	7.64E-01	9.03E-01	**-1.50**	**1.73E-02**	**3.68E-02**	-	hypothetical protein
EAM_0253	0.95	2.87E-06	8.93E-05	**1.61**	**6.07E-15**	**5.30E-13**	*lysC*	lysine-sensitive aspartokinase III
EAM_0266	**-2.29**	**1.62E-08**	**1.21E-06**	**-2.63**	**1.50E-10**	**3.73E-09**	-	hypothetical protein
EAM_0277	0.95	7.38E-04	7.13E-03	**1.17**	**3.12E-05**	**1.97E-04**	-	membrane protein
EAM_0282	-0.41	5.50E-01	8.03E-01	**-1.79**	**1.20E-02**	**2.82E-02**	-	phage holin
EAM_0294	0.39	4.07E-01	7.00E-01	**2.65**	**5.59E-08**	**8.13E-07**	-	hypothetical protein
EAM_0301	-0.25	6.06E-01	8.38E-01	**-1.15**	**1.76E-02**	**3.71E-02**	-	invasin
EAM_0304	-0.25	7.21E-01	8.87E-01	**-1.69**	**1.30E-02**	**2.99E-02**	-	hypothetical protein
EAM_0305	-0.74	1.84E-01	4.45E-01	**-1.41**	**1.11E-02**	**2.66E-02**	-	hypothetical protein
EAM_0316	**-2.16**	**1.57E-10**	**2.25E-08**	**-2.64**	**1.76E-14**	**1.29E-12**	*soxS*	AraC family transcriptional regulator
EAM_0320	0.54	4.87E-03	3.10E-02	**1.41**	**4.18E-13**	**2.17E-11**	-	Na(+)/H(+) exchanger
EAM_0327	0.06	8.87E-01	9.59E-01	**1.30**	**1.03E-03**	**3.97E-03**	*acs*	acetyl-coenzyme A synthetase
EAM_0328	0.38	1.34E-01	3.66E-01	**2.06**	**2.08E-15**	**2.08E-13**	*gltP*	proton glutamate symport protein
EAM_0331	-0.18	6.97E-01	8.74E-01	**-1.27**	**6.81E-03**	**1.82E-02**	-	hypothetical protein
EAM_0335	-0.11	8.25E-01	9.34E-01	**-1.17**	**1.86E-02**	**3.86E-02**	*EdcA*	signal transduction protein
EAM_0336	0.96	6.01E-04	6.05E-03	**1.11**	**7.87E-05**	**4.45E-04**	-	gluconate 2-dehydrogenase
EAM_0337	**1.41**	**4.36E-07**	**1.78E-05**	**1.20**	**1.79E-05**	**1.22E-04**	-	gluconate 2-dehydrogenase
EAM_0338	**1.59**	**9.93E-06**	**2.44E-04**	**1.45**	**4.87E-05**	**2.93E-04**	-	gluconate 2-dehydrogenase
EAM_0343	-0.13	5.48E-01	8.02E-01	**-1.42**	**3.18E-10**	**7.45E-09**	-	NAD dependent epimerase/dehydratase
EAM_0346	-0.08	8.73E-01	9.54E-01	**-1.24**	**1.34E-02**	**3.06E-02**	-	hypothetical protein
EAM_0347	**1.22**	**2.49E-03**	**1.92E-02**	**2.32**	**2.52E-08**	**3.97E-07**	-	amino acid efflux protein
EAM_0348	-0.22	6.69E-01	8.64E-01	**-1.19**	**1.73E-02**	**3.67E-02**	-	hypothetical protein
EAM_0365	-0.14	8.15E-01	9.28E-01	**-1.48**	**1.79E-02**	**3.77E-02**	-	hypothetical protein
EAM_0368	-0.18	6.93E-01	8.72E-01	**-1.09**	**1.92E-02**	**3.96E-02**	-	hypothetical protein
EAM_0369	-0.02	9.69E-01	9.90E-01	**-1.25**	**1.58E-02**	**3.44E-02**	-	hypothetical protein
EAM_0370	-0.34	6.23E-01	8.45E-01	**-1.92**	**6.98E-03**	**1.86E-02**	-	membrane protein
EAM_0371	-0.03	9.66E-01	9.90E-01	**-1.45**	**1.77E-02**	**3.73E-02**	-	outer membrane protein
EAM_0373	-0.06	8.98E-01	9.62E-01	**-1.14**	**1.23E-02**	**2.88E-02**	*clpB*	ATPase/chaperone
EAM_0375	-0.07	8.62E-01	9.50E-01	**-1.15**	**6.96E-03**	**1.85E-02**	-	hypothetical protein
EAM_0378	-0.26	6.50E-01	8.57E-01	**-1.58**	**8.06E-03**	**2.08E-02**	-	membrane protein
EAM_0379	-0.10	8.34E-01	9.37E-01	**-1.15**	**1.39E-02**	**3.14E-02**	-	membrane protein
EAM_0380	-0.13	7.91E-01	9.18E-01	**-1.18**	**1.97E-02**	**4.03E-02**	-	hypothetical protein

EAM_0384	-0.11	8.20E-01	9.32E-01	**-1.22**	**1.59E-02**	**3.46E-02**	-	hypothetical protein
EAM_0395	-0.12	8.61E-01	9.50E-01	**-1.67**	**2.06E-02**	**4.17E-02**	-	hypothetical protein
EAM_0396	0.18	7.81E-01	9.14E-01	**-1.63**	**1.65E-02**	**3.55E-02**	-	hypothetical protein
EAM_0400	-0.10	7.84E-01	9.15E-01	**-1.08**	**2.62E-03**	**8.39E-03**	-	pseudo
EAM_0407	0.87	2.33E-04	2.90E-03	**1.10**	**3.96E-06**	**3.34E-05**	-	phosphoribosylamine--glycine ligase
EAM_0408	0.97	1.48E-06	4.94E-05	**1.32**	**7.26E-11**	**2.00E-09**	-	pantoate--beta-alanine ligase
EAM_0409	0.95	8.97E-06	2.25E-04	**1.21**	**1.43E-08**	**2.36E-07**	-	asparagine synthetase
EAM_0410	**1.21**	**1.47E-03**	**1.26E-02**	**1.01**	**7.90E-03**	**2.05E-02**	-	hypothetical protein
EAM_0418	**2.32**	**3.74E-07**	**1.60E-05**	**1.68**	**1.70E-04**	**8.83E-04**	*groS*	molecular chaperone GroES
EAM_0419	**2.36**	**1.33E-09**	**1.40E-07**	**1.81**	**2.11E-06**	**1.96E-05**	*groL*	molecular chaperone GroEL
EAM_0422	0.56	3.21E-02	1.31E-01	**1.48**	**3.19E-08**	**4.92E-07**	*efp*	elongation factor P
EAM_0425	-0.96	2.55E-04	3.12E-03	**-1.34**	**4.21E-07**	**4.76E-06**	*blc*	outer membrane lipoprotein
EAM_0438	0.83	8.25E-03	4.74E-02	**1.47**	**3.44E-06**	**2.97E-05**	*hflK*	protein hflk
EAM_0439	0.82	1.26E-03	1.11E-02	**1.01**	**7.74E-05**	**4.39E-04**	*hflC*	protein HflC
EAM_0445	**-2.59**	**1.06E-13**	**3.81E-11**	**-3.64**	**2.39E-23**	**4.29E-20**	-	lipoprotein
EAM_0448	**3.31**	**6.98E-10**	**8.06E-08**	**4.03**	**3.74E-13**	**1.97E-11**	*rpsF*	30S ribosomal protein S6
EAM_0449	**3.33**	**3.52E-10**	**4.80E-08**	**3.76**	**3.58E-12**	**1.44E-10**	*priB*	primosomal replication protein N
EAM_0450	**3.31**	**6.31E-13**	**1.62E-10**	**3.91**	**1.45E-16**	**2.25E-14**	*rpsR*	30S ribosomal protein S18
EAM_0451	**2.95**	**7.24E-08**	**4.16E-06**	**3.52**	**3.56E-10**	**8.29E-09**	*rplI*	50S ribosomal protein L9
EAM_0453	0.75	1.72E-02	8.30E-02	**1.88**	**5.94E-09**	**1.07E-07**	*fklB*	FKBP-type peptidylprolyl isomerase
EAM_0455	-0.43	1.38E-01	3.76E-01	**-1.14**	**9.17E-05**	**5.10E-04**	*cysQ*	cysQ protein
EAM_0459	0.82	2.06E-04	2.63E-03	**1.25**	**1.76E-08**	**2.85E-07**	-	hypothetical protein
EAM_0462	0.29	3.55E-01	6.54E-01	**1.06**	**9.99E-04**	**3.87E-03**	*ppa*	inorganic pyrophosphatase
EAM_0463	-0.28	4.30E-01	7.18E-01	**-1.08**	**2.78E-03**	**8.81E-03**	-	methyl-accepting chemotaxis protein
EAM_0464	0.83	5.09E-04	5.43E-03	**1.36**	**1.58E-08**	**2.58E-07**	*fbp*	fructose-1,6-bisphosphatase
EAM_0469	-0.11	8.31E-01	9.35E-01	**-1.36**	**1.10E-02**	**2.63E-02**	-	hypothetical protein
EAM_0472	-0.31	5.55E-01	8.05E-01	**-1.27**	**1.68E-02**	**3.61E-02**	-	membrane protein
EAM_0477	-0.22	6.27E-01	8.48E-01	**-1.17**	**9.78E-03**	**2.42E-02**	-	transcriptional regulator
EAM_0478	0.98	4.05E-04	4.51E-03	**1.18**	**2.56E-05**	**1.65E-04**	-	phospholipid-binding lipoprotein
EAM_0481	0.54	2.45E-02	1.09E-01	**1.19**	**6.92E-07**	**7.40E-06**	-	lipoprotein
EAM_0486	-0.20	4.69E-01	7.48E-01	**-1.28**	**3.65E-06**	**3.13E-05**	-	hypothetical protein
EAM_0489	-0.65	1.48E-01	3.90E-01	**-1.46**	**1.30E-03**	**4.79E-03**	-	hypothetical protein
EAM_0497	-0.97	3.51E-04	4.03E-03	**-1.31**	**1.64E-06**	**1.60E-05**	-	hypothetical protein

EAM_0498	0.71	3.49E-03	2.45E-02	**1.84**	**1.24E-13**	**7.40E-12**	-	transporter
EAM_0499	0.64	4.31E-02	1.61E-01	**1.36**	**1.94E-05**	**1.30E-04**	*alx*	protein Alx (high pH-induced membrane-bound redox modulator)
EAM_0509	**-1.57**	**1.01E-03**	**9.18E-03**	**-1.42**	**2.90E-03**	**9.14E-03**	-	hypothetical protein
EAM_0512A	0.17	6.33E-01	8.50E-01	**1.02**	**2.94E-03**	**9.24E-03**	*pqqA*	coenzyme PQQ synthesis protein A
EAM_0513	0.71	8.37E-03	4.80E-02	**1.28**	**2.34E-06**	**2.13E-05**	*pqqB*	coenzyme PQQ synthesis protein B
EAM_0514	0.81	1.41E-03	1.21E-02	**1.05**	**3.57E-05**	**2.21E-04**	*pqqC*	coenzyme PQQ synthesis protein C
EAM_0520	-0.11	8.92E-01	9.61E-01	**-1.82**	**1.41E-02**	**3.18E-02**	-	cytochrome oxidase subunit (partial)
EAM_0529	-0.28	6.70E-01	8.64E-01	**-1.57**	**2.05E-02**	**4.15E-02**	-	hypothetical protein
EAM_0531	-0.15	6.90E-01	8.72E-01	**-1.05**	**5.99E-03**	**1.64E-02**	*rlsC*	levan regulatory protein
EAM_0532	-0.27	6.69E-01	8.64E-01	**-1.66**	**9.51E-03**	**2.37E-02**	-	hypothetical protein
EAM_0538	-0.01	1.00E+00	1.00E+00	**-1.28**	**1.29E-02**	**2.98E-02**	-	exported endonuclease
EAM_0544	-0.26	6.65E-01	8.63E-01	**-1.57**	**1.13E-02**	**2.70E-02**	-	hypothetical protein
EAM_0547	-0.46	4.60E-01	7.40E-01	**-1.67**	**8.65E-03**	**2.20E-02**	-	hypothetical protein
EAM_0560	0.07	7.82E-01	9.14E-01	**1.09**	**6.13E-05**	**3.58E-04**	-	membrane protein
EAM_0561	0.18	5.48E-01	8.02E-01	**1.22**	**4.40E-05**	**2.66E-04**	-	hypothetical protein
EAM_0565	-0.27	6.84E-01	8.69E-01	**-1.67**	**1.32E-02**	**3.02E-02**	-	membrane protein
EAM_0566	0.74	3.21E-02	1.31E-01	**1.31**	**1.74E-04**	**8.98E-04**	*rsmC*	ribosomal RNA small subunit methyltransferase C
EAM_0571	**1.20**	**6.46E-07**	**2.36E-05**	**1.91**	**6.68E-15**	**5.62E-13**	-	lipoprotein
EAM_0572	**1.33**	**3.50E-04**	**4.03E-03**	**2.57**	**2.20E-11**	**6.67E-10**	-	lipoprotein
EAM_0573	**1.05**	**3.86E-05**	**6.92E-04**	**1.91**	**2.11E-13**	**1.16E-11**	-	hypothetical protein
EAM_0574	0.93	6.02E-04	6.05E-03	**1.53**	**1.87E-08**	**2.97E-07**	-	outer membrane protein
EAM_0575	0.81	1.08E-03	9.67E-03	**1.24**	**7.62E-07**	**8.13E-06**	-	membrane protein
EAM_0576	0.94	8.26E-04	7.73E-03	**1.22**	**1.50E-05**	**1.05E-04**	-	hypothetical protein
EAM_0578	**1.52**	**1.14E-05**	**2.70E-04**	**1.48**	**1.95E-05**	**1.30E-04**	-	hypothetical protein
EAM_0579	**1.45**	**3.32E-05**	**6.35E-04**	**1.42**	**4.55E-05**	**2.75E-04**	-	hypothetical protein
EAM_0580	**2.07**	**9.80E-11**	**1.60E-08**	**1.86**	**4.35E-09**	**8.11E-08**	-	hypothetical protein
EAM_0585	**1.14**	**3.00E-06**	**9.20E-05**	**1.13**	**3.12E-06**	**2.74E-05**	-	hypothetical protein
EAM_0589	**1.38**	**6.85E-05**	**1.10E-03**	**1.29**	**1.87E-04**	**9.49E-04**	-	hypothetical protein
EAM_0595	-0.19	7.19E-01	8.87E-01	**-1.24**	**1.80E-02**	**3.77E-02**	-	hypothetical protein
EAM_0605	-0.44	1.36E-01	3.72E-01	**-1.27**	**2.50E-05**	**1.62E-04**	-	pseudo
EAM_0616	0.92	4.15E-03	2.77E-02	**1.26**	**9.79E-05**	**5.42E-04**	*deoA*	thymidine phosphorylase
EAM_0624	**-1.54**	**3.17E-07**	**1.44E-05**	**-1.73**	**1.02E-08**	**1.75E-07**	-	lipoprotein

EAM_0625	-0.80	1.01E-02	5.56E-02	**-1.35**	**1.76E-05**	**1.20E-04**	-	polypeptide transport protein
EAM_0627	-0.21	5.92E-01	8.31E-01	**-1.18**	**2.63E-03**	**8.42E-03**	-	LysR family transcriptional regulator
EAM_0628	0.86	2.37E-04	2.93E-03	**1.20**	**4.24E-07**	**4.78E-06**	-	ABC transporter ATP-binding protein
EAM_0631	-0.43	4.45E-01	7.30E-01	**-1.49**	**8.37E-03**	**2.14E-02**	-	amidase
EAM_0638	**1.38**	**2.90E-06**	**8.94E-05**	**2.04**	**1.12E-11**	**3.82E-10**	*rob*	right origin-binding protein
EAM_0639	0.70	1.26E-03	1.11E-02	**1.57**	**9.52E-13**	**4.74E-11**	*creA*	hypothetical protein
EAM_0646	0.65	1.54E-02	7.62E-02	**1.39**	**3.15E-07**	**3.70E-06**	*talB*	transaldolase B
EAM_0648	-0.14	7.36E-01	8.91E-01	**-1.18**	**6.41E-03**	**1.73E-02**	-	acyltransferase
EAM_0649	**1.51**	**5.26E-04**	**5.52E-03**	**1.64**	**1.84E-04**	**9.38E-04**	*dnaK*	chaperone protein
EAM_0650	**1.21**	**6.48E-04**	**6.50E-03**	**1.42**	**6.79E-05**	**3.91E-04**	*dnaJ*	chaperone protein
EAM_0653	0.35	4.97E-01	7.66E-01	**2.76**	**5.45E-07**	**5.99E-06**	*rpsT*	30S ribosomal protein S20
EAM_0654	0.33	2.05E-01	4.72E-01	**1.03**	**7.18E-05**	**4.11E-04**	*ribF*	bifunctional riboflavin kinase\FMN adenylyltransferase
EAM_0655	0.55	6.89E-02	2.27E-01	**1.30**	**2.42E-05**	**1.57E-04**	*ileS*	isoleucyl-tRNA synthetase
EAM_0657	0.37	2.27E-01	5.02E-01	**1.28**	**3.94E-05**	**2.42E-04**	*fkpB*	FkbB-type 16 kD peptidyl-prolyl cis-trans isomerase
EAM_0660	**1.33**	**1.44E-07**	**7.48E-06**	**1.76**	**6.20E-12**	**2.37E-10**	*carA*	carbamoyl-phosphate synthase small subunit
EAM_0661	**1.32**	**2.52E-05**	**5.21E-04**	**1.79**	**1.87E-08**	**2.97E-07**	*carB*	carbamoyl-phosphate synthase large subunit
EAM_0664	0.68	5.20E-03	3.29E-02	**1.09**	**8.23E-06**	**6.29E-05**	*apaH*	bis(5'-nucleosyl)-tetraphosphatase
EAM_0666	0.81	3.46E-03	2.44E-02	**1.33**	**1.91E-06**	**1.80E-05**	*ksgA*	dimethyladenosine transferase
EAM_0667	0.41	1.44E-01	3.85E-01	**1.46**	**3.40E-07**	**3.95E-06**	*pdxA*	4-hydroxythreonine-4-phosphate dehydrogenase
EAM_0668	0.87	1.75E-03	1.44E-02	**1.30**	**3.01E-06**	**2.65E-05**	*surA*	survival protein A
EAM_0669	0.54	8.52E-02	2.67E-01	**1.08**	**6.90E-04**	**2.84E-03**	*imp*	organic solvent tolerance protein
EAM_0673	-0.54	1.10E-02	5.92E-02	**-1.16**	**6.68E-08**	**9.39E-07**	*polB*	DNA polymerase II
EAM_0684	-0.76	1.47E-01	3.89E-01	**-1.16**	**2.40E-02**	**4.69E-02**	*leuL*	leu operon leader peptide
EAM_0690	0.54	5.00E-02	1.79E-01	**1.20**	**1.38E-05**	**9.76E-05**	*mraW*	S-adenosyl-methyltransferase
EAM_0692	0.78	3.84E-03	2.62E-02	**1.23**	**6.49E-06**	**5.17E-05**	*ftsI*	peptidoglycan synthetase
EAM_0693	0.88	2.59E-03	1.98E-02	**1.59**	**6.84E-08**	**9.57E-07**	*murE*	UDP-N-acetylmuramoylalanyl-D-glutamate--2,6-diaminopimelate ligase
EAM_0694	**1.12**	**1.09E-05**	**2.64E-04**	**1.47**	**9.46E-09**	**1.64E-07**	*murF*	UDP-N-acetylmuramoyl-tripeptide--D-alanyl-D-alanine ligase
EAM_0695	0.72	8.40E-03	4.80E-02	**1.48**	**9.51E-08**	**1.27E-06**	*mraY*	Phospho-N-acetylmuramoyl-pentapeptide-transferase
EAM_0696	0.97	9.31E-04	8.58E-03	**1.51**	**3.48E-07**	**4.02E-06**	*murD*	UDP-N-acetylmuramoylalanine--D-glutamate ligase

EAM_0698	0.91	2.09E-03	1.67E-02	**1.27**	**1.84E-05**	**1.24E-04**	*murG*	UDP-N-acetylglucosamine-N-acetylmuramyl-(pentapeptide) pyrophosphoryl-undecaprenol N-acetylglucosamine transferase
EAM_0699	**1.14**	**3.68E-04**	**4.18E-03**	**1.67**	**2.82E-07**	**3.35E-06**	*murC*	UDP-N-acetylmuramate--L-alanine ligase
EAM_0706	0.12	7.30E-01	8.89E-01	**1.05**	**2.26E-03**	**7.52E-03**	*secM*	secretion monitor
EAM_0707	0.79	2.77E-03	2.07E-02	**1.32**	**8.22E-07**	**8.72E-06**	*secA*	preprotein translocase subunit SecA
EAM_0708	-0.20	6.82E-01	8.68E-01	**-1.49**	**2.56E-03**	**8.21E-03**	-	hypothetical protein
EAM_0714	-0.26	6.42E-01	8.53E-01	**-1.31**	**2.02E-02**	**4.11E-02**	-	type II secretion system protein
EAM_0715	-0.33	6.01E-01	8.37E-01	**-1.64**	**1.19E-02**	**2.80E-02**	-	type II secretion system protein
EAM_0716	-0.48	4.27E-01	7.16E-01	**-1.46**	**1.70E-02**	**3.64E-02**	-	type II secretion system protein
EAM_0720	-0.15	7.75E-01	9.10E-01	**-1.27**	**1.68E-02**	**3.61E-02**	-	type II secretion system protein
EAM_0721	-0.12	7.97E-01	9.20E-01	**-1.05**	**2.07E-02**	**4.17E-02**	-	type II secretion system protein
EAM_0722	-0.27	5.74E-01	8.16E-01	**-1.16**	**1.53E-02**	**3.35E-02**	-	type II secretion system protein
EAM_0729	-0.28	5.71E-01	8.15E-01	**-1.25**	**1.34E-02**	**3.06E-02**	*hofC*	protein transport protein
EAM_0733	0.02	9.80E-01	9.92E-01	**-1.52**	**2.23E-02**	**4.42E-02**	-	hypothetical protein
EAM_0737	-0.34	5.32E-01	7.94E-01	**-1.52**	**7.03E-03**	**1.87E-02**	-	cytotoxic necrotizing factor
EAM_0739	**-1.25**	**3.60E-05**	**6.56E-04**	**-1.38**	**5.18E-06**	**4.19E-05**	-	glycosyl hydrolase
EAM_0741	-0.89	3.52E-02	1.40E-01	**-1.67**	**1.03E-04**	**5.70E-04**	-	glycosyl hydrolase
EAM_0743	-0.33	3.67E-01	6.62E-01	**-1.10**	**3.27E-03**	**1.01E-02**	-	glycosyl hydrolase
EAM_0744	-0.44	2.55E-01	5.39E-01	**-1.04**	**7.69E-03**	**2.00E-02**	-	major facilitator superfamily protein
EAM_0749	0.10	7.19E-01	8.87E-01	**1.55**	**8.15E-08**	**1.12E-06**	*lpdA*	dihydrolipoyl dehydrogenase (E3 component of pyruvate and 2-oxoglutarate dehydrogenases complexes)
EAM_0757	-0.48	3.57E-01	6.54E-01	**-1.48**	**5.04E-03**	**1.42E-02**	-	thiolase
EAM_0762	0.17	5.65E-01	8.12E-01	**1.05**	**3.56E-04**	**1.62E-03**	*cas3*	CRISPR-associated helicase
EAM_0763	**1.41**	**1.38E-05**	**3.18E-04**	**1.84**	**2.07E-08**	**3.29E-07**	-	CRISPR-associated protein
EAM_0764	**2.25**	**6.81E-08**	**4.16E-06**	**2.40**	**1.03E-08**	**1.75E-07**	-	CRISPR-associated protein
EAM_0765	**2.41**	**3.62E-10**	**4.80E-08**	**2.74**	**2.09E-12**	**9.26E-11**	-	CRISPR-associated protein
EAM_0766	**2.36**	**1.93E-07**	**9.88E-06**	**2.99**	**1.17E-10**	**2.96E-09**	-	CRISPR-associated protein
EAM_0767	**1.87**	**8.63E-09**	**7.19E-07**	**2.31**	**2.61E-12**	**1.09E-10**	-	CRISPR-associated protein
EAM_0768	**1.51**	**1.27E-05**	**2.94E-04**	**1.81**	**2.16E-07**	**2.70E-06**	-	CRISPR-associated protein
EAM_0769	0.65	6.33E-03	3.85E-02	**1.36**	**1.30E-08**	**2.17E-07**	-	CRISPR-associated protein
EAM_0770	-0.11	7.96E-01	9.20E-01	**-1.01**	**1.93E-02**	**3.97E-02**	-	lipoprotein
EAM_0771	-0.32	5.35E-01	7.96E-01	**-1.22**	**1.96E-02**	**4.01E-02**	-	transcriptional regulator

EAM_0772	0.79	1.03E-02	5.57E-02	**1.66**	**1.10E-07**	**1.44E-06**	*acnB*	aconitate hydratase 2
EAM_0781	-0.48	3.08E-01	6.07E-01	**-1.16**	**1.57E-02**	**3.42E-02**	-	hypothetical protein
EAM_0789	0.71	3.96E-03	2.68E-02	**1.42**	**7.48E-09**	**1.32E-07**	*panB*	3-methyl-2-oxobutanoate hydroxymethyltransferase
EAM_0790	**1.05**	**1.68E-03**	**1.41E-02**	**2.30**	**1.52E-11**	**4.85E-10**	*folK*	2-amino-4-hydroxy-6-hydroxymethyldihydropteridine pyrophosphokinase
EAM_0791	0.92	3.33E-03	2.37E-02	**2.22**	**8.28E-12**	**2.97E-10**	*pcnB*	poly(A) polymerase
EAM_0792	0.92	8.10E-03	4.68E-02	**2.34**	**7.77E-11**	**2.09E-09**	*gluQ*	glutamyl-Q tRNA(Asp) synthetase
EAM_0793	0.12	7.28E-01	8.89E-01	**1.19**	**6.10E-04**	**2.56E-03**	*dksA*	DnaK suppressor protein
EAM_0795	-0.44	3.26E-01	6.25E-01	**-1.01**	**2.47E-02**	**4.79E-02**	*ligT*	2'-5' RNA ligase
EAM_0798	-0.02	9.65E-01	9.90E-01	**1.38**	**1.67E-04**	**8.75E-04**	-	hypothetical protein
EAM_0802	-0.07	8.77E-01	9.56E-01	**-1.19**	**7.75E-03**	**2.01E-02**	*relB*	antitoxin
EAM_0803	-0.32	6.56E-01	8.57E-01	**-1.66**	**2.18E-02**	**4.35E-02**	*relE*	toxin
EAM_0804	-0.11	8.61E-01	9.50E-01	**-1.44**	**2.30E-02**	**4.54E-02**	-	hypothetical protein
EAM_0811	**-1.53**	**7.32E-08**	**4.16E-06**	**-1.81**	**2.77E-10**	**6.53E-09**	-	hypothetical protein
EAM_0819	**1.31**	**2.69E-03**	**2.02E-02**	**2.06**	**4.06E-06**	**3.41E-05**	*rpsB*	30S ribosomal protein S2
EAM_0820	**1.44**	**2.76E-04**	**3.32E-03**	**2.19**	**5.63E-08**	**8.17E-07**	*tsf*	elongation factor Ts
EAM_0822	0.52	1.24E-01	3.48E-01	**1.03**	**2.54E-03**	**8.15E-03**	*frr*	ribosome recycling factor
EAM_0823	0.38	1.35E-01	3.69E-01	**1.09**	**2.11E-05**	**1.40E-04**	*dxr*	1-deoxy-D-xylulose 5-phosphate reductoisomerase
EAM_0824	0.33	1.70E-01	4.22E-01	**1.29**	**8.33E-08**	**1.14E-06**	*uppS*	UDP pyrophosphate synthetase
EAM_0825	0.51	5.33E-02	1.87E-01	**1.36**	**2.63E-07**	**3.17E-06**	*cdsA*	phosphatidate cytidylyltransferase
EAM_0826	0.49	6.77E-02	2.24E-01	**1.24**	**5.61E-06**	**4.51E-05**	*ecfE*	protease
EAM_0827	**1.11**	**1.75E-03**	**1.44E-02**	**1.65**	**4.65E-06**	**3.83E-05**	*yaeT*	outer membrane protein assembly factor
EAM_0828	**1.64**	**1.12E-04**	**1.63E-03**	**2.13**	**7.87E-07**	**8.37E-06**	*skp*	periplasmic chaperone
EAM_0829	0.54	1.31E-01	3.61E-01	**1.35**	**1.67E-04**	**8.74E-04**	*lpxD*	UDP-3-o-[3-hydroxymyristoyl] glucosamine N-acyltransferase
EAM_0830	0.72	2.35E-02	1.06E-01	**1.71**	**1.10E-07**	**1.44E-06**	*fabZ*	(3R)-hydroxymyristoyl-ACP dehydratase
EAM_0831	0.99	4.33E-03	2.85E-02	**1.93**	**4.70E-08**	**7.02E-07**	*lpxA*	acyl-ACP--UDP-N- acetylglucosamine O-acyltransferase
EAM_0832	0.31	2.63E-01	5.48E-01	**1.26**	**9.04E-06**	**6.79E-05**	*lpxB*	lipid-A-disaccharide synthase
EAM_0834	0.66	3.00E-03	2.20E-02	**1.21**	**6.04E-08**	**8.66E-07**	*dnaE*	DNA polymerase III subunit alpha
EAM_0835	0.52	7.81E-02	2.50E-01	**1.23**	**4.33E-05**	**2.62E-04**	*accA*	acetyl-coenzyme A carboxylase carboxyl transferase subunit alpha
EAM_0843	-0.33	4.59E-01	7.40E-01	**-1.23**	**6.35E-03**	**1.72E-02**	-	membrane protein
EAM_0844	0.73	1.81E-03	1.48E-02	**1.28**	**7.31E-08**	**1.02E-06**	*proS*	prolyl-tRNA synthetase

EAM_0853	0.46	1.16E-01	3.32E-01	**1.64**	**4.33E-08**	**6.51E-07**	*mltD*	membrane-bound lytic murein transglycosylase D
EAM_0858	-0.29	5.65E-01	8.11E-01	**-1.44**	**5.61E-03**	**1.56E-02**	-	cytosine/purines uracil thiamine allantoin permease
EAM_0859	-0.32	5.19E-01	7.88E-01	**-1.27**	**1.13E-02**	**2.69E-02**	-	GntR family transcriptional regulator
EAM_0865	-0.19	6.92E-01	8.72E-01	**-1.56**	**1.64E-03**	**5.75E-03**	-	hypothetical protein
EAM_0867	-0.36	5.60E-01	8.08E-01	**-1.57**	**1.18E-02**	**2.79E-02**	-	hypothetical protein
EAM_0868	-0.37	4.16E-01	7.06E-01	**-1.05**	**2.04E-02**	**4.13E-02**	-	gamma-glutamyltranspeptidase
EAM_0872	-0.20	7.53E-01	8.96E-01	**-1.63**	**1.13E-02**	**2.69E-02**	-	ABC transporter substrate-binding protein
EAM_0873	-0.16	8.02E-01	9.23E-01	**-1.88**	**4.17E-03**	**1.22E-02**	-	ABC transporter permease
EAM_0878	-0.41	4.28E-01	7.16E-01	**-1.25**	**1.73E-02**	**3.67E-02**	-	hypothetical protein
EAM_0880	-0.48	3.66E-01	6.61E-01	**-1.52**	**5.33E-03**	**1.49E-02**	-	Xanthine/uracil permease
EAM_0891	0.54	8.64E-02	2.69E-01	**1.12**	**4.13E-04**	**1.84E-03**	*lpcA*	phosphoheptose isomerase
EAM_0894	-0.21	5.82E-01	8.23E-01	**-1.21**	**2.08E-03**	**7.04E-03**	*dinB*	DNA polymerase IV
EAM_0896	0.33	2.69E-01	5.57E-01	**1.14**	**1.87E-04**	**9.47E-04**	*gpt*	xanthine-guanine phosphoribosyltransferase
EAM_0898	0.71	2.51E-02	1.10E-01	**1.40**	**1.40E-05**	**9.87E-05**	*crl*	curlin genes transcriptional activator sigma factor-binding protein
EAM_0906	**-1.21**	**4.29E-05**	**7.57E-04**	**-1.62**	**4.92E-08**	**7.32E-07**	-	membrane protein
EAM_0907	**-1.80**	**5.81E-06**	**1.65E-04**	**-1.91**	**1.59E-06**	**1.56E-05**	-	hypothetical protein
EAM_0909	**-2.07**	**5.44E-07**	**2.07E-05**	**-2.75**	**1.06E-10**	**2.76E-09**	-	hypothetical protein
EAM_0911	**-1.11**	**1.10E-05**	**2.65E-04**	**-1.40**	**3.85E-08**	**5.87E-07**	-	hypothetical protein
EAM_0912	0.53	1.80E-02	8.62E-02	**1.07**	**2.09E-06**	**1.95E-05**	-	membrane protein
EAM_0919	0.84	3.17E-03	2.30E-02	**1.80**	**5.69E-10**	**1.27E-08**	-	ABC transporter permease
EAM_0920	**1.26**	**2.02E-05**	**4.32E-04**	**1.76**	**4.30E-09**	**8.07E-08**	-	ABC transporter substrate-binding protein
EAM_0923	**-1.41**	**2.26E-05**	**4.76E-04**	**-1.44**	**1.45E-05**	**1.02E-04**	-	hypothetical protein
EAM_0927	**-1.24**	**3.80E-04**	**4.28E-03**	**-1.76**	**6.90E-07**	**7.40E-06**	-	phosphate starvation-inducible membrane protein
EAM_0928	**-1.27**	**1.16E-05**	**2.73E-04**	**-1.32**	**4.94E-06**	**4.01E-05**	-	membrane-associated aspartyl/asparaginyl beta-hydroxylase
EAM_0933	-0.89	4.01E-02	1.53E-01	**-2.25**	**6.82E-07**	**7.34E-06**	-	hypothetical protein
EAM_0939	0.99	3.42E-06	1.02E-04	**1.19**	**2.55E-08**	**4.00E-07**	*rdgC*	recombination associated protein
EAM_0940	-0.35	5.25E-01	7.92E-01	**-1.46**	**9.76E-03**	**2.42E-02**	-	amino acid permease
EAM_0941	-0.44	3.59E-01	6.56E-01	**-1.61**	**9.61E-04**	**3.74E-03**	-	phosphotransferase/kinase
EAM_0942	-0.43	3.92E-01	6.86E-01	**-1.67**	**1.27E-03**	**4.72E-03**	-	AraC family transcriptional regulator
EAM_0944	0.43	3.84E-02	1.48E-01	**1.21**	**5.08E-09**	**9.28E-08**	-	outer membrane lipoprotein
EAM_0955	0.72	3.89E-02	1.50E-01	**1.28**	**2.85E-04**	**1.34E-03**	-	peroxidase

EAM_0957	0.20	3.64E-01	6.60E-01	**1.02**	**6.37E-06**	**5.08E-05**	*queA*	S-adenosylmethionine--tRNA ribosyltransferase-isomerase
EAM_0958	**1.18**	**6.53E-06**	**1.79E-04**	**1.73**	**7.73E-11**	**2.09E-09**	-	membrane protein
EAM_0959	0.89	2.53E-05	5.21E-04	**1.33**	**4.00E-10**	**9.19E-09**	-	bactoprenol glucosyl transferase
EAM_0960	0.32	2.53E-01	5.37E-01	**1.37**	**1.33E-06**	**1.33E-05**	*gtrA*	bactoprenol-linked glucose translocase
EAM_0961	0.23	2.37E-01	5.15E-01	**1.33**	**7.60E-12**	**2.78E-10**	*tgt*	queuine tRNA-ribosyltransferase
EAM_0962	0.27	2.42E-01	5.21E-01	**1.20**	**2.20E-07**	**2.74E-06**	-	preprotein translocase subunit
EAM_0963	0.86	2.10E-03	1.67E-02	**2.00**	**3.16E-12**	**1.30E-10**	*secD*	protein-export membrane protein
EAM_0964	0.66	1.03E-02	5.59E-02	**1.75**	**1.79E-11**	**5.58E-10**	*secF*	protein-export membrane protein
EAM_0971	0.29	3.77E-01	6.73E-01	**1.09**	**1.16E-03**	**4.37E-03**	*thiL*	thiamine-monophosphate kinase
EAM_0981	0.46	1.48E-01	3.89E-01	**1.44**	**1.08E-05**	**7.95E-05**	*cyoE*	protoheme IX farnesyltransferase
EAM_0982	0.91	6.59E-02	2.21E-01	**1.89**	**2.08E-04**	**1.02E-03**	*cyoD*	cytochrome O ubiquinol oxidase subunit IV
EAM_0983	**1.08**	**2.66E-03**	**2.01E-02**	**1.73**	**2.17E-06**	**2.00E-05**	*cyoC*	cytochrome O ubiquinol oxidase subunit III
EAM_0984	**1.23**	**2.45E-03**	**1.90E-02**	**1.85**	**7.65E-06**	**5.97E-05**	*cyoB*	cytochrome O ubiquinol oxidase subunit I
EAM_0985	**1.31**	**1.54E-04**	**2.09E-03**	**1.67**	**1.90E-06**	**1.80E-05**	*cyoA*	cytochrome O ubiquinol oxidase subunit II
EAM_0989	**1.39**	**6.86E-05**	**1.10E-03**	**2.29**	**1.86E-10**	**4.57E-09**	*tig*	trigger factor
EAM_0994	**1.39**	**5.65E-06**	**1.62E-04**	**1.11**	**2.48E-04**	**1.19E-03**	*lon*	ATP-dependent protease La
EAM_0995	0.76	2.57E-04	3.13E-03	**1.23**	**5.36E-09**	**9.75E-08**	*hupB*	DNA-binding protein HU-beta
EAM_0996	0.87	3.51E-03	2.45E-02	**1.54**	**3.13E-07**	**3.69E-06**	*ppiD*	peptidyl-prolyl cis-trans isomerase D
EAM_1004	**3.01**	**7.81E-09**	**6.66E-07**	**2.11**	**2.96E-05**	**1.88E-04**	*glnK*	nitrogen regulatory protein P-II
EAM_1007	-1.01	2.32E-02	1.05E-01	**-1.05**	**1.89E-02**	**3.90E-02**	-	lipoprotein
EAM_1013	-0.38	3.08E-01	6.07E-01	**-1.03**	**6.23E-03**	**1.69E-02**	-	hypothetical protein
EAM_1015	-0.43	3.14E-01	6.12E-01	**-1.07**	**1.28E-02**	**2.95E-02**	*rpmE2*	50S ribosomal protein L31
EAM_1016	0.50	3.38E-02	1.36E-01	**1.09**	**4.26E-06**	**3.53E-05**	*acrB*	multidrug efflux pump
EAM_1023	0.59	6.14E-03	3.76E-02	**1.83**	**9.66E-17**	**1.65E-14**	*apt*	adenine phosphoribosyltransferase
EAM_1027	**1.65**	**1.97E-04**	**2.54E-03**	**1.35**	**2.11E-03**	**7.12E-03**	*htpG*	heat shock protein HtpG
EAM_1032	-0.46	1.75E-01	4.29E-01	**-1.13**	**1.03E-03**	**3.96E-03**	-	membrane protein
EAM_1034	0.68	3.59E-02	1.42E-01	**1.43**	**1.58E-05**	**1.10E-04**	*hemH*	ferrochelatase
EAM_1040	-0.96	8.41E-03	4.80E-02	**-2.05**	**5.68E-08**	**8.20E-07**	-	membrane protein
EAM_1051	-0.48	3.97E-01	6.91E-01	**-1.44**	**1.23E-02**	**2.87E-02**	-	colicin immunity protein
EAM_1053	-0.33	5.64E-01	8.11E-01	**-1.38**	**1.87E-02**	**3.87E-02**	-	hypothetical protein
EAM_1057	**-1.25**	**1.65E-05**	**3.64E-04**	**-1.27**	**1.16E-05**	**8.46E-05**	-	ABC transporter ATP-binding protein
EAM_1058	**-1.09**	**6.93E-04**	**6.77E-03**	**-1.11**	**5.89E-04**	**2.48E-03**	-	ABC transporter permease

EAM_1060	0.77	4.34E-02	1.62E-01	**1.72**	**9.06E-06**	**6.79E-05**	*purE*	phosphoribosylaminoimidazole carboxylase catalytic subunit
EAM_1061	0.34	2.45E-01	5.26E-01	**1.17**	**6.66E-05**	**3.84E-04**	*lpxH*	UDP-2,3-diacylglucosamine hydrolase
EAM_1068	-0.77	1.94E-01	4.58E-01	**-1.51**	**1.24E-02**	**2.89E-02**	-	hypothetical protein
EAM_1069	-0.36	4.49E-01	7.30E-01	**-1.19**	**1.18E-02**	**2.78E-02**	-	hypothetical protein
EAM_1070	-0.28	5.18E-01	7.87E-01	**-1.18**	**7.00E-03**	**1.86E-02**	-	hypothetical protein
EAM_1071	-0.49	8.93E-02	2.75E-01	**-1.17**	**5.22E-05**	**3.12E-04**	-	hypothetical protein
EAM_1072	-0.21	6.40E-01	8.53E-01	**-1.39**	**2.65E-03**	**8.45E-03**	-	hypothetical protein
EAM_1086	-1.00	3.23E-04	3.77E-03	**-1.20**	**1.82E-05**	**1.24E-04**	-	lipoprotein
EAM_1103	-0.45	1.06E-01	3.11E-01	**-1.13**	**5.33E-05**	**3.16E-04**	-	hypothetical protein
EAM_1110	-0.70	2.85E-01	5.76E-01	**-1.68**	**1.21E-02**	**2.84E-02**	-	pseudo
EAM_1111	-0.55	4.39E-01	7.26E-01	**-1.64**	**2.36E-02**	**4.63E-02**	-	hypothetical protein
EAM_1113	-0.04	1.00E+00	1.00E+00	**-2.40**	**2.40E-02**	**4.69E-02**	-	hypothetical protein
EAM_1122	0.68	2.82E-02	1.20E-01	**1.36**	**1.32E-05**	**9.49E-05**	*dacA*	penicillin-binding protein 5
EAM_1127	0.82	3.43E-02	1.38E-01	**1.78**	**7.05E-06**	**5.57E-05**	-	hypothetical protein
EAM_1128	0.76	1.70E-02	8.22E-02	**1.67**	**2.40E-07**	**2.95E-06**	*nadD*	nicotinate-nucleotide adenylyltransferase
EAM_1129	0.60	2.33E-02	1.05E-01	**1.72**	**1.34E-10**	**3.37E-09**	*holA*	DNA polymerase III subunit delta
EAM_1137	0.50	4.40E-02	1.63E-01	**1.05**	**2.52E-05**	**1.63E-04**	*cutE*	apolipoprotein N-acyltransferase
EAM_1140	0.88	4.88E-04	5.23E-03	**1.29**	**4.56E-07**	**5.10E-06**	-	PhoH-like ATP-binding protein
EAM_1143	**1.02**	**1.66E-05**	**3.64E-04**	**1.05**	**8.46E-06**	**6.43E-05**	*asnB*	asparagine synthetase B
EAM_1144	0.92	8.20E-04	7.69E-03	**1.24**	**7.78E-06**	**6.04E-05**	*nagD*	ribonucleotide monophosphatase
EAM_1149	0.63	2.51E-02	1.10E-01	**1.22**	**1.57E-05**	**1.09E-04**	*glnS*	glutaminyl-tRNA synthetase
EAM_1153	0.73	6.47E-03	3.90E-02	**1.06**	**7.48E-05**	**4.26E-04**	-	hypothetical protein
EAM_1155	0.80	2.11E-03	1.67E-02	**1.15**	**9.74E-06**	**7.24E-05**	*seqA*	negative regulator of replication initiation
EAM_1156	0.79	1.70E-02	8.22E-02	**1.24**	**1.81E-04**	**9.26E-04**	*pgm*	phosphoglucomutase
EAM_1158	**-1.12**	**5.02E-03**	**3.19E-02**	**-1.85**	**5.53E-06**	**4.45E-05**	-	membrane protein
EAM_1167	0.84	7.95E-04	7.55E-03	**1.05**	**3.16E-05**	**1.99E-04**	*gltA*	citrate synthase
EAM_1169	0.02	9.70E-01	9.90E-01	**1.93**	**2.11E-05**	**1.40E-04**	*sdhD*	succinate dehydrogenase hydrophobic membrane anchor protein
EAM_1170	0.23	4.70E-01	7.49E-01	**1.76**	**6.03E-08**	**8.66E-07**	*sdhA*	succinate dehydrogenase flavoprotein subunit
EAM_1171	0.33	3.69E-01	6.64E-01	**1.40**	**1.76E-04**	**9.06E-04**	*sdhB*	succinate dehydrogenase iron-sulfur protein
EAM_1172	**1.15**	**1.70E-03**	**1.42E-02**	**1.65**	**7.92E-06**	**6.09E-05**	*sucA*	2-oxoglutarate dehydrogenase E1

EAM_1173	**1.56**	**1.25E-04**	**1.78E-03**	**1.47**	**2.86E-04**	**1.34E-03**	*sucB*	dihydrolipoyllysine-residue succinyltransferase component of 2 oxoglutarate dehydrogenase complex
EAM_1174	**1.67**	**4.74E-04**	**5.10E-03**	**1.75**	**2.69E-04**	**1.28E-03**	*sucC*	succinyl-CoA synthetase subunit beta
EAM_1175	**1.63**	**1.48E-04**	**2.03E-03**	**1.64**	**1.32E-04**	**7.07E-04**	*sucD*	succinyl-CoA ligase [ADP-forming] subunit alpha
EAM_1180	0.82	2.56E-02	1.12E-01	**1.35**	**2.91E-04**	**1.37E-03**	*tolB*	TolB colicin import protein
EAM_1181	0.98	6.28E-03	3.83E-02	**1.59**	**1.19E-05**	**8.68E-05**	*pal*	peptidoglycan-associated lipoprotein
EAM_1182	0.63	2.17E-02	9.95E-02	**1.15**	**3.52E-05**	**2.18E-04**	-	hypothetical protein
EAM_1183	-0.29	6.78E-01	8.68E-01	**-1.88**	**7.67E-03**	**2.00E-02**	-	hypothetical protein
EAM_1192	-0.32	6.23E-01	8.45E-01	**-1.58**	**1.78E-02**	**3.74E-02**	-	RHS family protein
EAM_1193	-0.25	6.05E-01	8.38E-01	**-1.18**	**1.46E-02**	**3.26E-02**	-	RHS family protein
EAM_1194	0.51	1.15E-01	3.30E-01	**1.04**	**1.44E-03**	**5.17E-03**	*gpmA*	2,3-bisphosphoglycerate-independent phosphoglycerate mutase
EAM_1206	0.45	6.26E-02	2.13E-01	**1.15**	**3.01E-06**	**2.65E-05**	*pgl*	6-phosphogluconolactonase
EAM_1209	-0.55	1.32E-01	3.64E-01	**-1.31**	**4.56E-04**	**1.99E-03**	*bioA*	adenosylmethionine-8-amino-7-oxononanoate aminotransferase
EAM_1210	-0.42	4.96E-01	7.66E-01	**-1.72**	**6.25E-03**	**1.70E-02**	*bioB*	biotin synthase
EAM_1211	-0.54	2.92E-01	5.84E-01	**-1.41**	**7.25E-03**	**1.91E-02**	*bioF*	8-amino-7-oxononanoate synthase
EAM_1212	-0.34	4.89E-01	7.60E-01	**-1.09**	**2.55E-02**	**4.91E-02**	*bioC*	biotin synthesis protein
EAM_1213	-0.32	5.66E-01	8.12E-01	**-1.46**	**9.23E-03**	**2.32E-02**	*bioD*	dethiobiotin synthetase
EAM_1214	-0.48	4.17E-01	7.07E-01	**-1.50**	**1.22E-02**	**2.85E-02**	-	ABC transporter ATP-binding protein
EAM_1228	0.47	1.35E-01	3.69E-01	**1.02**	**1.37E-03**	**4.99E-03**	-	hypothetical protein
EAM_1231	**-2.60**	**1.39E-06**	**4.69E-05**	**-2.79**	**2.73E-07**	**3.28E-06**	-	membrane protein
EAM_1232	**-1.44**	**4.52E-07**	**1.82E-05**	**-1.72**	**2.26E-09**	**4.56E-08**	*pbpG*	penicillin-binding protein 7
EAM_1249	-0.55	2.40E-01	5.19E-01	**-1.45**	**2.30E-03**	**7.61E-03**	-	acetyltransferase
EAM_1253	**-3.61**	**2.13E-15**	**1.27E-12**	**-3.55**	**5.04E-15**	**4.51E-13**	*hutU*	urocanate hydratase
EAM_1254	**-3.12**	**5.50E-11**	**9.85E-09**	**-3.11**	**6.16E-11**	**1.72E-09**	*hutH*	histidine ammonia-lyase
EAM_1256	**-1.82**	**1.36E-04**	**1.90E-03**	**-2.30**	**2.17E-06**	**2.00E-05**	-	hypothetical protein
EAM_1257	**-2.84**	**1.07E-15**	**9.55E-13**	**-3.37**	**1.46E-20**	**4.75E-18**	-	chlorohydrolase
EAM_1258	**-1.51**	**4.61E-03**	**2.97E-02**	**-2.54**	**4.02E-06**	**3.38E-05**	*hutI*	imidazolonepropionase
EAM_1259	**-3.74**	**8.76E-16**	**9.55E-13**	**-4.03**	**1.40E-17**	**3.57E-15**	*hutG*	N-formylglutamate amidohydrolase
EAM_1260	0.66	1.15E-02	6.10E-02	**1.06**	**4.73E-05**	**2.85E-04**	-	5'(3')-deoxyribonucleotidase
EAM_1263	-0.26	6.08E-01	8.40E-01	**-1.26**	**1.38E-02**	**3.12E-02**	-	hypothetical protein
EAM_1264	-0.51	3.56E-01	6.54E-01	**-1.37**	**1.41E-02**	**3.18E-02**	-	pseudo

EAM_1267	-0.27	5.84E-01	8.24E-01	**-1.20**	**1.65E-02**	**3.56E-02**	-	hypothetical protein
EAM_1271	-0.85	3.57E-03	2.48E-02	**-1.10**	**1.75E-04**	**9.01E-04**	-	Zinc-binding protein
EAM_1272	**-1.41**	**1.02E-05**	**2.49E-04**	**-2.16**	**3.87E-11**	**1.14E-09**	*dps*	DNA protection during starvation protein
EAM_1274	0.18	7.00E-01	8.75E-01	**1.09**	**1.99E-02**	**4.07E-02**	*ompX*	outer membrane protein
EAM_1278	0.99	2.04E-04	2.61E-03	**1.08**	**5.47E-05**	**3.23E-04**	-	efflux pump outer membrane protein
EAM_1280	-0.49	2.27E-01	5.02E-01	**-1.15**	**5.03E-03**	**1.42E-02**	-	hypothetical protein
EAM_1283	-0.69	3.83E-04	4.29E-03	**-1.00**	**2.95E-07**	**3.49E-06**	*moeB*	molybdopterin biosynthesis protein
EAM_1294	0.33	2.39E-01	5.19E-01	**2.10**	**7.93E-13**	**4.00E-11**	*sdaC*	serine transporter
EAM_1295	-0.72	1.26E-01	3.52E-01	**-1.46**	**2.32E-03**	**7.63E-03**	-	membrane protein
EAM_1306	-0.44	1.93E-01	4.57E-01	**-1.22**	**3.31E-04**	**1.53E-03**	-	membrane protein
EAM_1309	0.23	4.62E-01	7.41E-01	**1.22**	**6.39E-05**	**3.71E-04**	*artQ*	arginine ABC transporter permease
EAM_1310	**1.19**	**6.34E-06**	**1.77E-04**	**1.09**	**3.35E-05**	**2.09E-04**	*artI*	arginine ABC transporter substrate-binding protein
EAM_1317	-0.68	2.09E-03	1.67E-02	**-1.39**	**6.34E-10**	**1.39E-08**	-	hypothetical protein
EAM_1318	**1.13**	**6.31E-03**	**3.84E-02**	**2.43**	**1.77E-08**	**2.85E-07**	*cspD*	cold shock-like protein
EAM_1322	0.75	2.05E-02	9.44E-02	**1.44**	**1.00E-05**	**7.43E-05**	*infA*	translation initiation factor IF-1
EAM_1328	0.61	1.69E-02	8.21E-02	**1.20**	**3.09E-06**	**2.72E-05**	*lrp*	leucine-responsive AsnC family transcriptional regulator
EAM_1329	0.62	1.54E-02	7.62E-02	**1.10**	**1.83E-05**	**1.24E-04**	*ftsK*	cell division protein
EAM_1330	**1.04**	**1.27E-03**	**1.11E-02**	**1.04**	**1.26E-03**	**4.67E-03**	*lolA*	outer membrane lipoprotein carrier protein
EAM_1336	0.62	7.36E-03	4.32E-02	**1.15**	**9.06E-07**	**9.49E-06**	-	hypothetical protein
EAM_1337	0.34	2.19E-01	4.93E-01	**1.25**	**7.36E-06**	**5.78E-05**	*serC*	phosphoserine aminotransferase
EAM_1339	0.42	1.12E-01	3.24E-01	**1.21**	**7.91E-06**	**6.09E-05**	*cmk*	cytidylate kinase
EAM_1340	0.19	6.53E-01	8.57E-01	**1.07**	**1.10E-02**	**2.63E-02**	*rpsA*	30S ribosomal protein S1
EAM_1342	-0.44	2.89E-01	5.81E-01	**-1.38**	**9.49E-04**	**3.71E-03**	-	competence-like protein
EAM_1343	0.71	5.97E-04	6.05E-03	**1.30**	**6.83E-10**	**1.48E-08**	*msbA*	lipid A export ATP-binding/permease
EAM_1344	0.47	5.97E-02	2.05E-01	**1.03**	**3.97E-05**	**2.43E-04**	*lpxK*	tetraacyldisaccharide 4'-kinase
EAM_1345	0.35	4.27E-01	7.16E-01	**1.16**	**8.93E-03**	**2.26E-02**	-	hypothetical protein
EAM_1357	**2.06**	**6.63E-10**	**7.92E-08**	**3.43**	**2.00E-22**	**1.43E-19**	*ompF*	outer membrane porin
EAM_1358	0.72	8.47E-03	4.81E-02	**1.54**	**3.48E-08**	**5.33E-07**	*asnS*	asparaginyl-tRNA synthetase
EAM_1361	-0.54	1.66E-01	4.18E-01	**-1.23**	**1.87E-03**	**6.39E-03**	*ssuB*	aliphatic sulfonates ABC transporter ATP-binding protein
EAM_1362	-0.36	3.29E-01	6.28E-01	**-1.13**	**2.19E-03**	**7.31E-03**	*ssuC*	aliphatic sulfonates ABC transporter permease
EAM_1363	-0.17	6.73E-01	8.65E-01	**-1.28**	**1.50E-03**	**5.35E-03**	*ssuD*	alkanesulfonate monooxygenase

EAM_1369	0.70	4.50E-03	2.92E-02	**1.10**	**8.99E-06**	**6.77E-05**	-	RNA methylase
EAM_1370	0.95	1.85E-04	2.42E-03	**1.11**	**1.22E-05**	**8.83E-05**	*uup*	ABC transporter ATP-binding protein
EAM_1373	0.74	7.88E-03	4.57E-02	**1.18**	**2.41E-05**	**1.57E-04**	-	lipoprotein
EAM_1374	0.54	1.49E-02	7.43E-02	**1.14**	**3.84E-07**	**4.40E-06**	*fabA*	3-hydroxydecanoyl-ACP dehydratase
EAM_1375	0.51	3.78E-02	1.47E-01	**1.13**	**4.13E-06**	**3.45E-05**	-	protease La-like protein
EAM_1377	**1.45**	**3.81E-03**	**2.60E-02**	**2.12**	**3.62E-05**	**2.24E-04**	*ompA*	outer membrane protein A
EAM_1379	-0.47	4.11E-01	7.04E-01	**-1.75**	**3.08E-03**	**9.59E-03**	-	hypothetical protein
EAM_1388	-0.68	1.75E-01	4.29E-01	**-1.73**	**7.33E-04**	**2.99E-03**	-	acylphosphatase
EAM_1392	**-1.25**	**6.52E-04**	**6.51E-03**	**-1.28**	**4.76E-04**	**2.07E-03**	-	aldehyde dehydrogenase
EAM_1393	**-1.38**	**4.50E-05**	**7.86E-04**	**-1.66**	**9.88E-07**	**1.03E-05**	-	hypothetical protein
EAM_1394	-0.65	1.83E-01	4.43E-01	**-1.54**	**2.31E-03**	**7.63E-03**	-	hypothetical protein
EAM_1399	**-1.19**	**9.98E-04**	**9.08E-03**	**-1.16**	**1.29E-03**	**4.76E-03**	-	hypothetical protein
EAM_1407	-0.32	4.58E-01	7.38E-01	**-1.42**	**1.31E-03**	**4.84E-03**	-	transposase
EAM_1418	0.86	4.00E-03	2.69E-02	**1.40**	**3.78E-06**	**3.23E-05**	*mdoH*	glucans biosynthesis glucosyl transferase H
EAM_1422	0.68	2.98E-02	1.24E-01	**1.41**	**9.22E-06**	**6.89E-05**	-	rhodanese-like protein
EAM_1430	0.97	1.27E-04	1.81E-03	**1.28**	**5.31E-07**	**5.88E-06**	*pyrC*	dihydroorotase
EAM_1434	-0.18	6.91E-01	8.72E-01	**-1.02**	**2.22E-02**	**4.42E-02**	-	hypothetical protein
EAM_1435	0.22	5.13E-01	7.84E-01	**1.01**	**2.34E-03**	**7.69E-03**	*mviN*	membrane protein
EAM_1438	-0.53	6.61E-02	2.21E-01	**-1.08**	**2.32E-04**	**1.13E-03**	*flgA*	flagella basal body P-ring formation protein
EAM_1446	-0.28	5.98E-01	8.35E-01	**-1.23**	**1.98E-02**	**4.06E-02**	*flgI*	flagellar P-ring protein
EAM_1453	1.13	2.14E-02	9.81E-02	**2.15**	**2.15E-05**	**1.42E-04**	-	hypothetical protein
EAM_1454	**1.38**	**2.69E-03**	**2.02E-02**	**2.24**	**1.88E-06**	**1.78E-05**	*rpmF*	50S ribosomal protein L32
EAM_1455	**1.22**	**3.81E-04**	**4.28E-03**	**2.30**	**7.52E-11**	**2.06E-09**	*plsX*	fatty acid/phospholipid synthesis protein
EAM_1456	**1.60**	**2.05E-06**	**6.55E-05**	**2.84**	**6.58E-16**	**8.42E-14**	*fabH*	3-oxoacyl-ACP synthase
EAM_1457	**1.53**	**9.80E-06**	**2.42E-04**	**2.55**	**1.10E-12**	**5.31E-11**	*fabD*	malonyl CoA-acyl carrier protein transacylase
EAM_1458	0.36	2.55E-01	5.39E-01	**1.38**	**1.38E-05**	**9.76E-05**	*fabG*	3-oxoacyl-ACP reductase
EAM_1459	0.26	4.40E-01	7.26E-01	**1.42**	**3.90E-05**	**2.40E-04**	*acpP*	acyl carrier protein
EAM_1460	**1.33**	**8.61E-05**	**1.35E-03**	**2.64**	**8.04E-14**	**5.05E-12**	*fabF*	3-oxoacyl-ACP synthase
EAM_1466	**1.42**	**6.19E-05**	**1.03E-03**	**2.62**	**1.37E-12**	**6.54E-11**	*ptsG*	PTS system glucose-specific transporter subunit IIBC
EAM_1472	-0.49	3.47E-01	6.47E-01	**-1.35**	**1.04E-02**	**2.53E-02**	-	hypothetical protein
EAM_1476	-0.72	3.27E-03	2.34E-02	**-1.15**	**3.37E-06**	**2.91E-05**	-	hypothetical protein
EAM_1477	**-1.05**	**3.04E-03**	**2.23E-02**	**-1.35**	**1.46E-04**	**7.75E-04**	*yedP*	mannosyl-3-phosphoglycerate phosphatase

EAM_1478	-0.24	6.59E-01	8.59E-01	**-1.49**	**7.13E-03**	**1.88E-02**	-	hypothetical protein
EAM_1479	**-1.02**	**9.02E-05**	**1.39E-03**	**-1.33**	**3.94E-07**	**4.49E-06**	-	hypothetical protein
EAM_1483	-0.64	1.44E-01	3.85E-01	**-1.60**	**3.95E-04**	**1.76E-03**	-	membrane protein
EAM_1484	-0.43	3.38E-01	6.38E-01	**-1.74**	**1.79E-04**	**9.16E-04**	*fliR*	flagellar biosynthetic protein FliR
EAM_1486	-0.71	1.67E-01	4.19E-01	**-1.22**	**1.75E-02**	**3.70E-02**	*fliP*	flagellar biosynthetic protein
EAM_1493	-0.29	5.73E-01	8.16E-01	**-1.18**	**2.41E-02**	**4.69E-02**	*fliI*	flagellum-specific ATP synthase
EAM_1498	-0.44	6.28E-02	2.14E-01	**-1.01**	**2.25E-05**	**1.48E-04**	-	chemotaxis two-component response regulator
EAM_1499	-0.50	5.21E-02	1.84E-01	**-1.39**	**1.58E-07**	**2.00E-06**	-	hypothetical protein
EAM_1501	-0.53	2.61E-01	5.46E-01	**-1.57**	**9.20E-04**	**3.60E-03**	-	antitermination Protein
EAM_1502	-0.80	1.80E-01	4.39E-01	**-1.84**	**2.62E-03**	**8.39E-03**	-	hypothetical protein
EAM_1504	-0.42	3.92E-01	6.86E-01	**-1.58**	**1.52E-03**	**5.40E-03**	*edcC*	signal transduction protein
EAM_1506	0.69	2.72E-02	1.17E-01	**1.38**	**1.22E-05**	**8.83E-05**	*lolD*	lipoprotein-releasing system ATP-binding protein
EAM_1520	0.30	3.28E-01	6.26E-01	**1.57**	**6.49E-07**	**7.02E-06**	*icdA*	isocitrate dehydrogenase
EAM_1521	-0.77	1.71E-01	4.22E-01	**-1.81**	**1.57E-03**	**5.54E-03**	-	integrase (partial)
EAM_1523	-0.51	3.32E-01	6.31E-01	**-1.65**	**2.38E-03**	**7.77E-03**	-	Mig-14 family protein
EAM_1524	-0.57	3.93E-01	6.86E-01	**-1.68**	**1.38E-02**	**3.13E-02**	-	phage holin
EAM_1531	-0.53	2.23E-01	4.97E-01	**-1.11**	**1.22E-02**	**2.86E-02**	-	pseudo
EAM_1534	0.44	1.51E-01	3.95E-01	**1.15**	**1.65E-04**	**8.66E-04**	*argC*	N-acetyl-gamma-glutamyl-phosphate reductase
EAM_1535	-0.38	2.85E-01	5.76E-01	**-1.26**	**4.57E-04**	**1.99E-03**	-	pseudo
EAM_1544	**-1.06**	**2.95E-03**	**2.18E-02**	**-1.42**	**7.33E-05**	**4.18E-04**	-	hypothetical protein
EAM_1548	-0.40	5.92E-01	8.31E-01	**-2.03**	**8.61E-03**	**2.19E-02**	-	hypothetical protein
EAM_1549	-0.44	5.37E-01	7.96E-01	**-1.68**	**2.00E-02**	**4.08E-02**	-	transcriptional regulator
EAM_1550	-0.72	1.88E-01	4.49E-01	**-1.85**	**9.66E-04**	**3.76E-03**	-	type III secretion system protein
EAM_1552	-0.60	2.97E-01	5.91E-01	**-1.58**	**7.07E-03**	**1.87E-02**	-	type III secretion system lipoprotein
EAM_1553	-0.82	1.85E-01	4.45E-01	**-1.82**	**3.65E-03**	**1.10E-02**	-	pseudo
EAM_1554	-0.47	4.97E-01	7.66E-01	**-1.67**	**1.83E-02**	**3.81E-02**	-	type III secretion system protein
EAM_1555	-0.69	8.84E-02	2.73E-01	**-1.59**	**1.28E-04**	**6.90E-04**	-	type III secretion system protein
EAM_1556	-0.64	2.25E-01	5.00E-01	**-1.54**	**3.96E-03**	**1.17E-02**	-	regulator of type III secretion system
EAM_1557	-0.57	3.37E-01	6.37E-01	**-1.69**	**5.84E-03**	**1.60E-02**	-	type III secretion system protein
EAM_1558	-0.48	4.72E-01	7.50E-01	**-1.57**	**2.23E-02**	**4.43E-02**	-	type III secretion system protein
EAM_1559	-0.64	2.89E-01	5.81E-01	**-1.67**	**6.92E-03**	**1.85E-02**	-	type III secretion system protein
EAM_1560	-0.47	4.77E-01	7.53E-01	**-1.62**	**1.61E-02**	**3.48E-02**	-	type III secretion system protein
EAM_1561	-0.34	2.81E-01	5.72E-01	**-1.08**	**8.18E-04**	**3.26E-03**	-	type III secretion system protein

EAM_1563	-0.47	4.00E-01	6.92E-01	**-1.60**	**5.18E-03**	**1.46E-02**	-	type III secretion system protein
EAM_1564	-0.40	5.11E-01	7.81E-01	**-1.59**	**1.14E-02**	**2.70E-02**	-	type III secretion system protein
EAM_1565	-0.69	2.98E-01	5.92E-01	**-1.91**	**4.91E-03**	**1.40E-02**	-	type III secretion system protein
EAM_1567	-0.36	6.18E-01	8.41E-01	**-1.77**	**1.79E-02**	**3.76E-02**	-	type III secretion system protein
EAM_1568	-0.66	3.74E-01	6.70E-01	**-1.73**	**2.25E-02**	**4.45E-02**	-	type III secretion system protein
EAM_1569	-0.46	4.11E-01	7.04E-01	**-1.39**	**1.42E-02**	**3.19E-02**	-	type III secretion system chaperone protein
EAM_1571	-0.20	6.00E-01	8.36E-01	**-1.06**	**5.92E-03**	**1.62E-02**	-	type III secretion system protein
EAM_1572	-0.44	3.14E-01	6.11E-01	**-1.21**	**6.37E-03**	**1.72E-02**	-	type III secretion system protein
EAM_1573	-0.53	2.66E-01	5.53E-01	**-1.50**	**1.80E-03**	**6.19E-03**	-	type III secretion system protein
EAM_1574	-0.62	4.17E-01	7.07E-01	**-1.83**	**1.95E-02**	**4.00E-02**	-	type III secretion system protein
EAM_1575	-0.49	4.74E-01	7.52E-01	**-1.74**	**1.26E-02**	**2.93E-02**	-	type III secretion system protein
EAM_1577	-0.19	5.94E-01	8.32E-01	**-1.05**	**4.57E-03**	**1.32E-02**	-	sulfate transporter
EAM_1578	-0.06	9.14E-01	9.67E-01	**-1.40**	**9.03E-04**	**3.56E-03**	-	hypothetical protein
EAM_1582	**1.18**	**5.91E-04**	**6.03E-03**	**2.04**	**7.31E-09**	**1.30E-07**	*prsA*	ribose-phosphate pyrophosphokinase
EAM_1585	0.43	1.53E-01	3.97E-01	**1.14**	**1.72E-04**	**8.90E-04**	*hemA*	glutamyl-tRNA reductase
EAM_1587	0.87	1.39E-02	7.01E-02	**1.39**	**1.06E-04**	**5.80E-04**	*hemK*	protein methyltransferase
EAM_1590	0.78	9.31E-03	5.22E-02	**1.11**	**2.14E-04**	**1.05E-03**	*kdsA*	2-dehydro-3-deoxyphosphooctonate aldolase
EAM_1605	-0.43	1.58E-01	4.05E-01	**1.06**	**5.99E-04**	**2.52E-03**	*ompF*	outer membrane protein F
EAM_1606	**-2.31**	**3.02E-07**	**1.39E-05**	**-2.13**	**2.10E-06**	**1.95E-05**	*astC*	succinylornithine transaminase
EAM_1607	**-2.25**	**2.21E-07**	**1.09E-05**	**-2.09**	**1.39E-06**	**1.38E-05**	*astA*	arginine N-succinyltransferase
EAM_1608	**-2.20**	**2.56E-07**	**1.19E-05**	**-1.98**	**3.14E-06**	**2.75E-05**	*astD*	N-succinylglutamate 5-semialdehyde dehydrogenase
EAM_1609	**-2.10**	**2.51E-07**	**1.18E-05**	**-1.70**	**2.27E-05**	**1.49E-04**	*astB*	N-succinylarginine dihydrolase
EAM_1610	**-1.67**	**1.33E-06**	**4.53E-05**	**-1.29**	**1.71E-04**	**8.88E-04**	*astE*	succinylglutamate desuccinylase
EAM_1611	-0.97	6.78E-03	4.04E-02	**-1.07**	**2.96E-03**	**9.28E-03**	-	hypothetical protein
EAM_1612	**-2.72**	**7.92E-06**	**2.06E-04**	**-2.23**	**1.84E-04**	**9.38E-04**	*spy*	spheroplast protein Y
EAM_1617	**-1.95**	**2.57E-05**	**5.26E-04**	**-3.47**	**1.63E-12**	**7.48E-11**	-	membrane protein
EAM_1620	0.07	9.04E-01	9.64E-01	**-1.41**	**7.67E-03**	**2.00E-02**	-	hypothetical protein
EAM_1622	**-1.62**	**1.40E-05**	**3.20E-04**	**-1.62**	**1.39E-05**	**9.79E-05**	-	phosphotransferase
EAM_1623	-0.97	1.90E-04	2.47E-03	**-1.55**	**4.30E-09**	**8.07E-08**	-	hypothetical protein
EAM_1624	**1.08**	**3.27E-04**	**3.80E-03**	**1.85**	**1.53E-09**	**3.17E-08**	-	hypothetical protein
EAM_1625	-0.62	1.61E-01	4.10E-01	**-1.12**	**1.13E-02**	**2.70E-02**	-	hypothetical protein
EAM_1626	-0.46	1.36E-01	3.70E-01	**-1.05**	**9.69E-04**	**3.77E-03**	-	hypothetical protein

EAM_1628	0.46	1.89E-01	4.52E-01	**1.05**	**3.14E-03**	**9.73E-03**	*infC*	translation initiation factor IF-3
EAM_1629	0.67	1.93E-01	4.57E-01	**2.28**	**2.30E-05**	**1.50E-04**	*rpmI*	50S ribosomal protein L35
EAM_1630	0.79	1.41E-01	3.80E-01	**2.26**	**5.11E-05**	**3.06E-04**	*rplT*	50S ribosomal protein L20
EAM_1631	0.67	2.62E-03	1.99E-02	**1.34**	**3.10E-09**	**6.04E-08**	*pheS*	phenylalanyl-tRNA synthetase subunit alpha
EAM_1637	-0.41	3.58E-01	6.56E-01	**-1.08**	**1.70E-02**	**3.63E-02**	-	hypothetical protein
EAM_1643	-0.57	2.60E-01	5.45E-01	**-2.15**	**5.62E-05**	**3.30E-04**	-	pseudo
EAM_1644	-1.06	7.14E-02	2.33E-01	**-1.80**	**2.42E-03**	**7.87E-03**	-	hypothetical protein
EAM_1649	**-1.20**	**1.36E-03**	**1.19E-02**	**-1.46**	**1.13E-04**	**6.16E-04**	-	hypothetical protein
EAM_1659	-0.26	4.26E-01	7.16E-01	**-1.06**	**1.14E-03**	**4.30E-03**	*ribE*	riboflavin synthase subunit alpha
EAM_1662	-0.59	1.14E-01	3.27E-01	**-1.18**	**1.84E-03**	**6.31E-03**	-	LysR family transcriptional regulator
EAM_1666	0.70	1.26E-02	6.50E-02	**1.01**	**3.33E-04**	**1.54E-03**	*rnt*	ribonuclease T
EAM_1669	**-1.43**	**6.51E-05**	**1.06E-03**	**-1.04**	**3.28E-03**	**1.01E-02**	-	transcriptional regulator
EAM_1674	-0.09	7.34E-01	8.91E-01	**-1.01**	**2.02E-04**	**1.01E-03**	-	hypothetical protein
EAM_1675	-0.89	1.55E-03	1.32E-02	**-1.52**	**9.17E-08**	**1.24E-06**	-	transposase (partial)
EAM_1696	-0.58	2.40E-01	5.19E-01	**-1.74**	**5.22E-04**	**2.24E-03**	*araA*	L-arabinose isomerase
EAM_1697	-0.38	4.82E-01	7.58E-01	**-1.37**	**1.30E-02**	**2.99E-02**	*araB*	L-ribulokinase
EAM_1700	-0.39	2.78E-01	5.68E-01	**-1.17**	**1.42E-03**	**5.14E-03**	*araH*	L-arabinose ABC transporter permease
EAM_1707	**1.65**	**3.24E-07**	**1.44E-05**	**2.78**	**1.17E-16**	**1.91E-14**	-	lipoprotein
EAM_1708	-0.18	6.30E-01	8.49E-01	**-1.25**	**7.36E-04**	**3.00E-03**	-	major facilitator superfamily protein
EAM_1717	**1.28**	**7.52E-06**	**1.98E-04**	**1.76**	**1.34E-09**	**2.79E-08**	*dcp*	peptidyl-dipeptidase
EAM_1718	-0.27	4.24E-01	7.14E-01	**-1.06**	**2.28E-03**	**7.55E-03**	-	hydroxylase
EAM_1720	-0.66	1.23E-02	6.45E-02	**-1.17**	**1.07E-05**	**7.87E-05**	-	hypothetical protein
EAM_1722	-0.53	6.09E-03	3.74E-02	**-1.03**	**1.28E-07**	**1.66E-06**	-	hypothetical protein
EAM_1723	0.47	1.62E-01	4.12E-01	**2.28**	**5.68E-11**	**1.60E-09**	-	glutamate/leucine/phenylalanine/valine dehydrogenase
EAM_1724	0.81	2.03E-02	9.37E-02	**1.78**	**6.08E-07**	**6.65E-06**	-	short-chain alcohol dehydrogenase
EAM_1725	-0.60	8.79E-02	2.73E-01	**-1.31**	**2.35E-04**	**1.14E-03**	-	hypothetical protein
EAM_1726	-0.60	7.53E-02	2.43E-01	**-1.09**	**1.27E-03**	**4.69E-03**	-	TonB-dependent receptor
EAM_1730	-0.79	2.02E-01	4.69E-01	**-2.09**	**1.15E-03**	**4.32E-03**	-	lipoprotein
EAM_1731	-0.52	3.09E-01	6.07E-01	**-1.52**	**3.75E-03**	**1.12E-02**	-	1-phosphatidylinositol phosphodiesterase
EAM_1732	-0.47	3.84E-01	6.78E-01	**-1.70**	**2.15E-03**	**7.22E-03**	-	hypothetical protein
EAM_1739	-0.44	5.94E-01	8.32E-01	**-1.95**	**2.16E-02**	**4.32E-02**	-	hypothetical protein
EAM_1743	**-1.16**	**3.50E-05**	**6.51E-04**	**-1.54**	**6.20E-08**	**8.81E-07**	-	hypothetical protein

EAM_1749	-0.78	3.25E-05	6.26E-04	**-1.09**	**9.75E-09**	**1.68E-07**	-	monooxygenase
EAM_1750	-0.84	1.64E-05	3.64E-04	**-1.29**	**4.80E-11**	**1.37E-09**	*pvcB*	siderophore biosynthesis protein
EAM_1751	**-1.05**	**1.10E-04**	**1.61E-03**	**-1.75**	**2.77E-10**	**6.53E-09**	*pvcA*	siderophore biosynthesis protein
EAM_1757	-0.47	1.87E-01	4.48E-01	**-1.05**	**2.99E-03**	**9.37E-03**	*glsA*	glutaminase
EAM_1759	-0.70	1.23E-01	3.46E-01	**-1.56**	**8.02E-04**	**3.20E-03**	-	acyl-CoA dehydrogenase
EAM_1761	-0.55	2.42E-01	5.21E-01	**-1.71**	**4.24E-04**	**1.88E-03**	-	hypothetical protein
EAM_1762	-0.42	4.26E-01	7.16E-01	**-1.63**	**2.80E-03**	**8.85E-03**	-	FAD-dependent oxidoreductase
EAM_1763	-0.75	2.87E-02	1.21E-01	**-1.39**	**5.84E-05**	**3.43E-04**	*sfnG*	FMNH2-dependent dimethyl sulfone monooxygenase
EAM_1764	-0.67	9.95E-02	2.97E-01	**-1.69**	**5.26E-05**	**3.13E-04**	*msuD*	methanesulfonate monooxygenase
EAM_1768	-0.42	4.63E-01	7.42E-01	**-1.51**	**1.04E-02**	**2.53E-02**	-	hypothetical protein
EAM_1772	**1.03**	**1.51E-04**	**2.06E-03**	**1.26**	**3.89E-06**	**3.31E-05**	-	acetyltransferase
EAM_1773	-0.33	3.95E-01	6.88E-01	**-1.25**	**1.58E-03**	**5.55E-03**	-	hypothetical protein
EAM_1780	-0.37	3.37E-01	6.37E-01	**-1.15**	**3.49E-03**	**1.06E-02**	-	hypothetical protein
EAM_1781	0.91	1.78E-04	2.36E-03	**1.58**	**1.34E-10**	**3.37E-09**	-	hypothetical protein
EAM_1789	-0.39	5.33E-01	7.95E-01	**-1.56**	**1.48E-02**	**3.28E-02**	-	tail fiber like-protein
EAM_1790	-0.63	1.23E-01	3.46E-01	**-1.21**	**3.33E-03**	**1.02E-02**	-	hypothetical protein
EAM_1791	-0.41	3.87E-01	6.81E-01	**-1.29**	**7.02E-03**	**1.86E-02**	-	hypothetical protein
EAM_1792	-0.49	5.30E-01	7.94E-01	**-2.04**	**1.18E-02**	**2.79E-02**	-	hypothetical protein
EAM_1793	-0.52	4.05E-01	6.98E-01	**-1.68**	**7.84E-03**	**2.03E-02**	-	hypothetical protein
EAM_1794	-0.80	1.13E-01	3.26E-01	**-1.82**	**4.14E-04**	**1.84E-03**	-	hypothetical protein
EAM_1795	-0.18	7.23E-01	8.88E-01	**-1.19**	**2.02E-02**	**4.10E-02**	-	phage protein
EAM_1796	-0.86	1.56E-01	4.01E-01	**-2.16**	**5.80E-04**	**2.45E-03**	-	hypothetical protein
EAM_1797	-0.34	5.47E-01	8.02E-01	**-1.72**	**3.38E-03**	**1.03E-02**	-	hypothetical protein
EAM_1798	-0.46	2.13E-01	4.84E-01	**-1.11**	**3.03E-03**	**9.47E-03**	-	acyltransferase
EAM_1799	-0.62	3.02E-01	5.97E-01	**-1.78**	**4.05E-03**	**1.19E-02**	-	phage tail fiber protein
EAM_1800	-0.62	2.99E-01	5.93E-01	**-1.81**	**3.09E-03**	**9.62E-03**	-	phage protein
EAM_1801	-0.62	2.41E-01	5.21E-01	**-1.80**	**9.92E-04**	**3.84E-03**	-	integrase (partial)
EAM_1804	-0.34	6.43E-01	8.53E-01	**-2.26**	**2.89E-03**	**9.12E-03**	-	hypothetical protein
EAM_1805	**-1.35**	**5.89E-04**	**6.03E-03**	**-1.06**	**6.44E-03**	**1.74E-02**	*asr*	acid shock protein
EAM_1806	-0.89	1.61E-01	4.10E-01	**-1.58**	**1.45E-02**	**3.25E-02**	-	hypothetical protein
EAM_1811	-1.10	1.27E-02	6.54E-02	**-1.35**	**2.14E-03**	**7.20E-03**	-	hypothetical protein
EAM_1812	**-1.21**	**1.02E-04**	**1.54E-03**	**-1.09**	**4.51E-04**	**1.98E-03**	-	short-chain dehydrogenase

EAM_1818	-0.50	1.24E-02	6.46E-02	**-1.20**	**2.64E-09**	**5.26E-08**	-	monooxygenase
EAM_1820	**-1.46**	**7.59E-07**	**2.70E-05**	**-2.51**	**2.21E-16**	**3.16E-14**	-	hypothetical protein
EAM_1822	0.20	3.49E-01	6.48E-01	**1.11**	**4.10E-07**	**4.66E-06**	-	hypothetical protein
EAM_1826	-0.57	4.11E-01	7.04E-01	**-1.88**	**8.30E-03**	**2.13E-02**	-	fimbrial chaperone protein
EAM_1827	-0.39	2.66E-01	5.53E-01	**-1.18**	**7.94E-04**	**3.17E-03**	-	outer membrane fimbrial usher protein
EAM_1833	0.82	1.54E-02	7.62E-02	**1.23**	**2.92E-04**	**1.37E-03**	*tpx*	thiol peroxidase
EAM_1835	0.17	6.04E-01	8.38E-01	**1.18**	**2.61E-04**	**1.25E-03**	-	membrane protein
EAM_1836	0.74	1.80E-02	8.62E-02	**2.40**	**1.93E-13**	**1.08E-11**	-	ATP-binding protein
EAM_1837	-0.11	7.84E-01	9.15E-01	**1.34**	**1.08E-03**	**4.11E-03**	*pspD*	phage shock protein D
EAM_1838	-0.31	4.42E-01	7.27E-01	**1.26**	**2.01E-03**	**6.81E-03**	*pspC*	phage shock protein C
EAM_1839	1.07	2.39E-02	1.07E-01	**3.52**	**7.48E-12**	**2.76E-10**	*pspB*	phage shock protein B
EAM_1840	**1.29**	**8.27E-03**	**4.75E-02**	**3.62**	**9.35E-12**	**3.25E-10**	*pspA*	phage shock protein A
EAM_1847	-0.93	6.08E-03	3.74E-02	**-1.80**	**2.54E-07**	**3.07E-06**	-	hypothetical protein
EAM_1855	0.57	4.13E-02	1.56E-01	**1.48**	**1.46E-07**	**1.87E-06**	*rnb*	exoribonuclease II
EAM_1856	-0.99	2.23E-04	2.78E-03	**-1.49**	**4.01E-08**	**6.09E-07**	*araD*	L-ribulose-5-phosphate 4-epimerase
EAM_1857	**-1.70**	**2.71E-08**	**1.83E-06**	**-2.03**	**5.22E-11**	**1.49E-09**	*osmB*	osmotically inducible lipoprotein B
EAM_1858	**-1.67**	**2.37E-08**	**1.73E-06**	**-2.05**	**1.18E-11**	**3.99E-10**	-	hypothetical protein
EAM_1859	**-1.01**	**8.36E-05**	**1.31E-03**	**-1.54**	**3.04E-09**	**5.95E-08**	-	hypothetical protein
EAM_1861	**1.09**	**5.69E-03**	**3.52E-02**	**2.16**	**8.65E-08**	**1.17E-06**	*pyrF*	Orotidine 5'-phosphate decarboxylase
EAM_1867	**-2.58**	**1.19E-07**	**6.28E-06**	**-2.05**	**1.72E-05**	**1.18E-04**	-	hypothetical protein
EAM_1868	**-1.95**	**6.59E-08**	**4.16E-06**	**-1.71**	**1.81E-06**	**1.73E-05**	-	hypothetical protein
EAM_1873	0.60	1.50E-01	3.92E-01	**1.08**	**1.04E-02**	**2.53E-02**	*rluB*	ribosomal large subunit pseudouridine synthase B
EAM_1884	**2.88**	**2.46E-08**	**1.73E-06**	**1.75**	**4.01E-04**	**1.78E-03**	*ompW*	outer membrane protein W
EAM_1894	0.66	1.33E-02	6.79E-02	**1.29**	**1.28E-06**	**1.28E-05**	*oppD*	oligopeptide transport ATP-binding protein
EAM_1895	0.67	7.81E-03	4.55E-02	**1.72**	**1.96E-11**	**6.05E-10**	*oppC*	oligopeptide transport permease
EAM_1896	0.92	9.49E-04	8.72E-03	**1.95**	**5.10E-12**	**2.01E-10**	*oppB*	oligopeptide transport permease
EAM_1897	**1.31**	**1.19E-04**	**1.71E-03**	**1.47**	**1.71E-05**	**1.17E-04**	*oppA*	periplasmic oligopeptide-binding protein
EAM_1898	0.87	1.04E-03	9.38E-03	**1.30**	**1.11E-06**	**1.14E-05**	*oppA2*	periplasmic oligopeptide-binding protein
EAM_1906	-0.71	2.76E-01	5.66E-01	**-1.98**	**3.23E-03**	**9.97E-03**	-	N-acetylglucosaminyl-phosphatidylinositol de-N-acetylase
EAM_1907	-0.57	4.00E-01	6.92E-01	**-2.06**	**3.42E-03**	**1.04E-02**	-	demethylmenaquinone methyltransferase
EAM_1908	-0.43	4.26E-01	7.16E-01	**-1.75**	**1.41E-03**	**5.11E-03**	-	hypothetical protein
EAM_1909	-0.67	2.77E-01	5.66E-01	**-1.53**	**1.40E-02**	**3.16E-02**	-	NAD-binding oxidoreductase

EAM_1912	-0.49	2.36E-01	5.15E-01	**-1.22**	**3.38E-03**	**1.03E-02**	-	gluconate dehydrogenase subunit
EAM_1913	-0.42	2.68E-01	5.54E-01	**-1.10**	**4.21E-03**	**1.23E-02**	-	gluconate dehydrogenase cytochrome C subunit
EAM_1917	0.49	5.17E-02	1.83E-01	**1.01**	**7.63E-05**	**4.34E-04**	-	UDP-glucose/GDP-mannose dehydrogenase
EAM_1919	-0.65	1.55E-03	1.32E-02	**-1.24**	**2.50E-09**	**5.00E-08**	*hnr*	two-component response regulator
EAM_1927	0.56	1.12E-02	5.99E-02	**1.34**	**1.67E-09**	**3.44E-08**	*ansA*	L-Asparaginase I
EAM_1931	**1.86**	**3.18E-06**	**9.62E-05**	**1.92**	**1.71E-06**	**1.65E-05**	*gapA*	glyceraldehyde 3-phosphate dehydrogenase A
EAM_1932	**1.26**	**7.76E-06**	**2.03E-04**	**1.08**	**1.23E-04**	**6.66E-04**	-	aldose 1-epimerase
EAM_1933	-0.65	5.27E-04	5.52E-03	**-1.22**	**1.08E-10**	**2.80E-09**	-	aldo/keto reductase
EAM_1937	-0.94	8.76E-02	2.72E-01	**-1.70**	**2.36E-03**	**7.72E-03**	-	hypothetical protein
EAM_1939	-0.45	2.92E-01	5.84E-01	**-1.13**	**8.60E-03**	**2.19E-02**	-	hypothetical protein
EAM_1945	-0.49	1.07E-01	3.14E-01	**-1.33**	**1.71E-05**	**1.17E-04**	-	hypothetical protein
EAM_1967	1.16	1.86E-02	8.76E-02	**2.41**	**2.43E-06**	**2.21E-05**	*cspC*	cold shock-like protein
EAM_1968	0.08	8.63E-01	9.51E-01	**1.82**	**6.41E-05**	**3.71E-04**	-	hypothetical protein
EAM_1973	**1.14**	**5.36E-05**	**9.10E-04**	**1.51**	**1.26E-07**	**1.64E-06**	*prc*	tail-specific protease
EAM_1974	**1.24**	**5.12E-04**	**5.44E-03**	**1.81**	**5.58E-07**	**6.11E-06**	*proQ*	ProP effector protein
EAM_1979	-0.50	1.44E-02	7.22E-02	**-1.19**	**1.25E-08**	**2.09E-07**	-	hypothetical protein
EAM_1984	0.86	1.32E-02	6.76E-02	**1.20**	**5.82E-04**	**2.46E-03**	-	membrane protein
EAM_1993	-0.23	4.38E-01	7.24E-01	**-1.07**	**4.82E-04**	**2.09E-03**	-	hypothetical protein
EAM_1997	0.34	1.73E-01	4.25E-01	**1.35**	**6.17E-08**	**8.80E-07**	*cmr*	multidrug translocase (chloramphenicol resistance pump)
EAM_2010	-0.50	8.35E-02	2.63E-01	**-1.07**	**2.34E-04**	**1.13E-03**	-	membrane protein
EAM_2012	0.59	4.90E-02	1.76E-01	**1.18**	**1.02E-04**	**5.64E-04**	-	methyltransferase
EAM_2014	-0.70	2.76E-01	5.66E-01	**-2.11**	**1.47E-03**	**5.25E-03**	-	hypothetical protein
EAM_2019	-0.79	2.20E-01	4.94E-01	**-1.87**	**4.50E-03**	**1.30E-02**	*flhE*	flagellar protein FlhE
EAM_2020	-0.45	2.56E-01	5.40E-01	**-1.12**	**4.72E-03**	**1.35E-02**	*flhA*	flagellar biosynthesis protein FlhA
EAM_2021	-0.60	2.77E-01	5.67E-01	**-1.34**	**1.74E-02**	**3.69E-02**	*flhB*	flagellar biosynthetic protein FlhB
EAM_2024	-0.29	4.15E-01	7.06E-01	**-1.11**	**1.81E-03**	**6.21E-03**	*cheB*	Chemotaxis response regulator protein-glutamate methylesterase
EAM_2025	-0.45	3.63E-01	6.59E-01	**-1.50**	**3.22E-03**	**9.95E-03**	*cheR*	chemotaxis protein methyltransferase
EAM_2026	-0.54	1.39E-01	3.76E-01	**-1.13**	**2.32E-03**	**7.64E-03**	-	methyl-accepting chemotaxis protein
EAM_2028	-0.50	2.11E-01	4.81E-01	**-1.36**	**8.76E-04**	**3.47E-03**	*cheD*	methyl-accepting chemotaxis protein
EAM_2029	-0.51	8.97E-02	2.75E-01	**-1.13**	**2.06E-04**	**1.02E-03**	*cheW*	chemotaxis protein
EAM_2031	-0.41	2.65E-01	5.51E-01	**-1.06**	**4.50E-03**	**1.30E-02**	*motB*	chemotaxis protein

EAM_2034	**-1.19**	**7.63E-04**	**7.31E-03**	**-1.23**	**4.80E-04**	**2.09E-03**	*flhD*	flagellar transcriptional activator
EAM_2035	-0.92	4.48E-03	2.92E-02	**-1.09**	**7.27E-04**	**2.97E-03**	*otsA*	trehalose-6-phosphate synthase
EAM_2036	**-1.38**	**5.04E-05**	**8.68E-04**	**-1.10**	**1.09E-03**	**4.16E-03**	*otsB*	trehalose phosphatase
EAM_2037	-0.48	3.90E-01	6.84E-01	**-1.41**	**1.25E-02**	**2.91E-02**	-	hypothetical protein
EAM_2039	-0.77	8.13E-02	2.57E-01	**-1.30**	**3.66E-03**	**1.10E-02**	*umuD*	protein UmuD
EAM_2040	-0.54	1.01E-01	3.00E-01	**-1.04**	**1.52E-03**	**5.41E-03**	*umuC*	protein UmuC
EAM_2044	-0.56	1.51E-01	3.95E-01	**-1.15**	**3.57E-03**	**1.08E-02**	-	oligosaccharide translocase
EAM_2045	-0.49	4.78E-01	7.55E-01	**-1.74**	**1.43E-02**	**3.20E-02**	-	glycosyl transferase family protein
EAM_2046	-0.71	3.78E-01	6.74E-01	**-2.24**	**7.66E-03**	**2.00E-02**	-	hypothetical protein
EAM_2051	-0.38	4.83E-01	7.58E-01	**-1.78**	**1.45E-03**	**5.20E-03**	-	dehydrogenase (partial)
EAM_2052	-0.51	1.11E-01	3.21E-01	**-1.23**	**1.55E-04**	**8.18E-04**	*phoA*	alkaline phosphatase
EAM_2053	-0.68	2.22E-01	4.96E-01	**-2.00**	**5.14E-04**	**2.21E-03**	-	hypothetical protein
EAM_2054	-0.33	4.54E-01	7.35E-01	**-1.52**	**7.74E-04**	**3.11E-03**	-	pseudo
EAM_2057	0.89	9.44E-03	5.25E-02	**1.15**	**9.11E-04**	**3.58E-03**	-	peroxidase
EAM_2059	-0.17	6.26E-01	8.47E-01	**1.07**	**1.67E-03**	**5.80E-03**	-	permease
EAM_2060	0.69	3.64E-01	6.60E-01	**2.72**	**7.89E-04**	**3.16E-03**	*putP*	sodium/proline symporter
EAM_2061	1.38	1.18E-01	3.36E-01	**2.56**	**5.14E-03**	**1.45E-02**	*putA*	bifunctional proline dehydrogenase/delta-1-pyrroline-5-carboxylate dehydrogenase
EAM_2062	-0.69	1.29E-01	3.58E-01	**-1.47**	**1.41E-03**	**5.11E-03**	-	acyltransferase
EAM_2063	-0.62	2.13E-01	4.84E-01	**-1.62**	**1.41E-03**	**5.11E-03**	-	pseudo
EAM_2066	-0.51	3.56E-01	6.54E-01	**-1.65**	**3.42E-03**	**1.04E-02**	-	hypothetical protein
EAM_2069	-0.48	1.71E-01	4.23E-01	**-1.01**	**4.71E-03**	**1.35E-02**	*fliS*	flagellar protein FliS
EAM_2073	-0.54	9.24E-02	2.82E-01	**-1.41**	**1.51E-05**	**1.05E-04**	-	hypothetical protein
EAM_2074	-0.83	1.43E-01	3.85E-01	**-1.93**	**8.79E-04**	**3.48E-03**	-	hypothetical protein
EAM_2075	**-1.69**	**1.14E-05**	**2.70E-04**	**-2.07**	**1.08E-07**	**1.43E-06**	-	hypothetical protein
EAM_2076	-0.97	7.45E-06	1.98E-04	**-1.08**	**6.15E-07**	**6.69E-06**	-	membrane protein
EAM_2078	**-1.46**	**6.83E-03**	**4.07E-02**	**-1.34**	**1.26E-02**	**2.93E-02**	-	hypothetical protein
EAM_2079	**-1.13**	**6.30E-05**	**1.04E-03**	**-1.15**	**4.66E-05**	**2.81E-04**	*vsr*	DNA mismatch endonuclease
EAM_2085	-0.59	1.97E-01	4.62E-01	**-1.34**	**3.72E-03**	**1.12E-02**	-	hypothetical protein
EAM_2086	-0.40	2.23E-01	4.97E-01	**-1.27**	**1.18E-04**	**6.45E-04**	*katG*	peroxidase/catalase
EAM_2087	-1.13	1.83E-02	8.67E-02	**-2.52**	**5.11E-07**	**5.70E-06**	*sitA*	iron ABC transporter substrate-binding protein
EAM_2088	-0.94	3.65E-02	1.44E-01	**-2.28**	**1.16E-06**	**1.18E-05**	*sitB*	iron ABC transporter ATP-binding protein
EAM_2089	-0.99	6.60E-03	3.97E-02	**-1.81**	**1.20E-06**	**1.22E-05**	*sitC*	iron ABC transporter permease

EAM_2090	-0.89	7.73E-02	2.48E-01	**-1.41**	**5.71E-03**	**1.58E-02**	*sitD*	iron ABC transporter permease
EAM_2092	-0.58	2.89E-01	5.81E-01	**-1.80**	**1.44E-03**	**5.18E-03**	-	type III effector protein
EAM_2093	-0.58	3.83E-01	6.78E-01	**-1.94**	**4.56E-03**	**1.31E-02**	-	hypothetical protein
EAM_2097	-0.46	3.59E-01	6.56E-01	**-1.68**	**1.01E-03**	**3.90E-03**	-	ABC transporter ATP-binding protein
EAM_2098	-0.56	1.83E-01	4.43E-01	**-1.46**	**7.29E-04**	**2.98E-03**	-	ABC transporter permease
EAM_2099	-0.54	3.22E-01	6.20E-01	**-1.60**	**4.31E-03**	**1.26E-02**	-	ABC transporter permease
EAM_2100	-0.27	5.36E-01	7.96E-01	**-1.16**	**9.12E-03**	**2.30E-02**	-	ABC transporter substrate-binding protein
EAM_2101	-0.97	8.54E-04	7.95E-03	**-1.24**	**2.57E-05**	**1.66E-04**	-	hypothetical protein
EAM_2102	-1.01	1.18E-02	6.23E-02	**-1.57**	**1.10E-04**	**6.00E-04**	-	hypothetical protein
EAM_2104	-0.65	1.12E-01	3.23E-01	**-1.56**	**2.07E-04**	**1.02E-03**	-	hypothetical protein
EAM_2106	-0.57	2.61E-01	5.46E-01	**-1.20**	**2.01E-02**	**4.10E-02**	-	hypothetical protein
EAM_2109	-0.08	9.01E-01	9.63E-01	**-1.37**	**1.70E-02**	**3.63E-02**	-	hypothetical protein
EAM_2112	-0.04	9.16E-01	9.68E-01	**-1.24**	**1.66E-03**	**5.78E-03**	*nac*	nitrogen assimilation regulatory protein
EAM_2115	**1.13**	**5.35E-04**	**5.57E-03**	**1.56**	**2.25E-06**	**2.06E-05**	-	liporotein
EAM_2116	0.55	3.98E-01	6.91E-01	**2.23**	**8.15E-04**	**3.25E-03**	-	membrane protein
EAM_2117	**1.24**	**3.25E-08**	**2.16E-06**	**1.76**	**6.74E-15**	**5.62E-13**	-	membrane protein
EAM_2119	**-1.44**	**4.63E-09**	**4.15E-07**	**-2.13**	**2.03E-17**	**4.85E-15**	-	zinc-binding alcohol dehydrogenase
EAM_2121	-0.30	6.80E-01	8.68E-01	**-1.69**	**2.35E-02**	**4.60E-02**	-	hypothetical protein
EAM_2122	-0.77	4.92E-02	1.77E-01	**-1.56**	**8.52E-05**	**4.79E-04**	-	AsnC family transcriptional regulator
EAM_2123	-0.78	8.57E-02	2.68E-01	**-1.60**	**5.15E-04**	**2.21E-03**	-	hypothetical protein
EAM_2132	-0.73	1.56E-03	1.32E-02	**-1.06**	**4.57E-06**	**3.78E-05**	-	NADH:flavin oxidoreductase
EAM_2133	-0.75	6.39E-03	3.88E-02	**-1.16**	**2.79E-05**	**1.79E-04**	-	membrane protein
EAM_2134	0.86	6.28E-02	2.14E-01	**1.76**	**2.04E-04**	**1.01E-03**	-	hypothetical protein
EAM_2137	0.86	3.25E-03	2.34E-02	**1.81**	**1.26E-09**	**2.65E-08**	-	amino acid permease
EAM_2139	0.76	2.96E-02	1.24E-01	**1.53**	**1.69E-05**	**1.16E-04**	-	hypothetical protein
EAM_2145	0.66	2.02E-02	9.37E-02	**1.21**	**2.37E-05**	**1.54E-04**	*hisH*	imidazole glycerol phosphate synthase subunit
EAM_2146	0.46	8.38E-02	2.63E-01	**1.01**	**1.40E-04**	**7.48E-04**	*hisA*	1-(5-phosphoribosyl)-5-[(5-phosphoribosylamino)methylideneamino] imidazole-4-carboxamide isomerase
EAM_2148	0.74	2.93E-03	2.18E-02	**1.08**	**1.33E-05**	**9.50E-05**	*hisI*	bifunctional phosphoribosyl-AMP cyclohydrolase/phosphoribosyl-ATP pyrophosphatase
EAM_2149	0.92	3.19E-03	2.31E-02	**1.31**	**2.71E-05**	**1.74E-04**	-	hypothetical protein
EAM_2157	0.71	2.84E-02	1.20E-01	**1.18**	**2.69E-04**	**1.28E-03**	*rfbA*	O-antigen export system permease

EAM_2159	0.64	3.24E-03	2.34E-02	**1.06**	**1.26E-06**	**1.27E-05**	*rfbA*	glucose-1-phospate thymidyltransferase
EAM_2160	0.73	6.61E-03	3.97E-02	**1.25**	**4.00E-06**	**3.37E-05**	*rfbB*	dTDP-D-glucose-4,6-dehydratase
EAM_2161	0.70	6.68E-03	4.00E-02	**1.22**	**2.96E-06**	**2.63E-05**	*galE*	UDP-glucose 4-epimerase
EAM_2162	0.70	1.71E-02	8.27E-02	**1.28**	**1.67E-05**	**1.15E-04**	*galF*	UTP-glucose-1-phosphate uridylyltransferase
EAM_2168	-0.37	3.88E-01	6.82E-01	**-1.30**	**2.96E-03**	**9.28E-03**	*amsD*	amylovoran biosynthesis glycosyltransferase
EAM_2175	-0.47	1.09E-01	3.19E-01	**-1.15**	**1.05E-04**	**5.77E-04**	-	membrane protein
EAM_2177	0.71	3.54E-02	1.41E-01	**1.31**	**1.18E-04**	**6.45E-04**	*dcd*	deoxycytidine triphosphate deaminase
EAM_2178	0.58	2.91E-02	1.22E-01	**1.41**	**1.36E-07**	**1.76E-06**	*udk*	uridine kinase
EAM_2179	-0.26	5.29E-01	7.94E-01	**-1.02**	**1.44E-02**	**3.23E-02**	-	membrane protein
EAM_2183	-0.30	5.40E-01	7.96E-01	**-1.08**	**2.54E-02**	**4.89E-02**	*mdtA*	multidrug resistance protein
EAM_2184	-0.32	4.42E-01	7.27E-01	**-1.29**	**2.29E-03**	**7.60E-03**	*mdtB*	multidrug resistance protein
EAM_2185	-0.42	2.30E-01	5.07E-01	**-1.08**	**2.18E-03**	**7.27E-03**	*mdtC*	multidrug resistance protein
EAM_2187	-0.31	2.25E-01	5.00E-01	**-1.10**	**3.00E-05**	**1.90E-04**	*baeR*	two-component response regulator
EAM_2188	-0.48	2.01E-01	4.67E-01	**-1.46**	**1.52E-04**	**8.01E-04**	-	pseudo
EAM_2189	-0.97	1.60E-02	7.84E-02	**-2.02**	**1.09E-06**	**1.13E-05**	-	hypothetical protein
EAM_2190	-0.49	2.64E-01	5.49E-01	**-1.49**	**8.49E-04**	**3.37E-03**	-	hypothetical protein
EAM_2199	0.53	3.78E-02	1.47E-01	**1.12**	**1.38E-05**	**9.76E-05**	*metG*	methionyl-tRNA synthetase
EAM_2207	**1.18**	**7.28E-03**	**4.28E-02**	**1.60**	**3.10E-04**	**1.44E-03**	*mglB*	galactoside ABC transporter substrate-binding protein
EAM_2219	-0.37	4.49E-01	7.31E-01	**-1.81**	**2.81E-04**	**1.32E-03**	-	membrane protein
EAM_2223	0.64	2.51E-02	1.10E-01	**1.70**	**4.37E-09**	**8.11E-08**	*fruB*	multiphosphoryl transfer protein
EAM_2227	0.71	7.93E-02	2.53E-01	**1.99**	**1.55E-06**	**1.53E-05**	*spr*	lipoprotein
EAM_2237	**1.46**	**3.42E-05**	**6.45E-04**	**2.81**	**2.74E-14**	**1.87E-12**	*rplY*	50S ribosomal protein L25
EAM_2242	-0.56	2.13E-01	4.84E-01	**-1.74**	**1.65E-04**	**8.63E-04**	-	hypothetical protein
EAM_2246	-0.28	5.61E-01	8.10E-01	**-1.11**	**1.98E-02**	**4.06E-02**	-	hypothetical protein
EAM_2248	-0.22	6.67E-01	8.64E-01	**-1.27**	**1.52E-02**	**3.33E-02**	-	transposase (partial)
EAM_2249	-0.37	3.80E-01	6.76E-01	**-1.10**	**8.40E-03**	**2.15E-02**	-	transposase (partial)
EAM_2251	0.94	6.84E-04	6.73E-03	**1.27**	**4.72E-06**	**3.88E-05**	-	hypothetical protein
EAM_2253	-0.57	2.86E-01	5.76E-01	**-1.39**	**1.06E-02**	**2.57E-02**	-	hypothetical protein
EAM_2258	-0.62	1.10E-01	3.21E-01	**-1.50**	**1.90E-04**	**9.59E-04**	-	hypothetical protein
EAM_2266	**1.22**	**4.24E-07**	**1.76E-05**	**1.37**	**1.46E-08**	**2.41E-07**	*nrdA*	ribonucleoside-diphosphate reductase 1 subunit alpha
EAM_2268	**1.07**	**2.74E-03**	**2.05E-02**	**1.10**	**2.01E-03**	**6.82E-03**	-	ferredoxin

EAM_2274	0.70	1.64E-02	8.02E-02	**1.36**	**4.10E-06**	**3.43E-05**	*kup*	low affinity potassium transport system protein
EAM_2276	**1.26**	**1.23E-05**	**2.86E-04**	**1.66**	**1.03E-08**	**1.75E-07**	*mdoD*	glucans biosynthesis protein D
EAM_2279	-0.61	5.96E-02	2.05E-01	**-1.20**	**2.45E-04**	**1.18E-03**	-	membrane protein
EAM_2280	**1.41**	**1.41E-03**	**1.21E-02**	**2.38**	**1.70E-07**	**2.15E-06**	*nuoN*	NADH dehydrogenase I subunit N
EAM_2281	**1.34**	**1.03E-04**	**1.54E-03**	**2.00**	**1.35E-08**	**2.24E-07**	*nuoM*	NADH dehydrogenase I subunit M
EAM_2282	0.91	8.39E-03	4.80E-02	**1.67**	**1.94E-06**	**1.83E-05**	*nuoL*	NADH dehydrogenase I subunit L
EAM_2283	0.87	1.04E-03	9.40E-03	**1.24**	**3.23E-06**	**2.81E-05**	*nuoK*	NADH dehydrogenase I subunit K
EAM_2284	0.94	3.00E-03	2.20E-02	**1.36**	**2.03E-05**	**1.35E-04**	*nuoJ*	NADH dehydrogenase I subunit J
EAM_2285	**1.03**	**1.20E-03**	**1.07E-02**	**1.47**	**4.76E-06**	**3.90E-05**	*nuoI*	NADH dehydrogenase I subunit I
EAM_2286	**1.10**	**8.84E-03**	**4.99E-02**	**2.01**	**2.77E-06**	**2.47E-05**	*nuoH*	NADH dehydrogenase I subunit H
EAM_2287	**1.21**	**6.52E-04**	**6.51E-03**	**1.85**	**3.25E-07**	**3.79E-06**	*nuoG*	NADH dehydrogenase I subunit G
EAM_2288	**1.13**	**1.79E-04**	**2.36E-03**	**1.67**	**5.31E-08**	**7.80E-07**	*nuoF*	NADH dehydrogenase I subunit F
EAM_2289	1.18	1.01E-02	5.54E-02	**1.94**	**3.33E-05**	**2.08E-04**	*nuoE*	NADH dehydrogenase I subunit E
EAM_2290	**1.40**	**2.75E-05**	**5.57E-04**	**2.04**	**1.95E-09**	**3.95E-08**	*nuoC*	NADH dehydrogenase I subunit C/D
EAM_2291	1.14	1.02E-02	5.56E-02	**1.99**	**1.26E-05**	**9.09E-05**	*nuoB*	NADH dehydrogenase I subunit B
EAM_2292	0.35	3.30E-01	6.28E-01	**1.27**	**5.62E-04**	**2.38E-03**	*nuoA*	NADH dehydrogenase I subunit A
EAM_2296	-0.42	4.48E-01	7.30E-01	**-1.53**	**7.58E-03**	**1.98E-02**	-	hypothetical protein
EAM_2297	-0.40	4.99E-01	7.69E-01	**-1.53**	**1.05E-02**	**2.54E-02**	-	hypothetical protein
EAM_2298	-0.30	4.02E-01	6.94E-01	**-1.30**	**2.83E-04**	**1.33E-03**	-	hypothetical protein
EAM_2306	-0.26	5.44E-01	7.99E-01	**-1.01**	**2.15E-02**	**4.32E-02**	-	ABC transporter substrate-binding protein
EAM_2308	-0.46	3.73E-01	6.70E-01	**-1.55**	**3.47E-03**	**1.06E-02**	-	ABC transporter permease
EAM_2310	-0.20	7.13E-01	8.83E-01	**-1.31**	**1.50E-02**	**3.31E-02**	-	hypothetical protein
EAM_2311	-0.73	3.15E-01	6.13E-01	**-1.92**	**9.94E-03**	**2.45E-02**	-	hypothetical protein
EAM_2315	0.35	2.55E-01	5.39E-01	**1.02**	**7.78E-04**	**3.12E-03**	*hisQ*	histidine ABC transporter permease
EAM_2321	0.62	3.15E-02	1.29E-01	**1.18**	**4.27E-05**	**2.59E-04**	*dedD*	membrane protein
EAM_2322	0.79	1.72E-02	8.30E-02	**1.59**	**2.49E-06**	**2.25E-05**	*folC*	bifunctional folylpolyglutamate synthase/dihydrofolate synthase
EAM_2323	0.63	5.66E-02	1.96E-01	**1.28**	**1.36E-04**	**7.27E-04**	*accD*	acetyl-coenzyme A carboxylase carboxyl transferase subunit beta
EAM_2324	-0.32	5.52E-01	8.03E-01	**-1.55**	**4.91E-03**	**1.40E-02**	-	hypothetical protein
EAM_2329	-0.56	1.60E-01	4.08E-01	**-1.45**	**3.29E-04**	**1.52E-03**	-	AraC family transcriptional regulator
EAM_2341	0.34	2.74E-01	5.64E-01	**1.01**	**1.12E-03**	**4.24E-03**	*mepA*	penicillin-insensitive murein endopeptidase
EAM_2343	0.64	1.25E-02	6.49E-02	**1.16**	**6.77E-06**	**5.36E-05**	-	methyltransferase

EAM_2344	-0.37	1.80E-01	4.39E-01	**-1.07**	**1.08E-04**	**5.90E-04**	-	hypothetical protein
EAM_2348	0.36	3.54E-01	6.53E-01	**1.58**	**7.68E-05**	**4.36E-04**	*fadL*	long-chain fatty acid transport protein
EAM_2363	0.51	5.58E-02	1.95E-01	**1.28**	**1.79E-06**	**1.72E-05**	-	mechanosensitive ion channel protein
EAM_2365	-0.47	2.62E-01	5.47E-01	**-1.42**	**1.10E-03**	**4.20E-03**	-	hypothetical protein
EAM_2367	-0.38	3.77E-01	6.73E-01	**-1.26**	**3.95E-03**	**1.17E-02**	-	hypothetical protein
EAM_2369	-0.78	1.21E-03	1.08E-02	**-1.55**	**2.52E-10**	**6.01E-09**	-	hypothetical protein
EAM_2370	**-1.07**	**4.76E-07**	**1.88E-05**	**-1.70**	**3.03E-15**	**2.78E-13**	-	universal stress protein
EAM_2375	-0.57	5.60E-02	1.95E-01	**-1.23**	**4.91E-05**	**2.94E-04**	*ipdC*	indole-3-pyruvate decarboxylase
EAM_2377	-0.36	2.88E-01	5.80E-01	**1.02**	**2.25E-03**	**7.47E-03**	-	hypothetical protein
EAM_2379	0.36	1.99E-01	4.64E-01	**1.32**	**3.00E-06**	**2.65E-05**	*nupC*	nucleoside permease
EAM_2390	0.48	2.51E-01	5.34E-01	**1.37**	**1.22E-03**	**4.57E-03**	*cysK*	cysteine synthase A
EAM_2391	0.56	1.32E-01	3.63E-01	**1.38**	**2.41E-04**	**1.16E-03**	*ptsH*	PTS system phosphocarrier protein
EAM_2394	-0.30	3.37E-01	6.37E-01	**-1.03**	**1.00E-03**	**3.87E-03**	-	two-component sensor kinase
EAM_2395	-0.60	3.64E-03	2.52E-02	**-1.08**	**2.29E-07**	**2.83E-06**	-	two-component response regulator
EAM_2411	**-4.50**	**5.08E-11**	**9.58E-09**	**-2.64**	**2.76E-05**	**1.77E-04**	-	hypothetical protein
EAM_2417	-0.04	9.01E-01	9.63E-01	**1.36**	**1.24E-06**	**1.25E-05**	-	hypothetical protein
EAM_2420	-0.49	5.46E-01	8.01E-01	**-1.96**	**2.01E-02**	**4.10E-02**	-	hypothetical protein
EAM_2421	-0.50	3.74E-01	6.70E-01	**-1.92**	**9.60E-04**	**3.74E-03**	-	non-ribosomal peptide synthetase
EAM_2422	0.07	8.52E-01	9.45E-01	**1.36**	**2.80E-04**	**1.32E-03**	-	hypothetical protein
EAM_2424	0.00	9.95E-01	1.00E+00	**1.14**	**1.23E-04**	**6.66E-04**	-	hypothetical protein
EAM_2427	-0.51	4.90E-01	7.61E-01	**-1.88**	**1.36E-02**	**3.10E-02**	-	hypothetical protein
EAM_2431	0.38	2.08E-01	4.77E-01	**1.10**	**3.72E-04**	**1.68E-03**	*nlpB*	lipoprotein
EAM_2442	0.84	9.36E-03	5.24E-02	**1.32**	**4.29E-05**	**2.61E-04**	*purN*	phosphoribosylglycinamide formyltransferase
EAM_2463	-0.43	3.65E-01	6.60E-01	**-1.46**	**2.30E-03**	**7.60E-03**	-	integrase (partial)
EAM_2467	**-1.43**	**2.63E-04**	**3.19E-03**	**-1.77**	**7.81E-06**	**6.04E-05**	*prt1*	extracellular metalloprotease
EAM_2468	**-1.53**	**2.09E-04**	**2.64E-03**	**-1.21**	**3.07E-03**	**9.57E-03**	-	hypothetical protein
EAM_2472	**1.04**	**1.86E-03**	**1.51E-02**	**1.59**	**2.55E-06**	**2.30E-05**	-	pyrrolo-quinoline quinone (PQQ) enzyme
EAM_2474	0.56	2.03E-02	9.37E-02	**1.23**	**4.56E-07**	**5.10E-06**	*hisS*	histidyl-tRNA synthetase
EAM_2475	0.50	1.14E-01	3.28E-01	**1.13**	**4.53E-04**	**1.98E-03**	*gcpE*	4-hydroxy-3-methylbut-2-en-1-yl diphosphate synthase
EAM_2476	0.37	8.95E-02	2.75E-01	**1.02**	**4.22E-06**	**3.52E-05**	-	DNA-binding protein
EAM_2478	0.87	3.30E-03	2.35E-02	**1.36**	**4.94E-06**	**4.01E-05**	-	hypothetical protein
EAM_2479	**1.25**	**3.30E-07**	**1.44E-05**	**1.60**	**8.32E-11**	**2.21E-09**	*ndk*	nucleoside diphosphate kinase

EAM_2486	-0.39	2.61E-01	5.46E-01	**-1.01**	**3.54E-03**	**1.07E-02**	-	proline racemase
EAM_2492	-0.44	1.44E-01	3.85E-01	**1.93**	**3.94E-10**	**9.10E-09**	*iscS*	cysteine desulfurase
EAM_2493	-0.96	1.67E-02	8.15E-02	**2.05**	**6.46E-07**	**7.02E-06**	-	transcriptional regulator
EAM_2494	0.55	4.84E-02	1.75E-01	**1.05**	**1.72E-04**	**8.90E-04**	-	tRNA/rRNA methyltransferase
EAM_2495	**1.21**	**2.07E-04**	**2.63E-03**	**1.95**	**3.75E-09**	**7.11E-08**	*suhB*	inositol monophosphatase
EAM_2503	0.80	3.58E-05	6.55E-04	**1.37**	**2.21E-12**	**9.53E-11**	-	transglycosylase
EAM_2508	0.10	8.48E-01	9.44E-01	**-1.39**	**9.47E-03**	**2.36E-02**	-	hypothetical protein
EAM_2509	-0.28	5.94E-01	8.32E-01	**-1.56**	**3.86E-03**	**1.15E-02**	-	pseudo
EAM_2516	0.53	4.81E-02	1.74E-01	**1.22**	**7.50E-06**	**5.88E-05**	*lepA*	GTP-binding protein
EAM_2518	-0.42	1.90E-01	4.53E-01	**-1.32**	**5.51E-05**	**3.24E-04**	-	phage antitermination Protein
EAM_2524	0.61	3.18E-02	1.30E-01	**1.39**	**1.42E-06**	**1.41E-05**	-	methyltransferase
EAM_2527	0.60	6.61E-02	2.21E-01	**1.04**	**1.44E-03**	**5.17E-03**	*grpE*	heat shock protein
EAM_2535	-0.50	4.48E-01	7.30E-01	**-1.78**	**8.63E-03**	**2.20E-02**	-	hypothetical protein
EAM_2536	-0.44	2.97E-01	5.91E-01	**-1.41**	**9.13E-04**	**3.58E-03**	*kpsC*	capsule polysaccharide export protein
EAM_2537	-0.45	4.40E-01	7.27E-01	**-1.45**	**1.35E-02**	**3.09E-02**	*cheW*	chemotaxis protein
EAM_2538	-0.27	5.84E-01	8.24E-01	**-1.28**	**9.08E-03**	**2.30E-02**	*cheA*	chemotaxis protein
EAM_2539	-0.32	5.79E-01	8.22E-01	**-1.39**	**1.67E-02**	**3.59E-02**	*motB*	motility protein B
EAM_2540	-0.40	4.70E-01	7.49E-01	**-1.75**	**2.31E-03**	**7.61E-03**	*motA*	motility protein A
EAM_2543	-0.46	4.59E-01	7.40E-01	**-1.50**	**1.73E-02**	**3.68E-02**	*fliA*	RNA polymerase sigma factor for flagellar operon
EAM_2545	-0.41	3.48E-01	6.48E-01	**-1.20**	**6.37E-03**	**1.72E-02**	*flgL*	flagellar hook-associated protein 3
EAM_2546	-0.21	6.28E-01	8.48E-01	**-1.02**	**2.07E-02**	**4.17E-02**	*flgK*	flagellar hook-associated protein 1
EAM_2547	-0.22	7.17E-01	8.86E-01	**-1.43**	**1.63E-02**	**3.52E-02**	*flgJ*	peptidoglycan hydrolase
EAM_2552	-0.20	7.08E-01	8.80E-01	**-1.44**	**7.46E-03**	**1.96E-02**	*flgE*	flagellar hook protein
EAM_2553	-0.55	3.90E-01	6.84E-01	**-1.46**	**2.42E-02**	**4.72E-02**	*flgD*	basal-body rod modification protein
EAM_2554	-0.42	5.24E-01	7.92E-01	**-1.89**	**5.69E-03**	**1.57E-02**	*flgC*	flagellar basal-body rod protein
EAM_2557	-0.46	3.41E-01	6.41E-01	**-1.57**	**1.56E-03**	**5.51E-03**	*flgM*	negative regulator of flagellin synthesis (anti-sigma-28 factor)
EAM_2558	-0.39	4.44E-01	7.29E-01	**-1.20**	**2.06E-02**	**4.17E-02**	*flgN*	flagella synthesis protein
EAM_2560	0.03	9.49E-01	9.82E-01	**-1.12**	**1.80E-02**	**3.78E-02**	*fliS*	flagellar protein
EAM_2562	-0.53	2.76E-01	5.66E-01	**-1.46**	**3.25E-03**	**1.00E-02**	-	flagellin
EAM_2566	-0.28	5.74E-01	8.16E-01	**-1.22**	**1.50E-02**	**3.31E-02**	-	aminotransferase
EAM_2567	-0.22	6.08E-01	8.40E-01	**-1.22**	**4.62E-03**	**1.33E-02**	-	transferase
EAM_2568	-0.40	5.63E-01	8.11E-01	**-1.74**	**1.30E-02**	**2.99E-02**	*fliR*	flagellar biosynthetic protein

EAM_2570	-0.34	5.54E-01	8.04E-01	**-1.45**	**1.34E-02**	**3.06E-02**	*fliP*	flagellar biosynthetic protein
EAM_2573	-0.24	6.17E-01	8.41E-01	**-1.11**	**2.08E-02**	**4.19E-02**	*fliM*	flagellar motor switch protein
EAM_2575	-0.32	5.69E-01	8.14E-01	**-1.47**	**1.00E-02**	**2.47E-02**	*fliK*	flagellar hook-length control protein
EAM_2576	-0.16	7.31E-01	8.90E-01	**-1.25**	**7.56E-03**	**1.98E-02**	*fliJ*	flagellar protein
EAM_2577	-0.28	5.64E-01	8.11E-01	**-1.22**	**1.39E-02**	**3.14E-02**	*fliI*	flagellum-specific ATP synthase
EAM_2578	-0.57	3.54E-01	6.53E-01	**-1.84**	**3.64E-03**	**1.10E-02**	*fliH*	flagellar assembly protein
EAM_2579	-0.26	6.11E-01	8.41E-01	**-1.31**	**1.07E-02**	**2.59E-02**	*fliG*	flagellar motor switch protein
EAM_2580	-0.34	5.38E-01	7.96E-01	**-1.54**	**5.79E-03**	**1.59E-02**	*fliF*	flagellar M-ring protein
EAM_2583	-0.16	7.54E-01	8.96E-01	**-1.51**	**2.76E-03**	**8.75E-03**	*cheB*	chemotaxis response regulator protein-glutamate methylesterase
EAM_2586	-0.39	4.34E-01	7.20E-01	**-1.40**	**5.69E-03**	**1.57E-02**	*flhB*	flagellar biosynthetic protein
EAM_2587	-0.46	2.77E-01	5.66E-01	**-1.31**	**2.26E-03**	**7.52E-03**	*flhA*	flagellar biosynthesis protein
EAM_2588	-0.37	4.79E-01	7.55E-01	**-1.81**	**7.63E-04**	**3.08E-03**	*flhE*	flagellar protein
EAM_2589	0.73	8.99E-04	8.33E-03	**1.71**	**2.34E-14**	**1.64E-12**	*tsx*	nucleoside-specific channel-forming protein
EAM_2594	-0.40	3.42E-01	6.42E-01	**-1.09**	**1.06E-02**	**2.55E-02**	*nrdH*	glutaredoxin-like protein
EAM_2600	0.94	4.56E-05	7.93E-04	**1.20**	**2.49E-07**	**3.04E-06**	*proX*	glycine betaine/L-proline ABC transporter substrate-binding protein
EAM_2601	-0.23	6.35E-01	8.51E-01	**-1.38**	**6.02E-03**	**1.64E-02**	-	major facilitator superfamily protein
EAM_2607	**-1.19**	**4.47E-05**	**7.85E-04**	**-1.97**	**4.50E-11**	**1.31E-09**	*trxC*	thioredoxin 2
EAM_2616	0.80	3.97E-03	2.69E-02	**1.33**	**1.87E-06**	**1.78E-05**	-	lipoprotein
EAM_2617	-0.82	7.06E-02	2.30E-01	**-2.58**	**6.63E-08**	**9.36E-07**	-	sigma(54) modulation protein
EAM_2620	0.57	8.03E-02	2.55E-01	**1.06**	**1.26E-03**	**4.67E-03**	*tyrA*	bifunctional chorismate mutase/prephenate dehydrogenase
EAM_2621	0.72	2.77E-02	1.19E-01	**1.58**	**1.89E-06**	**1.79E-05**	*aroF*	phospho-2-dehydro-3-deoxyheptonate aldolase
EAM_2622	-0.22	6.23E-01	8.45E-01	**-1.14**	**9.73E-03**	**2.42E-02**	-	pseudo
EAM_2625	-0.36	5.73E-01	8.16E-01	**-1.49**	**2.23E-02**	**4.43E-02**	-	pentatricopeptide repeat protein
EAM_2626	**2.18**	**1.35E-10**	**2.10E-08**	**3.36**	**4.29E-21**	**2.08E-18**	*rplS*	50S ribosomal protein L19
EAM_2627	**1.75**	**2.70E-06**	**8.49E-05**	**2.97**	**2.76E-14**	**1.87E-12**	*trmD*	tRNA (guanine-N(1)-)-methyltransferase
EAM_2628	**1.79**	**8.16E-06**	**2.07E-04**	**2.96**	**1.52E-12**	**7.09E-11**	*rimM*	16S_rRNA processing protein
EAM_2629	1.40	1.20E-02	6.34E-02	**2.92**	**6.59E-07**	**7.11E-06**	*rpsP*	30S ribosomal protein S16
EAM_2638	0.65	1.81E-02	8.63E-02	**1.30**	**3.22E-06**	**2.81E-05**	*alaS*	alanyl-tRNA synthetase
EAM_2646	**1.08**	**8.78E-04**	**8.15E-03**	**2.18**	**1.30E-11**	**4.19E-10**	-	hypothetical protein
EAM_2648	-0.20	6.52E-01	8.57E-01	**-1.08**	**1.59E-02**	**3.46E-02**	-	hypothetical protein
EAM_2655	-0.32	5.97E-01	8.35E-01	**-1.48**	**1.71E-02**	**3.65E-02**	-	type III secretion system protein

EAM_2656	-0.39	5.68E-01	8.14E-01	**-1.61**	**2.13E-02**	**4.28E-02**	-	type III secretion system protein
EAM_2657	-0.35	6.54E-01	8.57E-01	**-1.87**	**2.11E-02**	**4.23E-02**	-	type III secretion system protein
EAM_2658	-0.29	5.84E-01	8.24E-01	**-1.36**	**1.29E-02**	**2.98E-02**	-	type III secretion system protein
EAM_2660	-0.26	6.71E-01	8.64E-01	**-1.44**	**2.10E-02**	**4.23E-02**	-	type III secretion system protein
EAM_2661	-0.35	4.66E-01	7.45E-01	**-1.12**	**2.17E-02**	**4.33E-02**	-	type III secretion system protein
EAM_2662	-0.20	7.39E-01	8.94E-01	**-1.34**	**2.49E-02**	**4.81E-02**	-	type III secretion system protein
EAM_2665	-0.34	5.28E-01	7.94E-01	**-1.27**	**1.83E-02**	**3.81E-02**	-	type III secretion system protein
EAM_2669	-0.56	2.31E-01	5.08E-01	**-1.41**	**2.93E-03**	**9.22E-03**	-	type III secretion system protein
EAM_2674	-0.17	7.18E-01	8.86E-01	**-1.15**	**1.56E-02**	**3.41E-02**	-	type III secretion system protein
EAM_2676	-0.26	6.28E-01	8.48E-01	**-1.27**	**1.89E-02**	**3.91E-02**	-	type III secretion system protein
EAM_2685	0.06	8.63E-01	9.51E-01	**1.31**	**1.46E-04**	**7.75E-04**	*ispD*	4-diphosphocytidyl-2C-methyl-D-erythritol synthase
EAM_2696	0.64	3.98E-02	1.52E-01	**1.27**	**2.83E-05**	**1.80E-04**	-	6-pyruvoyl tetrahydrobiopterin synthase
EAM_2697	-0.71	6.36E-02	2.15E-01	**-1.62**	**3.03E-05**	**1.92E-04**	-	type III effector protein
EAM_2702	-0.31	4.62E-01	7.41E-01	**-1.29**	**2.73E-03**	**8.70E-03**	-	O-antigen acetylase
EAM_2703	**2.14**	**7.17E-08**	**4.16E-06**	**2.76**	**1.20E-11**	**4.01E-10**	*eno*	enolase
EAM_2704	**1.50**	**8.03E-06**	**2.06E-04**	**2.05**	**1.87E-09**	**3.82E-08**	*pyrG*	CTP synthetase
EAM_2714	0.87	6.63E-04	6.56E-03	**1.33**	**2.75E-07**	**3.29E-06**	*queF*	NADPH-dependent 7-cyano-7-deazaguanine reductase
EAM_2715	0.93	9.40E-05	1.43E-03	**1.13**	**2.28E-06**	**2.08E-05**	-	hypothetical protein
EAM_2719	0.46	3.41E-02	1.37E-01	**1.00**	**3.27E-06**	**2.83E-05**	*gcvA*	regulatory protein for glycine cleavage pathway
EAM_2727	**1.50**	**9.19E-06**	**2.29E-04**	**1.64**	**1.24E-06**	**1.25E-05**	-	membrane protein
EAM_2730	0.88	1.66E-04	2.22E-03	**1.10**	**2.57E-06**	**2.31E-05**	*ptrA*	protease 3
EAM_2733	-0.06	8.91E-01	9.61E-01	**-1.11**	**1.12E-02**	**2.68E-02**	-	hypothetical protein
EAM_2734	-0.33	6.44E-01	8.55E-01	**-1.72**	**1.98E-02**	**4.05E-02**	*ppdB*	prepilin peptidase-dependent protein B
EAM_2735	-0.52	3.48E-01	6.48E-01	**-1.44**	**9.85E-03**	**2.43E-02**	*ppdA*	prepilin peptidase-dependent protein A
EAM_2738	0.84	3.82E-05	6.88E-04	**1.08**	**1.50E-07**	**1.91E-06**	*lgt*	prolipoprotein diacylglyceryl transferase
EAM_2746	0.59	5.37E-03	3.36E-02	**1.00**	**2.63E-06**	**2.36E-05**	*aas*	bifunctional 2-acylglycerophosphoethanolamine acyltransferase/acyl [acyl-carrier-protein] synthetase
EAM_2756	-0.33	5.98E-01	8.35E-01	**-1.52**	**1.83E-02**	**3.81E-02**	-	hypothetical protein
EAM_2766	-0.54	4.48E-01	7.30E-01	**-1.67**	**2.32E-02**	**4.57E-02**	-	hypothetical protein
EAM_2775	**1.04**	**2.39E-03**	**1.86E-02**	**1.63**	**2.45E-06**	**2.22E-05**	*lysS*	lysyl-tRNA synthetase
EAM_2776	0.84	1.97E-02	9.18E-02	**1.84**	**5.40E-07**	**5.95E-06**	*prfB*	peptide chain release factor 2

EAM_2786	-0.31	1.76E-01	4.31E-01	**-1.15**	**8.78E-07**	**9.23E-06**	-	membrane protein
EAM_2811	**1.56**	**1.31E-09**	**1.40E-07**	**1.64**	**1.96E-10**	**4.77E-09**	*fbaA*	fructose-bisphosphate aldolase class 2
EAM_2812	0.83	4.08E-05	7.28E-04	**1.24**	**1.25E-09**	**2.64E-08**	*pgk*	phosphoglycerate kinase
EAM_2813	0.48	8.13E-02	2.57E-01	**1.01**	**2.38E-04**	**1.15E-03**	*epd*	D-erythrose 4-phosphate dehydrogenase
EAM_2814	1.00	1.38E-04	1.92E-03	**2.67**	**2.91E-22**	**1.74E-19**	*tktA*	transketolase
EAM_2815	0.77	1.84E-05	3.98E-04	**1.43**	**2.09E-15**	**2.08E-13**	-	peptidase
EAM_2823	0.13	6.66E-01	8.63E-01	**1.09**	**4.69E-04**	**2.04E-03**	*speB*	agmatinase
EAM_2826	**1.26**	**1.52E-04**	**2.06E-03**	**1.74**	**2.22E-07**	**2.75E-06**	*metK*	S-adenosylmethionine synthetase
EAM_2827	**1.12**	**1.02E-04**	**1.54E-03**	**1.95**	**2.87E-11**	**8.49E-10**	*galP*	galactose-proton symport
EAM_2836	-0.28	4.08E-01	7.01E-01	**-1.26**	**2.02E-04**	**1.01E-03**	-	RNA polymerase sigma factor
EAM_2838	-0.37	4.22E-01	7.11E-01	**-1.40**	**2.43E-03**	**7.88E-03**	-	type II/IV secretion system protein
EAM_2840	0.67	2.58E-02	1.13E-01	**1.39**	**4.79E-06**	**3.91E-05**	-	membrane protein
EAM_2841	0.47	9.87E-02	2.95E-01	**1.20**	**2.06E-05**	**1.37E-04**	-	Ham1 protein-like protein
EAM_2842	0.82	1.40E-04	1.94E-03	**1.09**	**4.14E-07**	**4.70E-06**	-	oxygen-independent coproporphyrinogen III oxidase
EAM_2843	**1.07**	**7.61E-07**	**2.70E-05**	**1.41**	**8.03E-11**	**2.15E-09**	-	hypothetical protein
EAM_2845	0.46	5.06E-02	1.81E-01	**1.05**	**7.65E-06**	**5.97E-05**	*trmB*	tRNA (guanine-N(7)-)-methyltransferase
EAM_2846	0.48	1.12E-02	5.97E-02	**1.31**	**6.94E-12**	**2.62E-10**	*mutY*	A/G-specific adenine glycosylase
EAM_2848	0.95	4.62E-07	1.84E-05	**1.59**	**6.12E-17**	**1.15E-14**	*mltC*	membrane-bound lytic murein transglycosylase C
EAM_2849	0.19	5.66E-01	8.12E-01	**1.21**	**2.27E-04**	**1.11E-03**	-	TetR family transcriptional regulator
EAM_2851	-0.28	6.37E-01	8.52E-01	**-1.73**	**3.85E-03**	**1.15E-02**	-	hypothetical protein
EAM_2852	-0.42	2.14E-01	4.85E-01	**-1.34**	**8.85E-05**	**4.94E-04**	-	integrase (partial)
EAM_2853	-0.42	5.39E-01	7.96E-01	**-1.72**	**1.53E-02**	**3.35E-02**	-	membrane protein
EAM_2854	-0.35	5.37E-01	7.96E-01	**-1.64**	**4.18E-03**	**1.22E-02**	-	hypothetical protein
EAM_2855	-0.23	7.13E-01	8.83E-01	**-1.72**	**7.39E-03**	**1.94E-02**	-	hypothetical protein
EAM_2856	-0.11	8.63E-01	9.51E-01	**-1.61**	**5.01E-03**	**1.42E-02**	-	hypothetical protein
EAM_2857	0.82	3.74E-04	4.22E-03	**1.13**	**1.15E-06**	**1.18E-05**	-	hypothetical protein
EAM_2867	-0.26	6.97E-01	8.74E-01	**-1.55**	**2.00E-02**	**4.07E-02**	-	hypothetical protein
EAM_2868	-0.30	5.45E-01	8.01E-01	**-1.18**	**1.98E-02**	**4.05E-02**	-	ATPase
EAM_2873	-0.42	4.26E-01	7.16E-01	**-1.43**	**7.54E-03**	**1.98E-02**	*hrpW*	pectate lyase
EAM_2874	-0.29	6.27E-01	8.48E-01	**-1.41**	**1.94E-02**	**3.99E-02**	-	type III secretion system protein
EAM_2877	-0.28	6.17E-01	8.41E-01	**-1.28**	**2.23E-02**	**4.42E-02**	*hrpN*	harpin
EAM_2879	-0.24	5.90E-01	8.30E-01	**-1.55**	**6.97E-04**	**2.86E-03**	*hrpT*	type III secretion system protein

EAM_2880	-0.25	6.06E-01	8.38E-01	**-1.27**	**9.10E-03**	**2.30E-02**	*hrcC*	type III secretion system protein
EAM_2881	-0.15	7.25E-01	8.89E-01	**-1.17**	**7.57E-03**	**1.98E-02**	*hrpG*	type III secretion system protein
EAM_2882	-0.40	3.65E-01	6.61E-01	**-1.65**	**2.78E-04**	**1.32E-03**	*hrpF*	type III secretion system protein
EAM_2883	-0.31	5.88E-01	8.28E-01	**-1.36**	**1.84E-02**	**3.83E-02**	*hrpE*	type III secretion system protein
EAM_2886	-0.51	1.81E-01	4.41E-01	**-1.05**	**6.23E-03**	**1.69E-02**	*hrpB*	type III secretion system protein
EAM_2887	**-1.40**	**2.75E-03**	**2.06E-02**	**-1.19**	**1.03E-02**	**2.53E-02**	*hrpA*	type III secretion system protein
EAM_2888	-0.32	6.16E-01	8.41E-01	**-1.69**	**1.03E-02**	**2.53E-02**	-	hypothetical protein
EAM_2889	-0.19	7.24E-01	8.89E-01	**-1.37**	**1.11E-02**	**2.65E-02**	-	pseudo
EAM_2896	-0.25	6.43E-01	8.53E-01	**-1.53**	**4.89E-03**	**1.39E-02**	*hrpI*	type III secretion system protein
EAM_2897	-0.30	6.24E-01	8.45E-01	**-1.57**	**1.05E-02**	**2.54E-02**	*hrpQ*	type III secretion system protein
EAM_2898	-0.07	8.78E-01	9.57E-01	**-1.22**	**5.42E-03**	**1.51E-02**	*hrcN*	type III secretion system protein
EAM_2900	-0.30	5.56E-01	8.05E-01	**-1.68**	**1.12E-03**	**4.24E-03**	*hrpP*	type III secretion system protein
EAM_2901	-0.31	4.96E-01	7.66E-01	**-1.16**	**1.16E-02**	**2.74E-02**	*hrcQ*	type III secretion system protein
EAM_2902	-0.26	6.82E-01	8.68E-01	**-1.64**	**1.19E-02**	**2.79E-02**	*hrcR*	type III secretion system protein
EAM_2903	-0.18	7.65E-01	9.03E-01	**-1.59**	**7.75E-03**	**2.01E-02**	*hrcS*	type III secretion system protein
EAM_2904	-0.34	5.03E-01	7.73E-01	**-1.25**	**1.50E-02**	**3.31E-02**	*hrcT*	type III secretion system protein
EAM_2905	-0.46	2.86E-01	5.76E-01	**-1.58**	**3.01E-04**	**1.40E-03**	*hrcU*	type III secretion system protein
EAM_2906	-0.17	7.43E-01	8.95E-01	**-1.22**	**1.81E-02**	**3.78E-02**	-	acetyltransferase
EAM_2913	0.97	2.48E-03	1.92E-02	**1.08**	**7.57E-04**	**3.06E-03**	*ivy*	inhibitor of lysozyme
EAM_2921	-0.27	6.48E-01	8.56E-01	**-1.56**	**1.06E-02**	**2.56E-02**	*mdtI*	multidrug resistance protein
EAM_2927	**1.06**	**1.96E-04**	**2.54E-03**	**1.33**	**3.19E-06**	**2.79E-05**	-	LuxR family transcriptional regulator
EAM_2931	**1.01**	**1.13E-03**	**1.01E-02**	**1.39**	**7.72E-06**	**6.01E-05**	*bglF*	PTS system transporter subunit IIC
EAM_2933	**1.25**	**3.44E-04**	**3.99E-03**	**1.50**	**1.92E-05**	**1.29E-04**	*bglY*	beta-glucosidase
EAM_2934	**1.05**	**5.45E-04**	**5.65E-03**	**1.14**	**1.74E-04**	**8.98E-04**	*bglA*	6-phospho-beta-glucosidase
EAM_2939	-0.44	4.47E-01	7.30E-01	**-1.51**	**1.05E-02**	**2.54E-02**	-	ABC transporter
EAM_2948	-0.22	3.51E-01	6.51E-01	**-1.06**	**8.58E-06**	**6.50E-05**	-	fimbrial protein
EAM_2959	**1.74**	**5.19E-07**	**2.00E-05**	**2.98**	**1.88E-16**	**2.80E-14**	*ddc*	L-2,4-diaminobutyrate decarboxylase
EAM_2960	**1.62**	**5.16E-06**	**1.50E-04**	**2.89**	**8.02E-15**	**6.24E-13**	*dat*	diaminobutyrate--2-oxoglutarate aminotransferase
EAM_2961	-0.34	3.56E-01	6.54E-01	**-1.04**	**5.28E-03**	**1.48E-02**	-	AraC family transcriptional regulator
EAM_2968	-0.07	8.81E-01	9.57E-01	**-1.18**	**1.51E-02**	**3.33E-02**	-	oxidoreductase (partial)
EAM_2969	-0.26	5.71E-01	8.15E-01	**-1.16**	**1.25E-02**	**2.91E-02**	-	hypothetical protein
EAM_2972	0.82	3.83E-03	2.61E-02	**1.64**	**1.15E-08**	**1.94E-07**	-	non-ribosomal peptide synthetase
EAM_2973	**1.93**	**3.77E-13**	**1.12E-10**	**2.19**	**2.36E-16**	**3.25E-14**	-	non-ribosomal peptide synthetase

EAM_2974	**1.83**	**3.86E-12**	**8.14E-10**	**2.06**	**7.41E-15**	**6.03E-13**	-	non-ribosomal peptide synthetase
EAM_2975	**1.49**	**4.92E-10**	**6.07E-08**	**1.42**	**2.84E-09**	**5.61E-08**	-	non-ribosomal peptide synthetase
EAM_2976	**2.05**	**2.09E-15**	**1.27E-12**	**1.88**	**2.62E-13**	**1.40E-11**	-	non-ribosomal peptide synthetase
EAM_2977	**2.36**	**1.47E-12**	**3.30E-10**	**2.29**	**5.69E-12**	**2.19E-10**	-	non-ribosomal peptide synthetase
EAM_2978	**1.46**	**5.26E-09**	**4.60E-07**	**1.39**	**2.54E-08**	**3.99E-07**	-	hypothetical protein
EAM_2980	**1.43**	**2.37E-11**	**4.72E-09**	**1.32**	**5.47E-10**	**1.23E-08**	-	amidohydrolase
EAM_2988	**1.30**	**1.04E-06**	**3.59E-05**	**1.18**	**9.13E-06**	**6.83E-05**	*tolC*	outer membrane protein
EAM_2991	-0.43	1.62E-01	4.10E-01	**-1.15**	**2.14E-04**	**1.05E-03**	-	aromatic ring-opening dioxygenase
EAM_3003	0.56	1.92E-01	4.55E-01	**2.66**	**5.59E-09**	**1.01E-07**	*rpsU*	30S ribosomal protein S21
EAM_3008	-0.17	7.98E-01	9.20E-01	**-1.67**	**1.25E-02**	**2.90E-02**	-	hypothetical protein
EAM_3015	-0.15	6.96E-01	8.74E-01	**-1.29**	**9.03E-04**	**3.56E-03**	-	transposase (partial)
EAM_3016	-0.09	9.03E-01	9.64E-01	**-1.62**	**1.99E-02**	**4.06E-02**	-	hypothetical protein
EAM_3020	-0.06	9.33E-01	9.79E-01	**-1.66**	**9.84E-03**	**2.43E-02**	-	hypothetical protein
EAM_3023	-0.27	6.19E-01	8.41E-01	**-1.28**	**1.80E-02**	**3.77E-02**	-	pseudo
EAM_3028	0.60	1.12E-02	5.99E-02	**1.85**	**1.42E-14**	**1.08E-12**	-	plasmid-like protein
EAM_3046	0.61	2.73E-02	1.18E-01	**1.21**	**1.22E-05**	**8.83E-05**	*valS*	valyl-tRNA synthetase
EAM_3047	0.22	5.18E-01	7.87E-01	**1.21**	**3.50E-04**	**1.60E-03**	-	AraC family transcriptional regulator
EAM_3049	-0.02	9.68E-01	9.90E-01	**-1.32**	**4.77E-03**	**1.36E-02**	-	phosphatase
EAM_3055	**1.90**	**1.99E-06**	**6.43E-05**	**2.09**	**1.93E-07**	**2.43E-06**	*pyrI*	aspartate carbamoyltransferase
EAM_3056	0.90	9.39E-03	5.24E-02	**1.89**	**9.77E-08**	**1.30E-06**	-	endoribonuclease
EAM_3059	**1.46**	**2.36E-05**	**4.93E-04**	**-1.24**	**3.46E-04**	**1.59E-03**	*nrdD*	anaerobic ribonucleoside-triphosphate reductase
EAM_3060	0.61	1.52E-01	3.96E-01	**-1.15**	**8.54E-03**	**2.18E-02**	*nrdG*	anaerobic ribonucleoside-triphosphate reductase activating protein
EAM_3067	**1.75**	**5.24E-06**	**1.51E-04**	**2.15**	**3.35E-08**	**5.15E-07**	*pnp*	polynucleotide phosphorylase
EAM_3068	**1.74**	**2.98E-04**	**3.52E-03**	**3.13**	**6.21E-10**	**1.37E-08**	*rpsO*	30S ribosomal protein S15
EAM_3069	0.59	1.05E-02	5.68E-02	**1.17**	**5.27E-07**	**5.86E-06**	*truB*	tRNA pseudouridine synthase B
EAM_3071	**1.36**	**6.02E-06**	**1.70E-04**	**1.79**	**3.73E-09**	**7.11E-08**	*infB*	translation initiation factor IF-2
EAM_3072	**1.41**	**7.11E-08**	**4.16E-06**	**2.16**	**6.83E-16**	**8.43E-14**	*nusA*	transcription elongation protein
EAM_3073	**1.66**	**7.69E-08**	**4.24E-06**	**2.54**	**1.26E-15**	**1.36E-13**	-	hypothetical protein
EAM_3074	**1.42**	**3.67E-04**	**4.18E-03**	**2.24**	**4.23E-08**	**6.40E-07**	*secG*	protein-export protein
EAM_3078	0.83	1.59E-02	7.82E-02	**1.25**	**2.93E-04**	**1.37E-03**	*ftsH*	cell division protein
EAM_3087	1.17	1.11E-02	5.96E-02	**2.36**	**8.46E-07**	**8.91E-06**	*rpmA*	50S ribosomal protein L27
EAM_3088	**1.35**	**2.14E-03**	**1.69E-02**	**2.16**	**1.65E-06**	**1.61E-05**	*rplU*	50S ribosomal protein L21

EAM_3093	0.86	4.66E-03	2.99E-02	**1.33**	**1.51E-05**	**1.05E-04**	-	hypothetical protein
EAM_3094	0.76	3.10E-02	1.28E-01	**1.14**	**1.19E-03**	**4.45E-03**	-	hypothetical protein
EAM_3100	0.79	3.87E-03	2.63E-02	**1.27**	**4.78E-06**	**3.91E-05**	*kdsC*	3-deoxy-D-manno-octulosonate 8-phosphate phosphatase
EAM_3102	0.85	7.00E-04	6.80E-03	**1.33**	**1.26E-07**	**1.64E-06**	-	hypothetical protein
EAM_3115	**1.22**	**5.47E-03**	**3.40E-02**	**2.82**	**1.15E-09**	**2.45E-08**	*rpsI*	30S ribosomal protein S9
EAM_3116	**1.46**	**2.95E-03**	**2.18E-02**	**2.63**	**2.46E-07**	**3.01E-06**	*rplM*	50S ribosomal protein L13
EAM_3121	0.80	8.46E-03	4.81E-02	**1.43**	**3.74E-06**	**3.20E-05**	*mdh*	malate dehydrogenase
EAM_3132	0.48	7.13E-02	2.33E-01	**1.27**	**2.07E-06**	**1.94E-05**	-	inhibitor of septum formation
EAM_3134	0.45	3.71E-02	1.45E-01	**1.17**	**8.62E-08**	**1.17E-06**	*mreC*	rod shape-determining protein
EAM_3135	0.51	1.03E-01	3.06E-01	**1.45**	**5.37E-06**	**4.33E-05**	*mreB*	rod shape-determining protein
EAM_3140	**1.85**	**2.93E-09**	**2.92E-07**	**2.91**	**2.60E-19**	**7.77E-17**	*aroQ*	3-dehydroquinate dehydratase
EAM_3141	**2.34**	**1.42E-10**	**2.13E-08**	**3.24**	**7.81E-18**	**2.15E-15**	*accB*	biotin carboxyl carrier protein of acetyl-CoA carboxylase
EAM_3142	**2.33**	**7.12E-13**	**1.70E-10**	**3.15**	**5.79E-21**	**2.30E-18**	*accC*	biotin carboxylase
EAM_3145	0.78	1.38E-03	1.20E-02	**1.44**	**4.94E-09**	**9.08E-08**	*prmA*	50S ribosomal protein L11 methyltransferase
EAM_3146	**1.53**	**1.03E-04**	**1.54E-03**	**2.67**	**6.98E-11**	**1.94E-09**	*dusB*	tRNA-dihydrouridine synthase B
EAM_3147	**1.79**	**2.42E-06**	**7.66E-05**	**3.41**	**2.97E-17**	**6.65E-15**	*fis*	DNA-binding protein
EAM_3149	-0.19	7.51E-01	8.96E-01	**-1.70**	**3.29E-03**	**1.01E-02**	-	hypothetical protein
EAM_3151	-0.04	9.26E-01	9.74E-01	**-1.24**	**2.84E-03**	**8.99E-03**	-	ABC transporter substrate-binding protein
EAM_3153	-0.15	7.26E-01	8.89E-01	**-1.15**	**8.06E-03**	**2.08E-02**	-	ABC transporter permease
EAM_3168	0.06	8.79E-01	9.57E-01	**-1.01**	**1.08E-02**	**2.60E-02**	-	hypothetical protein
EAM_3172	**1.90**	**2.79E-05**	**5.62E-04**	**3.28**	**7.21E-12**	**2.69E-10**	*rplQ*	50S ribosomal protein L17
EAM_3173	**1.71**	**2.10E-04**	**2.65E-03**	**3.02**	**4.28E-10**	**9.78E-09**	*rpoA*	DNA-directed RNA polymerase subunit alpha
EAM_3174	**1.75**	**6.59E-06**	**1.79E-04**	**2.58**	**1.09E-10**	**2.81E-09**	*rpsD*	30S ribosomal protein S4
EAM_3175	**1.73**	**4.11E-04**	**4.55E-03**	**3.03**	**3.38E-09**	**6.50E-08**	*rpsK*	30S ribosomal protein S11
EAM_3176	**1.30**	**2.33E-03**	**1.82E-02**	**2.57**	**7.76E-09**	**1.36E-07**	*rpsM*	30S ribosomal protein S13
EAM_3177	1.19	6.68E-02	2.22E-01	**2.37**	**4.22E-04**	**1.87E-03**	*rpmJ*	50S ribosomal protein L36
EAM_3178	**1.36**	**6.51E-03**	**3.92E-02**	**2.33**	**6.63E-06**	**5.27E-05**	*secY*	preprotein translocase subunit SecY
EAM_3179	**1.93**	**1.66E-04**	**2.22E-03**	**2.96**	**2.70E-08**	**4.19E-07**	*rplO*	50S ribosomal protein L15
EAM_3180	**2.13**	**9.37E-05**	**1.43E-03**	**3.45**	**1.94E-09**	**3.95E-08**	*rpmD*	50S ribosomal protein L30
EAM_3181	**1.97**	**2.92E-05**	**5.85E-04**	**2.89**	**3.26E-09**	**6.31E-08**	*rpsE*	30S ribosomal protein S5
EAM_3182	**2.36**	**2.05E-07**	**1.03E-05**	**3.07**	**4.76E-11**	**1.37E-09**	*rplR*	50S ribosomal protein L18

EAM_3183	**2.38**	**8.83E-05**	**1.37E-03**	**3.25**	**2.32E-07**	**2.86E-06**	*rplF*	50S ribosomal protein L6
EAM_3184	**2.35**	**2.42E-08**	**1.73E-06**	**3.14**	**5.59E-13**	**2.86E-11**	*rpsH*	30S ribosomal protein S8
EAM_3185	**2.20**	**1.71E-09**	**1.75E-07**	**3.04**	**8.84E-16**	**1.02E-13**	*rpsN*	30S ribosomal protein S14
EAM_3186	**1.97**	**6.88E-05**	**1.10E-03**	**3.18**	**8.57E-10**	**1.84E-08**	*rplE*	50S ribosomal protein L5
EAM_3187	**2.03**	**5.88E-05**	**9.85E-04**	**2.77**	**9.68E-08**	**1.29E-06**	*rplX*	50S ribosomal protein L24
EAM_3188	**1.52**	**7.61E-04**	**7.31E-03**	**2.16**	**2.63E-06**	**2.36E-05**	*rplN*	50S ribosomal protein L14
EAM_3189	**2.29**	**1.17E-05**	**2.73E-04**	**3.38**	**5.87E-10**	**1.31E-08**	*rpsQ*	30S ribosomal protein S17
EAM_3190	**2.08**	**4.54E-04**	**4.93E-03**	**2.99**	**1.11E-06**	**1.14E-05**	*rpmC*	50S ribosomal protein L29
EAM_3191	**2.09**	**3.33E-05**	**6.35E-04**	**3.26**	**6.84E-10**	**1.48E-08**	*rplP*	50S ribosomal protein L16
EAM_3192	**2.25**	**1.61E-06**	**5.30E-05**	**3.35**	**9.25E-12**	**3.25E-10**	*rpsC*	30S ribosomal protein S3
EAM_3193	**2.07**	**6.97E-08**	**4.16E-06**	**3.21**	**1.40E-15**	**1.48E-13**	*rplV*	50S ribosomal protein L22
EAM_3194	**2.70**	**6.13E-11**	**1.05E-08**	**4.09**	**1.04E-20**	**3.74E-18**	*rpsS*	30S ribosomal protein S19
EAM_3195	**2.34**	**6.39E-06**	**1.77E-04**	**3.74**	**1.10E-11**	**3.78E-10**	*rplB*	50S ribosomal protein L2
EAM_3196	**2.48**	**1.04E-07**	**5.59E-06**	**3.75**	**2.88E-14**	**1.91E-12**	*rplW*	50S ribosomal protein L23
EAM_3197	**2.45**	**7.09E-06**	**1.90E-04**	**4.04**	**4.75E-12**	**1.89E-10**	*rplD*	50S ribosomal protein L4
EAM_3198	**2.40**	**5.07E-07**	**1.98E-05**	**3.76**	**1.01E-13**	**6.11E-12**	*rplC*	50S ribosomal protein L3
EAM_3199	**2.44**	**5.98E-07**	**2.21E-05**	**4.27**	**7.21E-16**	**8.61E-14**	*rpsJ*	30S ribosomal protein S10
EAM_3200	0.63	8.92E-02	2.75E-01	**1.21**	**1.23E-03**	**4.60E-03**	*tufA*	elongation factor TU
EAM_3201	**1.05**	**7.21E-03**	**4.25E-02**	**1.85**	**3.95E-06**	**3.34E-05**	*fusA*	elongation factor G
EAM_3202	1.20	1.25E-02	6.49E-02	**2.54**	**3.59E-07**	**4.14E-06**	*rpsG*	30S ribosomal protein S7
EAM_3203	0.83	1.52E-01	3.96E-01	**2.63**	**1.55E-05**	**1.08E-04**	*rpsL*	30S ribosomal protein S12
EAM_3204	0.43	6.41E-02	2.16E-01	**1.22**	**1.08E-07**	**1.42E-06**	-	DsrH-like sulfur reduction protein
EAM_3207	0.67	3.27E-02	1.33E-01	**1.12**	**3.92E-04**	**1.75E-03**	-	hypothetical protein
EAM_3208	**1.04**	**4.52E-04**	**4.93E-03**	**1.37**	**4.93E-06**	**4.01E-05**	*fkpA*	FKBP-type peptidylprolyl isomerase
EAM_3214	0.56	9.44E-03	5.25E-02	**1.05**	**1.55E-06**	**1.53E-05**	-	ABC transporter ATP-binding protein
EAM_3219	0.32	3.42E-01	6.42E-01	**1.20**	**3.57E-04**	**1.62E-03**	*tauA*	taurine ABC transporter substrate-binding protein
EAM_3225	1.00	9.81E-07	3.44E-05	**1.74**	**4.36E-17**	**9.19E-15**	*crp*	catabolite gene activator
EAM_3227	0.30	1.98E-01	4.62E-01	**1.37**	**7.32E-09**	**1.30E-07**	*argD*	acetylornithine/succinyldiaminopimelate aminotransferase
EAM_3232	-0.64	1.12E-01	3.23E-01	**-1.29**	**1.56E-03**	**5.51E-03**	-	hypothetical protein
EAM_3234	**1.33**	**7.85E-04**	**7.48E-03**	**1.77**	**9.64E-06**	**7.18E-05**	*trpS*	tryptophanyl-tRNA synthetase
EAM_3235	0.72	6.65E-02	2.21E-01	**1.16**	**3.12E-03**	**9.68E-03**	*gph*	phosphoglycolate phosphatase
EAM_3236	**1.12**	**3.59E-06**	**1.05E-04**	**1.27**	**1.80E-07**	**2.27E-06**	*rpe*	ribulose-phosphate 3-epimerase

EAM_3239	**1.06**	**1.67E-03**	**1.40E-02**	**2.00**	**7.86E-09**	**1.37E-07**	*aroB*	3-dehydroquinate synthase
EAM_3240	**1.14**	**1.06E-04**	**1.57E-03**	**2.34**	**1.49E-14**	**1.11E-12**	*aroK*	shikimate kinase
EAM_3246	0.77	2.03E-03	1.62E-02	**1.27**	**3.74E-07**	**4.30E-06**	*mrcA*	penicillin-binding protein 1A
EAM_3251	**1.92**	**2.33E-07**	**1.13E-05**	**2.90**	**5.21E-14**	**3.33E-12**	*pckA*	phosphoenolpyruvate carboxykinase
EAM_3258	-0.85	3.58E-03	2.49E-02	**-1.06**	**2.87E-04**	**1.35E-03**	*gntX*	competence protein
EAM_3264	0.57	8.84E-02	2.73E-01	**1.17**	**4.45E-04**	**1.96E-03**	*glpE*	thiosulfate sulfurtransferase
EAM_3266	-0.55	1.72E-01	4.25E-01	**-1.47**	**3.54E-04**	**1.61E-03**	-	hypothetical protein
EAM_3268	-0.33	2.51E-01	5.35E-01	**-1.04**	**2.91E-04**	**1.37E-03**	*glgP*	glycogen phosphorylase
EAM_3269	-0.33	3.32E-01	6.31E-01	**-1.07**	**1.98E-03**	**6.73E-03**	*glgA*	glycogen synthase
EAM_3270	-0.27	5.13E-01	7.83E-01	**-1.25**	**3.20E-03**	**9.90E-03**	*glgC*	glucose-1-phosphate adenylyltransferase
EAM_3271	-0.35	3.66E-01	6.61E-01	**-1.32**	**7.89E-04**	**3.16E-03**	*glgX*	glycogen debranching protein
EAM_3272	-0.28	5.26E-01	7.92E-01	**-1.54**	**6.56E-04**	**2.72E-03**	*glgB*	1,4-alpha-glucan branching protein
EAM_3273	0.51	1.87E-02	8.76E-02	**1.05**	**1.18E-06**	**1.19E-05**	*asd*	aspartate-semialdehyde dehydrogenase
EAM_3275	0.72	8.81E-02	2.73E-01	**1.45**	**6.86E-04**	**2.82E-03**	*gntK*	thermoresistant gluconokinase
EAM_3277	-0.15	6.63E-01	8.61E-01	**-1.38**	**5.46E-05**	**3.23E-04**	-	hypothetical protein
EAM_3278	-0.03	9.46E-01	9.82E-01	**-1.15**	**9.20E-03**	**2.31E-02**	-	acetyltransferase
EAM_3280	**-1.31**	**1.82E-06**	**5.92E-05**	**-1.22**	**7.23E-06**	**5.69E-05**	-	hypothetical protein
EAM_3282	-0.24	6.09E-01	8.40E-01	**-1.47**	**2.17E-03**	**7.25E-03**	*ugpC*	sn-Glycerol-3-phosphate ABC transporter ATP-binding protein
EAM_3285	-0.29	5.53E-01	8.03E-01	**-1.58**	**1.90E-03**	**6.49E-03**	*ugpB*	glycerol-3-phosphate ABC transporter substrate-binding protein
EAM_3292	0.80	2.25E-02	1.02E-01	**1.33**	**1.77E-04**	**9.06E-04**	*rpoH*	RNA polymerase sigma-32 factor
EAM_3298	0.63	3.66E-02	1.44E-01	**1.00**	**9.17E-04**	**3.60E-03**	-	membrane protein
EAM_3299	0.92	3.69E-04	4.19E-03	**1.55**	**2.85E-09**	**5.61E-08**	*zntA*	lead, cadmium, zinc and mercury-transporting ATPase
EAM_3300	-0.16	6.69E-01	8.64E-01	**1.11**	**1.98E-03**	**6.73E-03**	*tusA*	sulfurtransferase tRNA 2-thiouridine synthesizin protein A
EAM_3301	0.45	4.48E-02	1.65E-01	**1.45**	**1.02E-10**	**2.68E-09**	-	membrane protein
EAM_3302	0.82	5.83E-04	5.98E-03	**1.28**	**8.70E-08**	**1.18E-06**	*dcrB*	bacteriophage adsorption lipoprotein
EAM_3309	-0.15	7.08E-01	8.81E-01	**-1.07**	**9.74E-03**	**2.42E-02**	-	aldehyde dehydrogenase
EAM_3310	-0.16	7.53E-01	8.96E-01	**-1.21**	**1.72E-02**	**3.66E-02**	-	membrane protein
EAM_3312	0.69	5.62E-03	3.48E-02	**1.08**	**1.85E-05**	**1.25E-04**	*proP*	proline/betaine transporter
EAM_3314	0.18	7.27E-01	8.89E-01	**-1.38**	**1.02E-02**	**2.49E-02**	-	LuxR family transcriptional regulator
EAM_3319	-0.35	4.95E-01	7.65E-01	**-1.50**	**3.69E-03**	**1.11E-02**	-	ornithine cyclodeaminase

EAM_3320	-0.18	7.52E-01	8.96E-01	**-1.30**	**2.03E-02**	**4.12E-02**	-	AraC family transcriptional regulator
EAM_3323	**1.25**	**3.14E-05**	**6.16E-04**	**1.95**	**2.08E-10**	**4.99E-09**	*pitA*	low-affinity inorganic phosphate transporter
EAM_3324	-0.69	4.06E-03	2.72E-02	**-1.39**	**1.20E-08**	**2.02E-07**	*uspB*	universal stress protein B
EAM_3327	**1.62**	**3.09E-05**	**6.12E-04**	**1.17**	**2.35E-03**	**7.71E-03**	*prlC*	oligopeptidase A
EAM_3328	0.36	1.31E-01	3.62E-01	**1.09**	**5.64E-06**	**4.52E-05**	-	membrane-associated protease
EAM_3335	0.06	9.07E-01	9.65E-01	**-1.50**	**5.16E-03**	**1.45E-02**	-	hypothetical protein
EAM_3336	-0.11	8.56E-01	9.47E-01	**-1.50**	**2.50E-03**	**8.05E-03**	-	transposase (partial)
EAM_3339	-0.05	9.04E-01	9.64E-01	**-1.04**	**1.71E-02**	**3.65E-02**	-	FAD-binding oxidoreductase
EAM_3340	0.33	4.42E-01	7.27E-01	**-1.14**	**9.36E-03**	**2.34E-02**	-	(2Fe-2S)-binding protein
EAM_3342	-0.24	6.14E-01	8.41E-01	**-1.19**	**1.19E-02**	**2.79E-02**	-	exported polysaccharide acetylesterase
EAM_3353	-0.18	7.07E-01	8.80E-01	**-1.40**	**3.20E-03**	**9.89E-03**	-	methyl-accepting chemotaxis protein
EAM_3371	0.13	7.76E-01	9.11E-01	**1.16**	**1.26E-02**	**2.92E-02**	-	membrane protein
EAM_3399	-0.63	2.38E-02	1.07E-01	**-1.26**	**8.16E-06**	**6.25E-05**	-	hypothetical protein
EAM_3407	-0.25	4.85E-01	7.59E-01	**-1.19**	**1.25E-03**	**4.66E-03**	-	pseudo
EAM_3416	0.60	2.46E-02	1.09E-01	**1.09**	**5.25E-05**	**3.13E-04**	*mtlA*	PTS system mannitol-specific transporter subunit IICBA
EAM_3426	-0.54	2.34E-01	5.11E-01	**-1.05**	**2.27E-02**	**4.48E-02**	-	hypothetical protein
EAM_3438	-0.31	1.60E-01	4.08E-01	**-1.11**	**9.63E-07**	**1.01E-05**	-	sugar isomerase
EAM_3443	-0.07	9.02E-01	9.63E-01	**-1.12**	**1.99E-02**	**4.06E-02**	-	hypothetical protein
EAM_3444	**1.80**	**1.21E-08**	**9.20E-07**	**2.63**	**5.97E-16**	**7.92E-14**	-	hypothetical protein
EAM_3452	0.70	1.26E-03	1.11E-02	**1.12**	**2.79E-07**	**3.32E-06**	*dnaN*	DNA polymerase III subunit beta
EAM_3454	1.10	3.83E-02	1.48E-01	**3.09**	**5.28E-08**	**7.78E-07**	*rpmH*	50S ribosomal protein L34
EAM_3455	**2.02**	**9.28E-08**	**5.04E-06**	**3.00**	**1.97E-14**	**1.41E-12**	*rnpA*	ribonuclease P protein component
EAM_3456	**1.85**	**2.51E-08**	**1.73E-06**	**2.88**	**4.93E-17**	**9.81E-15**	-	hypothetical protein
EAM_3457	**2.65**	**7.10E-14**	**2.83E-11**	**3.45**	**4.64E-21**	**2.08E-18**	*oxaA*	hypothetical protein
EAM_3466	**-1.31**	**1.47E-04**	**2.02E-03**	**-1.03**	**2.65E-03**	**8.45E-03**	*pstC*	phosphate ABC transporter permease
EAM_3467	**-1.33**	**3.14E-05**	**6.16E-04**	**-1.13**	**3.66E-04**	**1.66E-03**	*pstS*	phosphate ABC transporter substrate-binding protein
EAM_3469	-0.06	9.09E-01	9.65E-01	**-1.39**	**1.45E-02**	**3.25E-02**	-	phage holin family protein
EAM_3473	**2.12**	**5.97E-07**	**2.21E-05**	**2.97**	**1.27E-11**	**4.14E-10**	*atpC*	ATP synthase subunit epsilon
EAM_3474	**2.18**	**3.25E-07**	**1.44E-05**	**3.13**	**1.72E-12**	**7.81E-11**	*atpD*	ATP synthase subunit beta
EAM_3475	**2.55**	**5.03E-13**	**1.39E-10**	**3.60**	**1.73E-22**	**1.43E-19**	*atpG*	ATP synthase subunit gamma
EAM_3476	**2.58**	**3.45E-09**	**3.25E-07**	**3.86**	**6.43E-17**	**1.15E-14**	*atpA*	ATP synthase subunit alpha

EAM_3477	**1.92**	**7.63E-08**	**4.24E-06**	**2.94**	**2.42E-15**	**2.34E-13**	*atpH*	ATP synthase subunit delta
EAM_3478	**2.71**	**6.00E-19**	**2.15E-15**	**3.56**	**2.17E-29**	**7.78E-26**	*atpF*	ATP synthase subunit B
EAM_3479	**2.17**	**8.44E-06**	**2.13E-04**	**3.52**	**7.83E-12**	**2.83E-10**	*atpE*	ATP synthase subunit C
EAM_3480	**2.25**	**2.23E-13**	**7.27E-11**	**3.13**	**5.44E-23**	**6.50E-20**	*atpB*	ATP synthase subunit A
EAM_3481	**1.70**	**1.44E-05**	**3.28E-04**	**3.14**	**3.02E-14**	**1.97E-12**	*atpI*	ATP synthase I
EAM_3482	0.52	3.04E-02	1.26E-01	**1.01**	**2.73E-05**	**1.75E-04**	*gidB*	glucose-inhibited division protein B
EAM_3483	0.75	6.98E-04	6.79E-03	**1.25**	**1.86E-08**	**2.97E-07**	*gidA*	tRNA uridine 5-carboxymethylaminomethyl modification enzyme
EAM_0029	**1.79**	**1.66E-05**	**3.64E-04**	-0.53	1.87E-01	2.49E-01	*hemN*	oxygen-independent coproporphyrinogen III oxidase
EAM_0061	**1.22**	**6.80E-08**	**4.16E-06**	0.55	1.34E-02	3.06E-02	-	restriction enzyme
EAM_0101	**-1.71**	**1.08E-04**	**1.59E-03**	-0.60	1.68E-01	2.26E-01	*cpxP*	extracytoplasmic stress protein
EAM_0122	**1.10**	**2.77E-04**	**3.32E-03**	0.57	5.88E-02	9.44E-02	*hslU*	ATP-dependent Hsl protease ATP-binding subunit
EAM_0246	**1.07**	**1.85E-03**	**1.51E-02**	0.80	1.91E-02	3.93E-02	*hupA*	histone like DNA-binding protein HU-alpha
EAM_0402	**1.69**	**5.90E-15**	**3.02E-12**	0.25	2.35E-01	3.00E-01	-	hypothetical protein
EAM_0403	**1.37**	**6.78E-07**	**2.45E-05**	0.06	8.18E-01	8.62E-01	-	methyltransferase
EAM_0488	**2.04**	**3.09E-03**	**2.26E-02**	-0.47	4.86E-01	5.60E-01	-	hypothetical protein
EAM_0490	**-1.02**	**1.99E-04**	**2.56E-03**	-0.97	3.67E-04	1.66E-03	-	membrane protein
EAM_0510	**-1.35**	**8.00E-04**	**7.57E-03**	-0.65	1.02E-01	1.48E-01	-	carbonic anhydrase
EAM_0511	**-1.26**	**1.28E-04**	**1.82E-03**	-0.68	3.50E-02	6.31E-02	-	sulfate transporter
EAM_0583	**1.44**	**3.53E-09**	**3.25E-07**	0.95	8.78E-05	4.91E-04	-	hypothetical protein
EAM_0586	**1.02**	**1.73E-05**	**3.75E-04**	0.97	3.72E-05	2.29E-04	-	protein phosphatase
EAM_0593	**1.18**	**8.06E-06**	**2.06E-04**	0.97	2.18E-04	1.07E-03	-	protein kinase
EAM_0611	**1.20**	**1.56E-04**	**2.10E-03**	0.92	3.58E-03	1.08E-02	*osmY*	osmotically inducible protein Y
EAM_0700	**1.15**	**1.79E-04**	**2.36E-03**	0.69	2.28E-02	4.50E-02	*ddlB*	D-alanine--D-alanine ligase
EAM_0742	**-1.04**	**3.08E-05**	**6.12E-04**	-0.86	5.62E-04	2.38E-03	-	outer membrane protease
EAM_0890	**-1.36**	**5.81E-05**	**9.82E-04**	-0.65	5.25E-02	8.61E-02	*fadE*	acyl-CoA dehydrogenase
EAM_1005	**1.20**	**7.82E-03**	**4.55E-02**	0.89	4.62E-02	7.85E-02	*amtB*	ammonia transporter
EAM_1064	**2.68**	**5.59E-07**	**2.11E-05**	1.10	3.02E-02	5.61E-02	*adhP*	alcohol dehydrogenase
EAM_1116	**-1.89**	**3.12E-03**	**2.28E-02**	-0.39	5.30E-01	6.00E-01	-	cold shock-like protein
EAM_1200	**-1.77**	**3.58E-05**	**6.55E-04**	-0.34	4.12E-01	4.86E-01	-	membrane protein
EAM_1225	**-1.03**	**1.38E-03**	**1.20E-02**	-0.58	7.15E-02	1.11E-01	-	membrane protein
EAM_1366	**1.66**	**9.44E-09**	**7.52E-07**	0.89	1.68E-03	5.83E-03	*pyrD*	dihydroorotate dehydrogenase

EAM_1408	**-1.18**	**1.99E-03**	**1.60E-02**	-0.71	6.07E-02	9.71E-02	-	MarR family transcriptional regulator
EAM_1532	**-1.00**	**2.23E-05**	**4.72E-04**	-0.18	4.36E-01	5.09E-01	-	transglycosylase-associated protein
EAM_1537	**2.85**	**3.79E-10**	**4.85E-08**	0.96	2.58E-02	4.95E-02	*fumA*	class I fumarate hydratase
EAM_1538	**2.70**	**1.02E-06**	**3.55E-05**	0.03	9.57E-01	9.68E-01	*ttdT*	tartrate transporter
EAM_1539	**1.45**	**3.45E-05**	**6.46E-04**	-0.75	2.88E-02	5.41E-02	-	NADH:flavin oxidoreductase
EAM_1540	**1.05**	**1.61E-05**	**3.63E-04**	-0.47	5.28E-02	8.65E-02	-	hypothetical protein
EAM_1711	**1.90**	**4.32E-04**	**4.76E-03**	-0.35	5.04E-01	5.76E-01	-	dethiobiotin synthetase
EAM_1736	**1.05**	**5.26E-04**	**5.52E-03**	0.71	1.85E-02	3.85E-02	*katA*	catalase
EAM_1775	**1.41**	**1.12E-04**	**1.63E-03**	0.93	1.04E-02	2.53E-02	*fnr*	fumarate and nitrate reduction regulatory protein
EAM_1813	**-1.03**	**2.78E-03**	**2.07E-02**	-0.76	2.78E-02	5.26E-02	-	hypothetical protein
EAM_1814	**-1.08**	**3.36E-05**	**6.36E-04**	-0.78	2.52E-03	8.10E-03	-	lipoprotein
EAM_1817	**-1.17**	**2.17E-04**	**2.72E-03**	-0.44	1.58E-01	2.14E-01	-	lipoprotein
EAM_1823	**2.35**	**4.04E-14**	**1.81E-11**	0.00	9.90E-01	9.94E-01	*palI*	sucrose isomerase
EAM_1900	**2.57**	**1.02E-09**	**1.14E-07**	0.20	6.12E-01	6.78E-01	*adhE*	aldehyde-alcohol dehydrogenase
EAM_2193	**1.09**	**5.75E-04**	**5.92E-03**	-0.21	4.99E-01	5.71E-01	*thiM*	hydroxyethylthiazole kinase
EAM_2233	**-1.22**	**5.36E-04**	**5.57E-03**	-0.88	1.12E-02	2.69E-02	-	hypothetical protein
EAM_2262	**-1.04**	**5.63E-04**	**5.82E-03**	-0.51	8.77E-02	1.31E-01	*rcsB*	capsular synthesis two-component response regulator
EAM_2269	**1.10**	**1.51E-06**	**5.03E-05**	0.68	2.95E-03	9.28E-03	-	hypothetical protein
EAM_2301	**1.11**	**1.34E-04**	**1.89E-03**	0.90	1.72E-03	5.92E-03	*ackA*	acetate kinase
EAM_2378	**-1.06**	**2.49E-03**	**1.92E-02**	-0.94	6.87E-03	1.84E-02	*mntH*	manganese transport protein
EAM_2482	**1.15**	**6.43E-06**	**1.77E-04**	0.73	4.03E-03	1.19E-02	-	aldehyde dehydrogenase
EAM_2613	**1.37**	**7.52E-04**	**7.24E-03**	0.53	1.82E-01	2.43E-01	*clpB*	chaperone ClpB
EAM_2992	**-1.49**	**1.13E-04**	**1.63E-03**	-0.09	8.05E-01	8.51E-01	*ribB*	3,4-dihydroxy-2-butanone 4-phosphate synthase
EAM_3080	**-1.07**	**2.51E-03**	**1.93E-02**	-0.16	6.41E-01	7.06E-01	-	hypothetical protein
EAM_3355	**1.33**	**4.09E-07**	**1.73E-05**	0.94	3.35E-04	1.54E-03	-	hypothetical protein
EAM_3368	**1.12**	**5.96E-04**	**6.05E-03**	0.41	2.08E-01	2.71E-01	*prtA*	zinc-binding metalloprotease
EAM_3387	**1.31**	**4.76E-08**	**3.10E-06**	0.58	1.53E-02	3.35E-02	-	cellulose synthase catalytic subunit
EAM_3388	**1.52**	**9.15E-09**	**7.45E-07**	0.40	1.22E-01	1.71E-01	-	cellulose biosynthesis protein
EAM_3389	**1.26**	**8.43E-04**	**7.86E-03**	0.14	7.08E-01	7.66E-01	-	hypothetical protein
EAM_3450	**1.09**	**1.67E-05**	**3.64E-04**	0.94	1.96E-04	9.85E-04	*gyrB*	DNA gyrase subunit B

[a] DEGs are defined as genes with greater than 2 fold-change (FC) of the CPM value and less than 0.05 of the corresponding FDR value. The $\log_2$ FC value, the P-value, and the FDR values of the DEGs are in bold font.
[b] Genes annotated as "pseudo" are pseudogenes with defective copies of protein-coding genes.

Table S2. Gene ontology (GO) enrichment analysis.

			Subtoxic condition			Toxic condition		
Expression[a]	GO ID	GO terms	Query/ Reference[b]	P-value	FDR	Query/ Reference	P-value	FDR
Upregulated	GO:0006412	translation	43/108	9.40E-20	8.90E-17	73/108	1.60E-28	3.40E-25
	GO:0019538	protein metabolic process	61/292	5.60E-12	1.30E-09	-[c]	-	-
	GO:0006091	generation of precursor metabolites and energy	22/60	2.50E-10	4.80E-08	-	-	-
	GO:0006119	oxidative phosphorylation	12/14	2.90E-12	9.20E-10	14/14	3.70E-11	1.50E-08
	GO:0044249	cellular biosynthetic process	97/760	4.50E-06	1.50E-04	-	-	-
	GO:0009058	biosynthetic process	99/796	9.90E-06	3.00E-04	-	-	-
	GO:0010467	gene expression	60/415	4.20E-06	1.50E-04	126/415	5.40E-09	1.40E-06
	GO:0044237	cellular metabolic process	135/1250	2.50E-04	4.90E-03	311/1250	1.50E-07	3.20E-05
	GO:0044238	primary metabolic process	-	-	-	304/1228	2.80E-07	4.60E-05
	GO:0006399	tRNA metabolic process	-	-	-	31/69	2.60E-07	4.60E-05
	GO:0006633	fatty acid biosynthetic process	-	-	-	11/13	3.30E-07	4.60E-05
	GO:0015985	energy coupled proton transport	8/8	1.00E-09	1.10E-07	-	-	-
	GO:0044281	small molecule metabolic process	-	-	-	-	-	-
	GO:0034220	ion transmembrane transport	8/21	8.50E-05	1.90E-03	13/21	9.50E-06	5.50E-04
	GO:0043170	macromolecule metabolic process	-	-	-	214/876	1.20E-05	6.50E-04
	GO:0006457	protein folding	7/19	3.00E-04	5.70E-03	12/19	1.60E-05	7.80E-04
	GO:0008152	metabolic process	-	-	-	369/1619	3.30E-05	1.40E-03
	GO:0009615	response to virus	5/6	1.30E-05	3.90E-04	6/6	3.20E-05	1.40E-03
	GO:0009987	cellular process	-	-	-	361/1583	3.50E-05	1.50E-03
	GO:0006099	tricarboxylic acid cycle	-	-	-	10/16	9.30E-05	3.50E-03
	GO:0002376	immune system process	5/5	2.30E-06	8.60E-05	5/5	1.80E-04	5.50E-03
	GO:0042274	ribosomal small subunit biogenesis	-	-	-	6/7	1.90E-04	5.60E-03

	GO:0042180	cellular ketone metabolic process	-	-	-	69/252	2.10E-04	6.00E-03
	GO:0046483	heterocycle metabolic process	-	-	-	-	-	-
	GO:0010608	posttranscriptional regulation of gene expression	-	-	-	9/15	3.30E-04	8.80E-03
Downregulated	GO:0009064	glutamine family amino acid metabolic process	8/36	8.40E-07	3.50E-05	-	-	-
	GO:0006935	chemotaxis	-	-	-	15/34	8.30E-06	4.60E-04
	GO:0009306	protein secretion	-	-	-	32/56	1.40E-14	2.70E-12

[a] GO enrichment analysis was conducted seperately on DEGs that were upregulated and downregulated, respectively.

[b] The ratio of the number of DEGs to the total number of genes in the GO category.

[c] "-" means missing data due to none-overpresention in the GO entry.

Table S3. Summarization of the phenotypes that have been investigated in this study and previous studies of type I membrane-associated toxins.

Host	Toxin gene	Induction level*	Intracellular ATP	Extracellular ATP	PMF	Antibiotic persistence	References
E.coli	*tisB*	Toxic	Decreased	Unknown	Collapsed	Unkown	6,7
E.coli	*tisB*	Subtoxic	Unknown	Unknown	Unknown	Increased	13
E.coli	*hokB*	Subtoxic**	Unknown	Increased	Collapsed	Increased	9,12
E.coli	*hok*	Toxic	Unknown	Unknown	Collapsed	Unknown	8
E. amylovora	*hok*	Subtoxic	Increased	Unchanged	Collapsed	Increased	This study
E. amylovora	*hok*	Toxic	Decreased	Increased	Collapsed	Unkown	This study

*The induction levels of the toxin genes in previous studies are re-categoriezed using the definition of the subtoxic and the toxic level in this study.

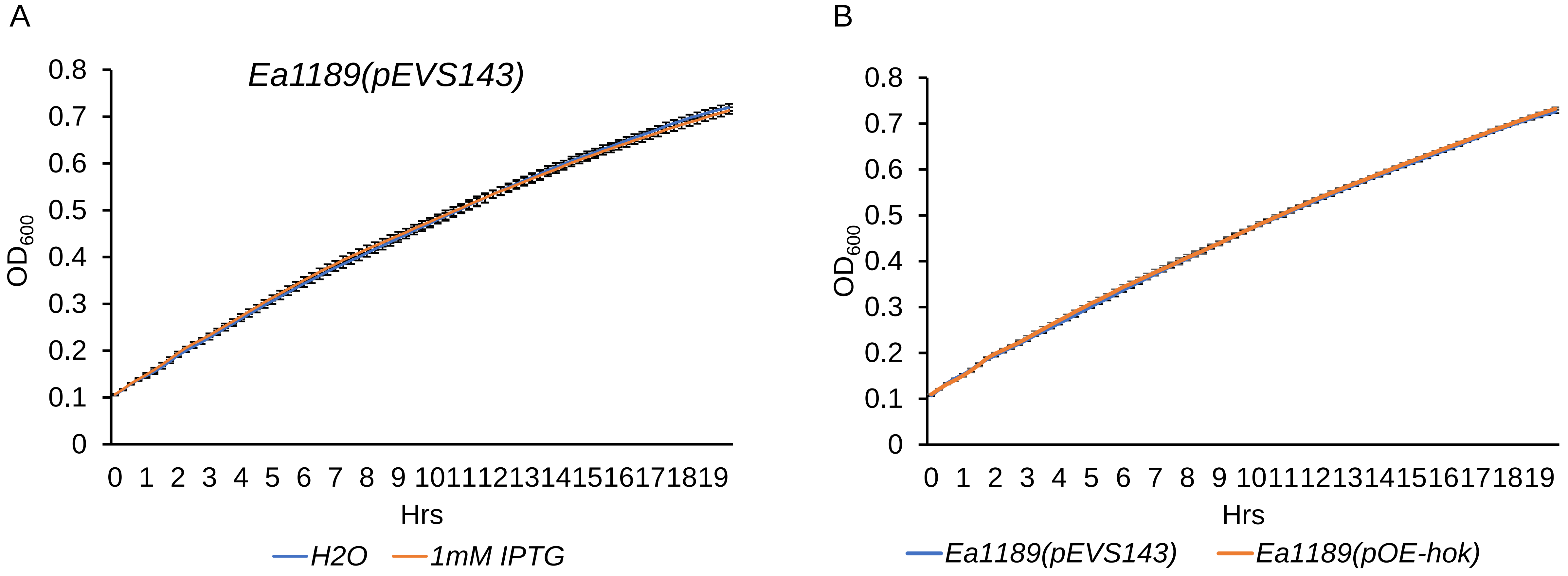

Fig. S1 Effect of 1 mM IPTG on growth of *E. amylovora* Ea1189(pEVS143) cultures (A) and effect of pOE-*hok* transformation, without any IPTG addition, on *E. amylovora* Ea1189 cultures (B). Overnight *E. amylovora* cultures were washed twice in fresh LB broth and adjusted to OD_{600}=0.1 in LB broth containing 1 mM IPTG or H_2O. Cultures in 150 µl were transferred to a 96-well plate and incubated in a Tecan spectrophotometer at 28 °C with periodic shaking. OD_{600} values were measured at 15 min intervals. Results represent the means of three biological replicates, and error bars indicate the standard deviation. The assays were done twice with similar results.

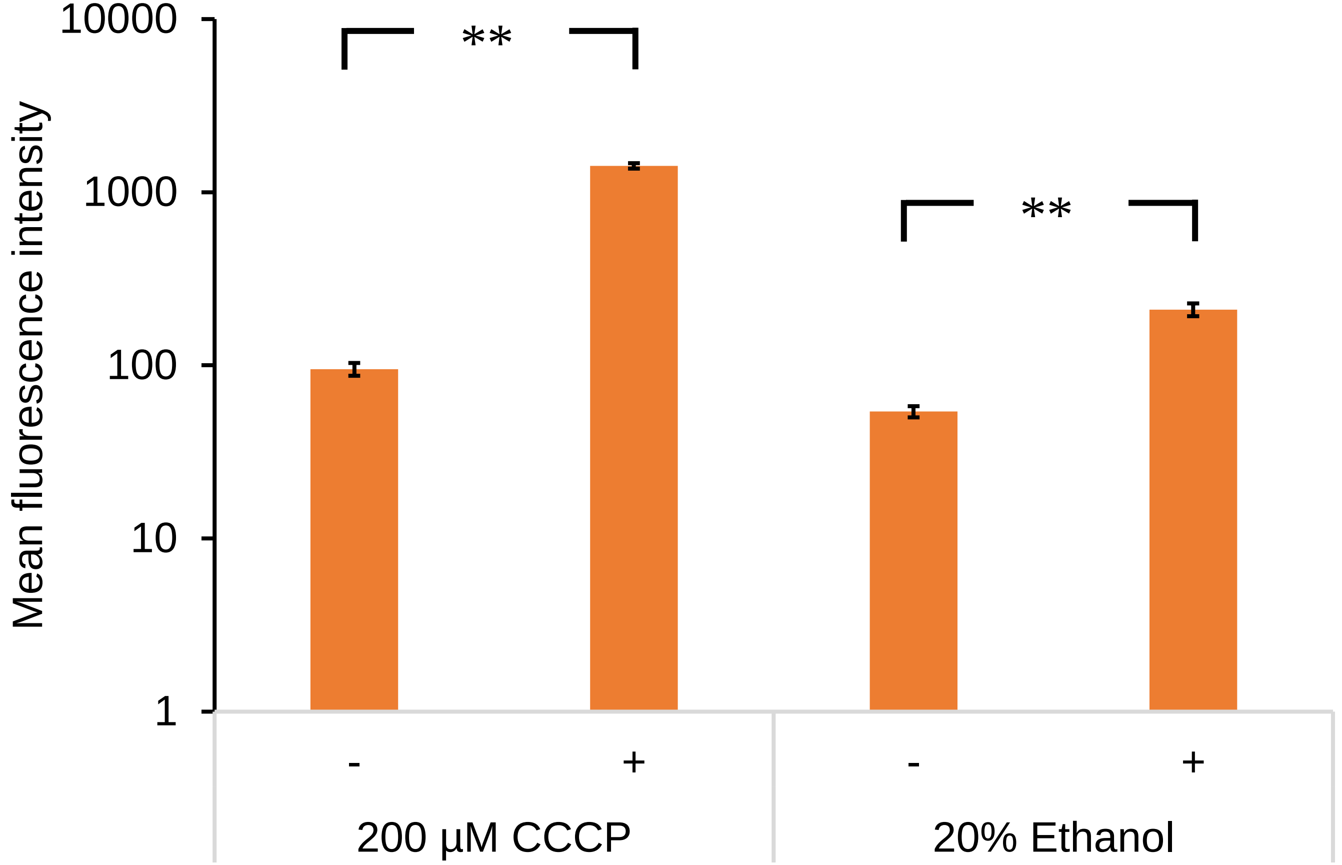

Fig. S2 Effect of 200 µM CCCP on DiBAC$_4$(3) staining and 20% Ethanol on PI staining, respectively, in *E. amylovora* Ea1189(pEVS143) cells measured using a flow cytometer. Ten thousand events were examined using a 488 nm laser and a 530/30 emission filter for cells stained by DiBAC$_4$(3) or a 561 nm laser and a 620/15 emission filter for PI staining. Results represent the means of three replications, and error bars indicate the standard deviation. Asterisk signs indicate significant differences ($P < 0.05$) using Student's t-test. The assays were done two times with similar results.

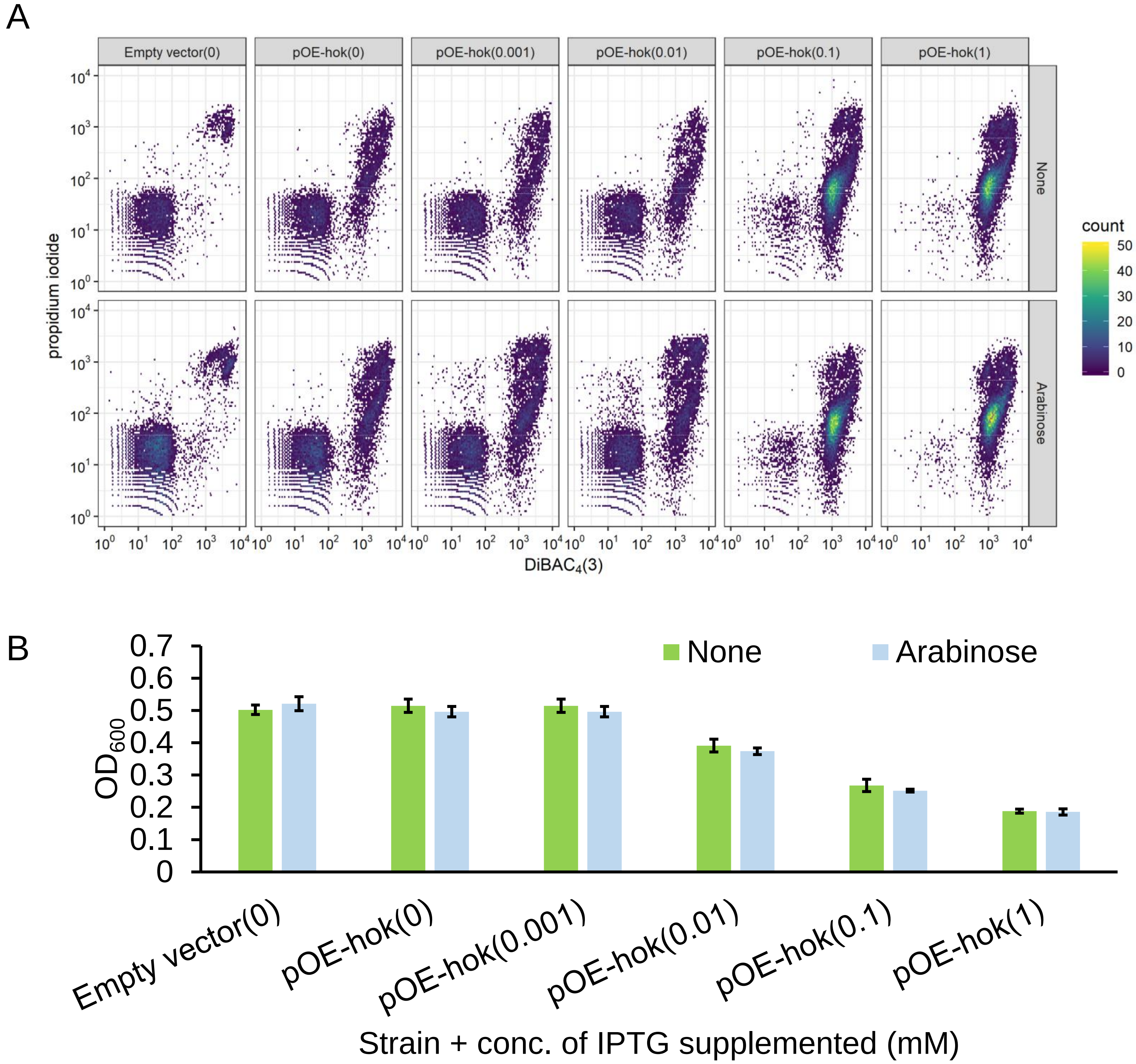

Fig. S3 Addition of arabinose does not significantly affect the proton motive force (PMF) and membrane integrity (A) or growth (B) of *E. amylovora* cultures when *hok* was induced at a subtoxic or toxic level. Arabinose at 10 mM or water was added to *E. amylovora* overnight cultures that were normalized to OD_{600}=0.2 in fresh LB broth. The concentrations of IPTG indicated in parentheses were then supplemented. The PMF and membrane integrity was determined 1 h after the IPTG addition; the OD_{600} values of the cultures were measured 4 h after the IPTG addition. Results represent the means of three biological replicates and analyzed using the Student's *t*-test with a cutoff *P*-value of 0.05 Error bars indicate the standard deviation. The assays were conducted three times with similar results.

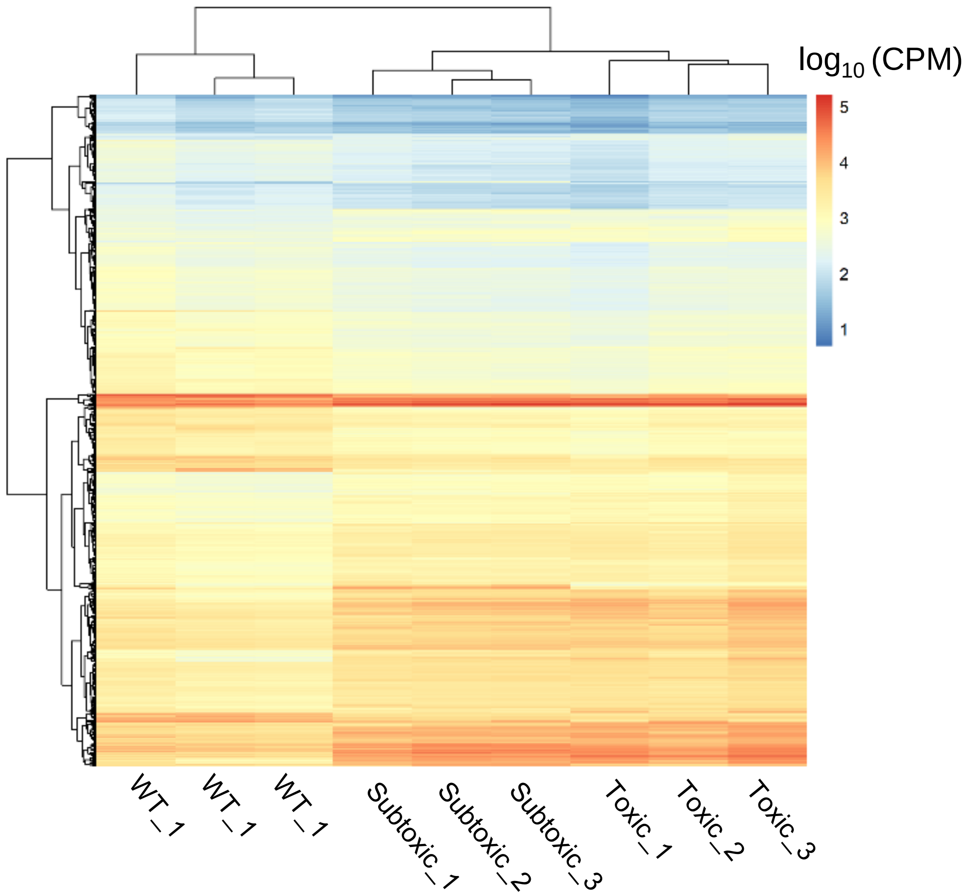

Fig. S4 A heatmap of counts per million reads (CPM) values of all genes in *E. amylovora*. Each column represents one replication of the treatment. Scale bar indicates the color key of the $\log_{10}$ CPM values.

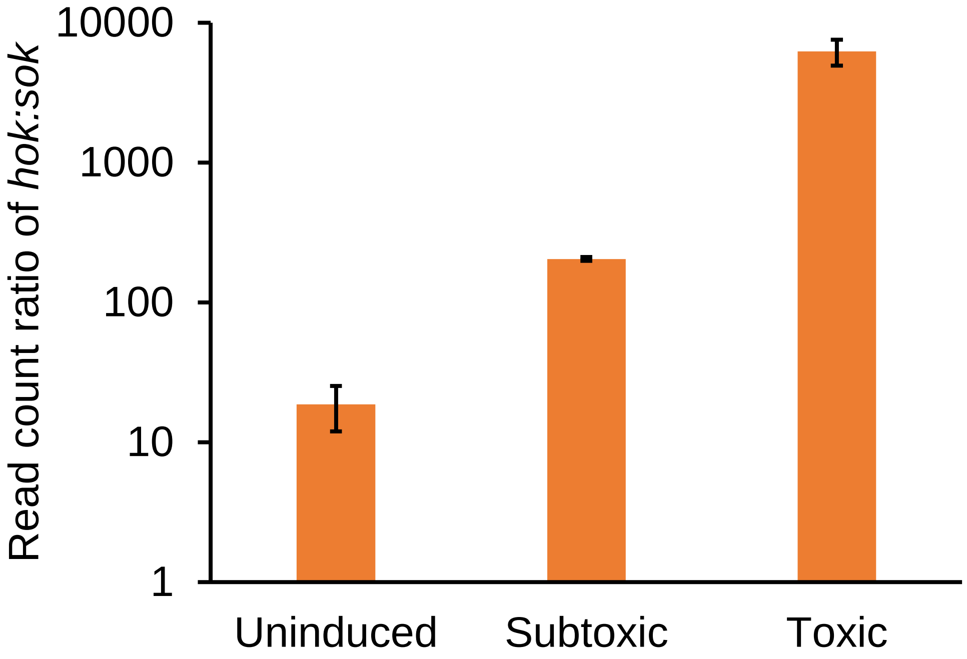

Fig. S5 Read count ratio of *hok* to *sok* in the whole transcriptomic analysis. “Uninduced”, “subtoxic”, and “toxic” indicates *E. amylovora* cells expressing *hok* at wild-type, subtoxic, and toxic levels, respectively. Error bars indicate the standard error of the mean for the three biological replications of each condition.

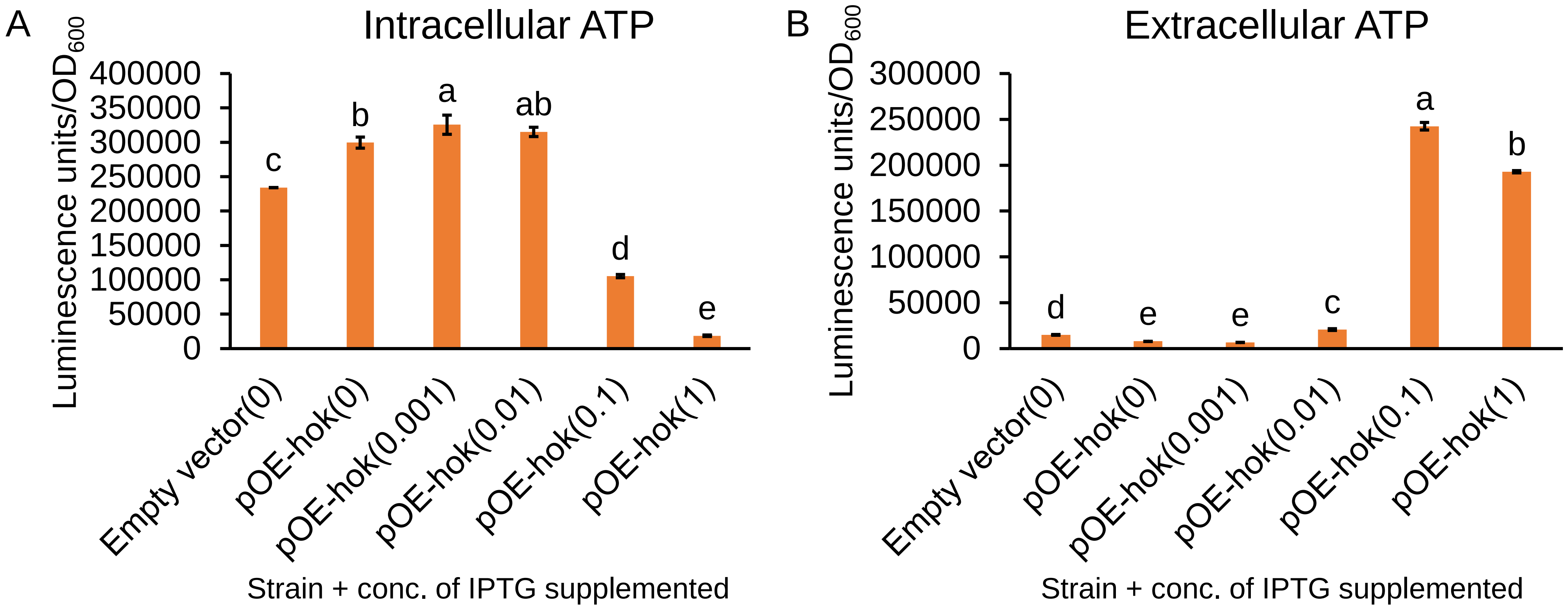

Fig. S6 Effect of *hok* induction on intracellular (A) and extracellular (B) levels of ATP in *E. amylovora*. Both extracellular and intracellular levels of ATP were simultaneously quantified using a luciferase reporter system. Results represent the means of three biological replications and error bars indicate the standard deviation. Different letters indicate significant differences ($P < 0.05$) using Tukey's HSD test. The assays were done twice with similar results.

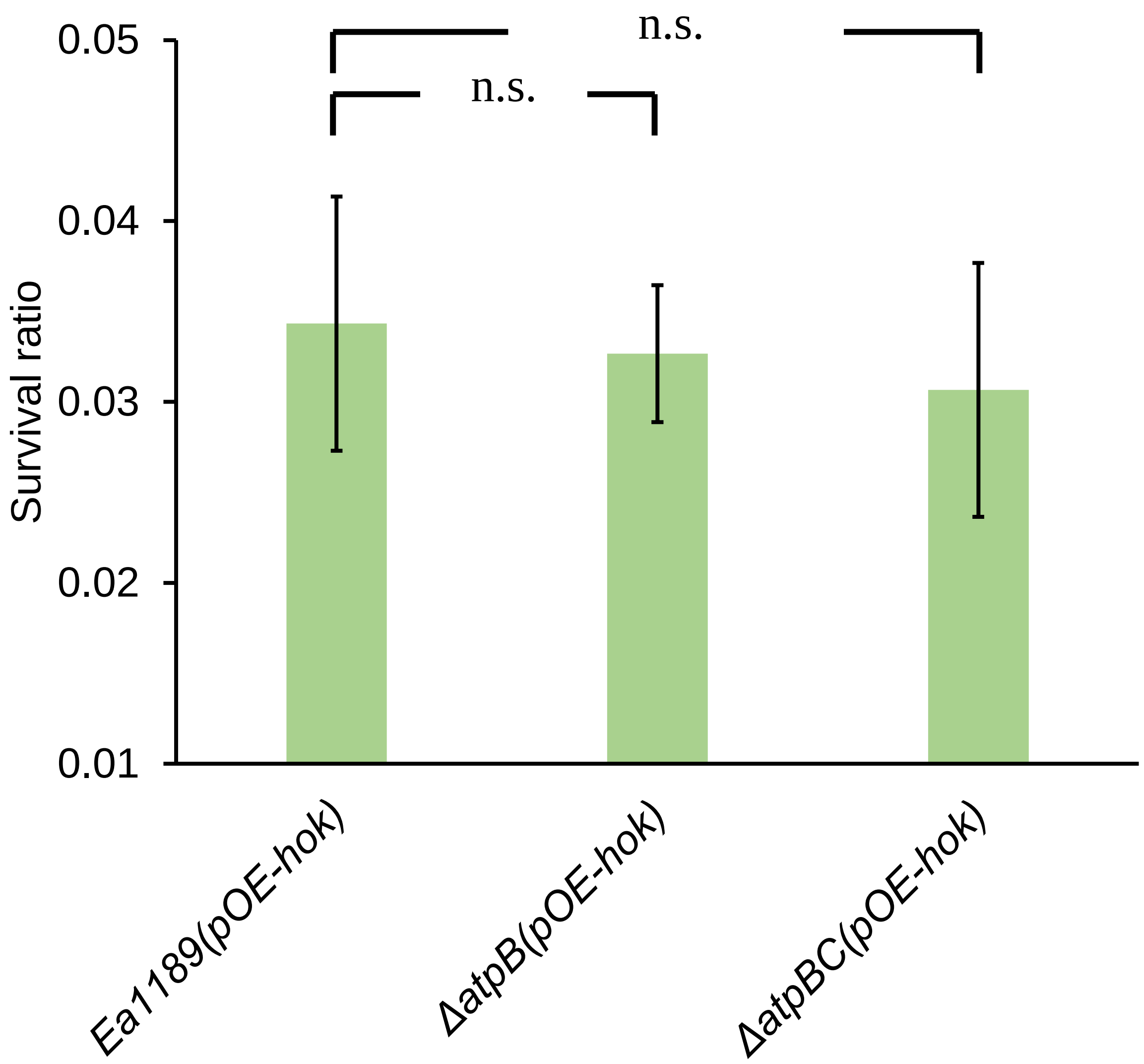

Fig. S7 Deletion of the ATP synthase gene *atpB* alone or the chromosomal region that spans the *atpB*, *atpE*, *atpF*, *atpH*, *atpA*, *atpG*, *atpD*, and *atpC* genes does not significantly affect the cell death due to induction of *hok* with 1 mM IPTG. Survival ratio was determined as the ratio of CFU/ml after the addition of 1 mM IPTG for 1 h to that before the treatment. Results represent the means of three biological replications and error bars indicate the standard deviation. “n.s.” indicates no significant difference ($P \geq 0.05$ using Student’s t-test). The assays were done three times with similar results.

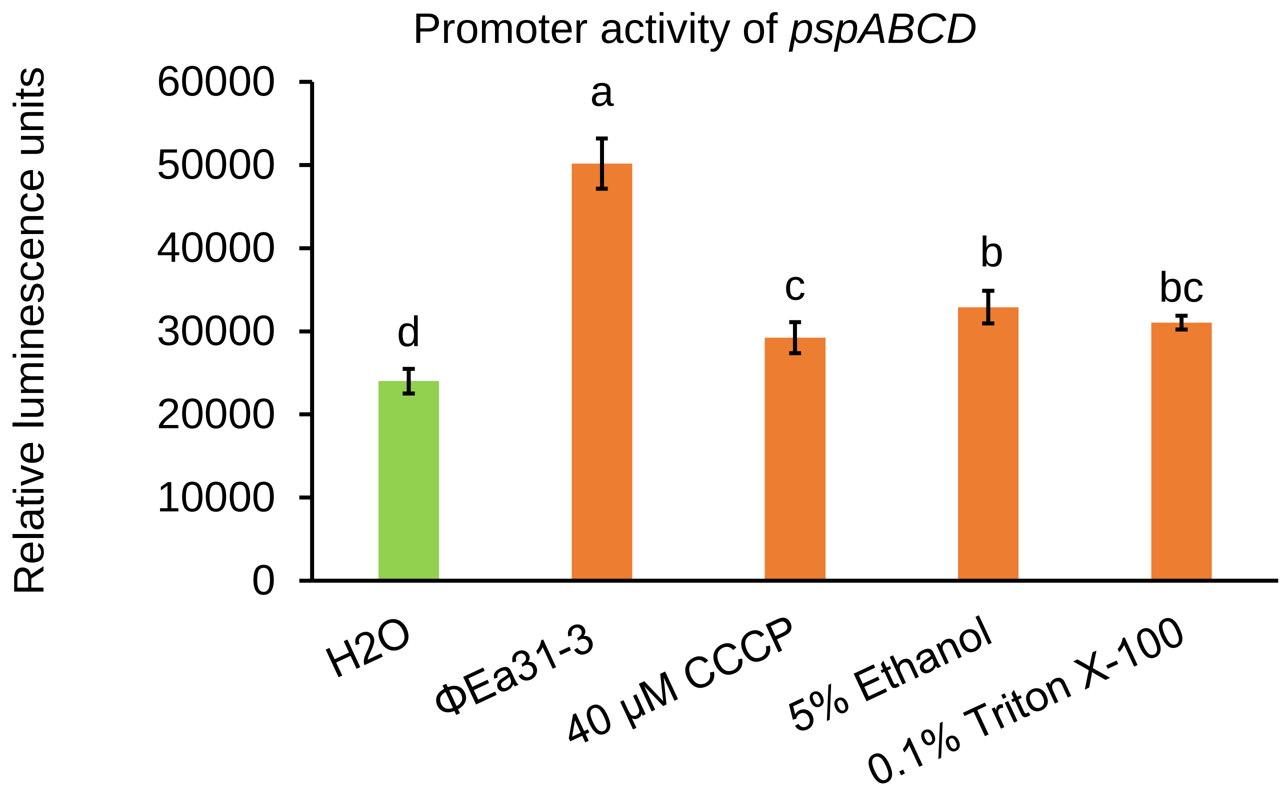

Fig. S8 Promoter activity of *pspABCD* in *E. amylovora* cells in known PMF dissipation conditions. The promoter activity of *pspABCD* was measured based on a gfp transcriptional fusion reporter system. Fluorescence was measured using a Tecan spectrophotometer plate reader. Around 10^8 PFU bacteriophage ΦEa31-3, 40 µM carbonyl cyanide-m-chlorophenylhydrazone (CCCP), 5% ethanol or 0.1% Triton X-100 was supplemented to *E. amylovora* cultures. Results represent the means of four biological replications and error bars indicate the standard deviation. Different letters indicate significant differences ($P < 0.05$) using Tukey's HSD test. For bars sharing the same letter, the difference is not statistically significant. The assays were done three times with similar results.
